# Enhancing a *de novo* enzyme activity by computationally-focused ultra-low-throughput screening†
†Electronic supplementary information (ESI) available: Additional simulation details and table of the full list of variants predicted by FuncLib. See DOI: 10.1039/d0sc01935f


**DOI:** 10.1039/d0sc01935f

**Published:** 2020-05-19

**Authors:** Valeria A. Risso, Adrian Romero-Rivera, Luis I. Gutierrez-Rus, Mariano Ortega-Muñoz, Francisco Santoyo-Gonzalez, Jose A. Gavira, Jose M. Sanchez-Ruiz, Shina C. L. Kamerlin

**Affiliations:** a Departamento de Química Física, Facultad de Ciencias , Unidad de Excelencia de Química aplicada a Biomedicina y Medioambiente (UEQ) , Universidad de Granada , 18071 Granada , Spain . Email: sanchezr@ugr.es; b Science for Life Laboratory , Department of Chemistry-BMC , Uppsala University , BMC Box 576 , S-751 23 Uppsala , Sweden . Email: lynn.kamerlin@kemi.uu.se; c Departamento de Química Orgánica , Facultad de Ciencias , Unidad de Excelencia de Química aplicada a Biomedicina y Medioambiente (UEQ) , Universidad de Granada , 18071 Granada , Spain; d Laboratorio de Estudios Cristalográficos , Instituto Andaluz de Ciencias de la Tierra , CSIC, Unidad de Excelencia de Química aplicada a Biomedicina y Medioambiente (UEQ) , University of Granada , Avenida de las Palmeras 4 , 18100 Armilla , Granada , Spain

## Abstract

Directed evolution has revolutionized protein engineering. Still, enzyme optimization by random library screening remains sluggish, in large part due to futile probing of mutations that are catalytically neutral and/or impair stability and folding. FuncLib is a novel approach which uses phylogenetic analysis and Rosetta design to rank enzyme variants with multiple mutations, on the basis of predicted stability. Here, we use it to target the active site region of a minimalist-designed, *de novo* Kemp eliminase. The similarity between the Michaelis complex and transition state for the enzymatic reaction makes this system particularly challenging to optimize. Yet, experimental screening of a small number of active-site variants at the top of the predicted stability ranking leads to catalytic efficiencies and turnover numbers (∼2 × 10^4^ M^–1^ s^–1^ and ∼10^2^ s^–1^) for this anthropogenic reaction that compare favorably to those of modern natural enzymes. This result illustrates the promise of FuncLib as a powerful tool with which to speed up directed evolution, even on scaffolds that were not originally evolved for those functions, by guiding screening to regions of the sequence space that encode stable and catalytically diverse enzymes. Empirical valence bond calculations reproduce the experimental activation energies for the optimized eliminases to within ∼2 kcal mol^–1^ and indicate that the enhanced activity is linked to better geometric preorganization of the active site. This raises the possibility of further enhancing the stability-guidance of FuncLib by computational predictions of catalytic activity, as a generalized approach for computational enzyme design.

## Abstract 2

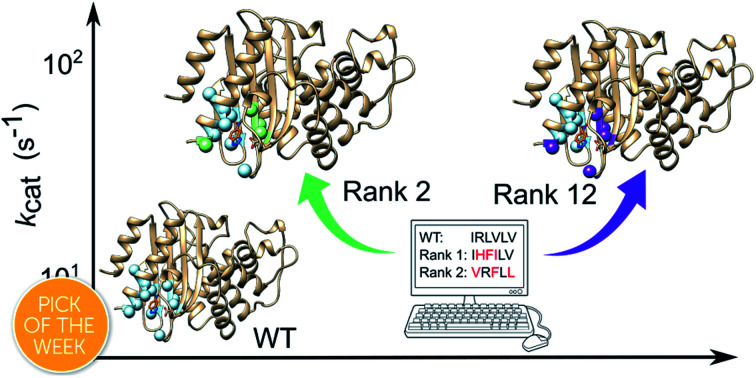
 *De novo* enzymes capable of efficiently catalysis of a non-natural reaction are obtained through minimalist design plus computationally-focused variant library screening.

## Introduction

Enzymes are green catalysts with unmatched catalytic proficiencies,^1^ and with widespread applications in biotechnology as extracellular catalysts for a host of (bio)chemical processes, from organic synthesis to developing new pharmaceuticals, biofuels, or bioremediation agents, to name but a few examples (see *e.g.*ref. 2 and 3 for an overview). To be able to efficiently control the physicochemical properties of enzymes in a tailored fashion is therefore a problem with major economic implications, leading to extensive research effort in this direction.^4^ However, natural enzymes have had millions of years to evolve to their modern catalytic efficiencies, and therefore mimicking this process whether *in vitro* or *in silico* is a non-trivial undertaking, in particular due to the immensity of the sequence space that needs exploring, and the very high frequency of catalytically detrimental mutations.^5^,^6^ Directed evolution revolutionized experimental protein engineering efforts, by vastly expanding the sequence space accessible to protein engineers by several orders of magnitude, with low overhead.^7^–^9^ Despite its many advantages, as a caveat, directed evolution is time-consuming, typically requiring many rounds of medium or high-throughput screening to achieve suitable levels of enzyme catalysis from a starting, low seed level.^10^ Nevertheless, it has facilitated the development of a wide diversity of biotechnological applications of proteins.

Recent years have seen an explosion of interest also in computational enzyme design,^11^–^14^ propelled in large part by early successes in *de novo* enzyme design through grafting computationally designed active site models onto natural protein scaffolds (*e.g.*ref. 15–17, among others). We note, however, that while impressive, this approach typically generates enzymes with only modest catalytic activities, which again require many rounds of directed evolution before reaching catalytic efficiencies^10^,^18^ that are comparable to naturally occurring enzymes.^19^

In light of the above, the use of computation to focus and speed up directed evolution is of considerable interest. Indeed, there have been substantial advances in this field, with many new screening approaches being put forward, based on sequence, structural or even dynamical information gained from simulations (see *e.g.*ref. 20–30). In addition, machine learning shows great promise as a screening tool in enzyme design studies.^31^–^34^ Still, the best engineered enzymes, with catalytic efficiencies comparable to natural enzymes, are more often the results of intensive directed evolution efforts starting from low-activity rational designs.^10^,^18^

**Fig. 1:**
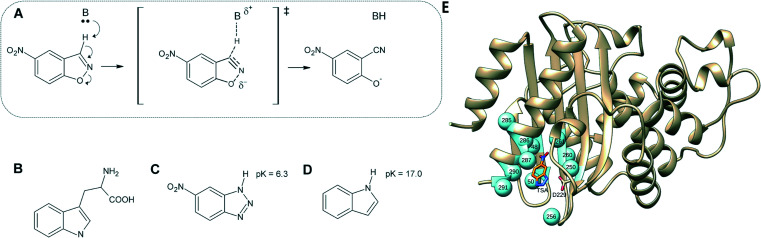
(A) Kemp elimination of 5-nitrobenzisoxazole showing a proposed transition state structure. For comparison, shown here are also the structures of (B) tryptophan, (C) a transition-state analog and (D) indole. (E) 3D-structure of the background GNCA4-WT *de novo* enzyme (PDB ID: ; 5FQK,^35^ referred to throughout as GNCA4-WT), showing both the position of the bound transition analogue, as well as the key residues we targeted using FuncLib (shown as spheres). Panels (A–D) were originally published in ref. 35. Reproduced here with permission from ref. 35. Copyright 2017, the authors. Published under a CC-BY license (; http://creativecommons.org/licenses/by/4.0/).

The sluggishness of the common directed evolution procedures has to do, at least in part, with the fact that most variants in a random library with a substantial mutational load will include mutations that are deleterious in terms of fundamental protein biophysical properties, such as stability and folding. FuncLib^28^ is a novel automated method for designing multipoint mutations at enzyme active sites by combining phylogenetic analysis and Rosetta design calculations. FuncLib does not *per se* predict mutations that enhance catalysis, but rather suggests variants with multiple mutations that generate stabilizing interacting networks at the active site, thus focusing the search to safe regions of the sequence space. Furthermore, FuncLib can be used to target regions that are expected to be relevant for catalysis, thus avoiding the inefficiency associated with probing catalytically neutral mutations. We note here that while one might intuitively expect trade-offs between catalytic activity and stability, this is not necessarily the case *a priori*: that is, it has been experimentally demonstrated that it is possible to enhance stability through either engineering^36^ or directed evolution,^37^ without compromising activity. Here, we apply the FuncLib approach to the enhancement of the activity of a *de novo* enzyme activity previously generated by minimalist rational design.^35^ Specifically, we recently demonstrated that a simple hydrophobic-to-ionizable residue substitution (Fig. 1) is sufficient to generate a *de novo* active site capable of highly proficient Kemp eliminase activity for the cleavage of 5-nitrobenzisoxazole in Precambrian β-lactamases obtained by ancestral inference,^35^ with the best of our designs (*k*_cat_/*K*_M_ ∼ 5 × 10^3^ M^–1^ s^–1^ and *k*_cat_ ∼ 10 s^–1^ at alkaline pH) showing catalytic proficiencies only two orders of magnitude lower than the best designed Kemp eliminase obtained through iterative design followed by 17 rounds of directed evolution.^38^

There are a number of reasons Kemp elimination is particularly attractive as a model system for *de novo* enzyme design studies. (1) It provides a simple activated model for proton abstraction by carbon, (2) as a non-natural reaction it means that no natural enzyme has evolved to catalyse this reaction, reducing the risk of contamination from natural enzymes, and (3) for historical reasons, Kemp elimination has often been used as a benchmark for enzyme (and other catalyst) design studies,^15^,^35^,^38^–^47^ providing extensive examples of designed constructs against which to compare our engineered β-lactamases. Certainly, Kemp elimination is a facile reaction that requires a simple catalytic machinery (essentially, a catalytic base to abstract the proton). However, and as a relevant point in the context of this work, it is difficult to generate high levels of Kemp eliminase activity because the transition state is so similar to the reactant state, both in terms of structure and in terms of charge distribution.^48^ That is, structurally, the overall geometry of both the substrate and transition state are similar. Therefore, moving from the substrate to the transition state does not bring about a large change in the spatial arrangement of the interacting moieties that could be used as a basis for the preferential stabilization of the transition state. In addition, charge build-up on the ring oxygen at the transition state (Fig. 1) is highly delocalized into the aromatic ring of the substrate.^48^–^50^ Therefore, it is highly challenging to use improved transition state stabilization or manipulate active site polarity as a means to achieve substantial gains in catalysis. As an illustration of this, Hilvert and coworkers^50^ have recently explored in detail the contribution of oxyanion hole stabilization to the highly proficient Kemp eliminase, HG3.17, and find the contribution of a key residue forming this oxyanion hole, Gln50, to be only modest, likely reflecting charge delocalization at the transition state.

Use of FuncLib allows us to consider the effect of mutations at 11 positions simultaneously, thus avoiding problems caused by epistasis which can lead to unpredictable (non-additive) effects on enzyme activity.^51^–^53^ Remarkably, we find that screening of just 20 FuncLib predicted variants leads to substantial enhancement of our previous best Kemp eliminase. That is, experimental validation of the twenty best scoring FuncLib predictions through biochemical and structural analysis allows us to identify 4 variants with significantly enhanced catalytic efficiency and improved turnover number, the best of which reach catalysis levels (*k*_cat_/*K*_M_ of ∼2 × 10^4^ M^–1^ s^–1^ and *k*_cat_ of ∼10^2^ s^–1^) for the cleavage of 5-nitrobenzisoxazole that compare favourably with that of naturally occurring enzymes.^19^ In addition, we demonstrate that the empirical valence bond (EVB) approach^54^ can reproduce the experimental free energy barriers for the optimized eliminases to within ∼2 kcal mol^–1^, raising the possibility of further enhancing the stability-guidance of FuncLib on the basis of EVB-based computational predictions of catalytic activity. Overall, we demonstrate a simple computational protocol with tremendous potential for biocatalysis.

## Materials and methods

### Initial screening using FuncLib

Initial design was performed using the FuncLib webserver (http://funclib.weizmann.ac.il/), as described in ref. 28. As our starting point, we selected all amino acids in close contact with the substrate for randomization by FuncLib, comprising of 11 starting positions (V48, D50, I250, R256, L260, V261, L285, V286, V287, W290 and H291, see Table S1†). The calculations were performed on Chain A of the crystal structure of the GNCA4-W229D/F290W variant (PDB ID: ; 5FQK,^35^ henceforth referred to as GNCA4-WT), with the transition state analog, 6-nitrobenzotriazole, retained in the calculation, and the His tag removed. The multiple sequence alignment was performed using the default parameters, and the top twenty ranked designs based on their stability score were retained for further experimental and computational analysis.

### Empirical valence bond simulations

The empirical valence bond (EVB) approach^54^ has been extensively used to successfully study enzyme catalysis in general,^55^,^56^ and Kemp elimination in particular.^48^,^57^–^59^ In this context, we recently used the EVB approach to study the evolution of multiple active site configurations^59^ in the *de novo* designed Kemp eliminase, KE07.^15^ In the present work, we follow the protocol presented in ref. 59. Our EVB simulations were performed using a simple two-state EVB model, describing the reactant and product states for the Kemp elimination reaction, with the side chain of D229 and the substrate included in the EVB region. All other residues were treated fully classically using the OPLS-AA force field.^60^,^61^ All simulations were performed using the *Q* simulation package, version 5.10,^62^ and a description of valence bond states and all EVB parameters used in the simulations are provided in the ESI of ref. 59.

EVB simulations were performed of the Kemp elimination reaction catalyzed by the GNCA4-WT β-lactamase, a series of additional single active site mutations of this variant used for calibration of the EVB simulations (G62S, A146G, A173V, L265Q, R256K, R256A), as well as the top-twenty ranked mutations predicted by the FuncLib web-server, based on both the structural predictions from FuncLib, and, where available, also crystal structures for comparison (for the three variants characterized in this work). Simulations of the GNCA4-WT variant were performed using the PDB ID: 5FQK,^35^ and the best hits from the FuncLib webserver were simulated based on the PDB structures provided by FuncLib^28^ with the substrate. The structures of all other variants were generated using SCWRL4.^63^ In all cases, the substrate 5-nitrobenzisoxazole was manually placed in the active site in the position of the transition state analogue 5(6)-nitrobenzotriazole present in the crystal structure. Missing residues at the C- and N-termini of the protein were ignored for simplicity, and the first residue of the His-tag present in the initial crystal structure was retained for consistency (this was also the case for the FuncLib calculations).

The entire system was then solvated in a 23.5 Å spherical droplet of TIP3P water molecules,^64^ centred on the CG atom of D229, and subject to surface-constrained all-atom solvent (SCAAS) boundary conditions.^65^ The system was modelled using a multi-layer approach standard to such simulations in which all atoms within the inner 85% of the water droplet are allowed to move freely, the atoms in the external 15% of the droplet are restrained to their crystallographic positions using a 10 kcal mol^–1^ Å^–2^ harmonic positional restrained, and all atoms outside the droplet are fixed at their crystallographic positions using a 200 kcal mol^–1^ Å^–2^ harmonic position restraint. Only those ionizable residues that fall within the mobile region (inner 85%) of the simulation sphere were ionized during the simulations, all other ionizable residues outside the mobile region were kept in their charge neutral states to avoid instabilities introduced by having charges located outside the explicit simulation sphere. Protonation states of ionizable residues within the explicit simulation sphere, as well as histidine protonation patterns (both of which were validated by PROPKA 3.1 (ref. 66) and visual inspection), can be found in Table S2.†

All systems were subjected to an initial 3 ps minimization at 1 K using a 0.1 fs stepsize, in order to remove bad contacts in the system after solvation. During this simulation time, a 200 kcal mol^–1^ Å^–2^ harmonic restraint was placed on all protein and substrate atoms in the simulation to restrain them to their crystallographic positions. The step size was then increased to 1 fs for the remainder of the simulations (both equilibration and subsequent EVB simulations), and the temperature was gradually increased from 1 to 300 K while simultaneously dropping the harmonic restraints from 200 to 0.5 kcal mol^–1^ Å^–2^ on only the atoms in the EVB region (not taking into account the additional restraints on atoms outside the inner 85% of the water droplet). Once the system had reached 300 K, the system was subjected to a further 20 ns of equilibration. Each equilibration was performed ten times, with ten different sets of initial velocities, leading to 200 ns of equilibration time per system, and 5.4 μs of equilibration time over all systems considered in this work. The corresponding backbone root mean square deviations are shown in Fig. S1–S3.†

For each system, the endpoints of the ten equilibration runs were then used as starting structures for subsequent EVB simulations, with three additional equilibration runs of 500 ps in length being performed from each of these starting points, using new random velocities, in order to generate 30 discrete starting points for EVB simulations of each system. The EVB free energy perturbation/umbrella sampling (EVB-FEP/US) calculations were performed in 51 individual mapping frames of 100 ps simulation length each, leading to a total of 5.1 ns simulation time per individual EVB trajectory, 153 ns simulation time per system, and 4.590 μs of equilibration time over all systems considered in this work. The EVB parameters were calibrated using the uncatalyzed background reaction in aqueous solution as a baseline, as described in ref. 59. The same calibration as in our previous work^59^ was used in the present study, and no new calibration was performed here with all EVB parameters used in this work presented in the ESI of ref. 59.

All simulations were performed using the Berendsen thermostat^67^ with the leapfrog integrator, and with the solute and solvent coupled to individual heat baths. The bonds to hydrogen atoms were constrained using the SHAKE algorithm.^68^ Cut-offs of 10 and 99 Å were used for the calculation of non-bonded interactions involving the protein and water molecules and the EVB region respectively (effectively no cut-off for the latter), and electrostatic interactions for all atoms falling beyond this cut-off were approximated using the local reaction field approach.^69^ The non-bonded pairlist was updated every 30 fs. All simulation analysis was performed using the *Q*Calc module of *Q*,^62^ and all structural analysis was performed using VMD version 1.9.3.^70^ For full simulation details, see ref. 59.

### Protein expression, purification and library screening

The different β-lactamase variants studied in this work were purified using procedures previously described in detail in ref. 35 and 71. Briefly, genes for the His-tagged proteins were cloned into a pET24 vector with kanamycin resistance, were cloned into *E. coli* BL21(DE3) cells, and the proteins were purified by NTA affinity chromatography. Stock solutions for activity determinations and physicochemical characterization were prepared by exhaustive dialysis against the desired buffer.

Mutagenized libraries for screening studies were generated by error-prone PCR using the GeneMorph II Random Mutagenesis kit (Agilent) and transformed into *E. coli* Bl21 (DE3) and individual colonies were picked and grown in 96-well plates. The Kemp eliminase activity of ∼500 variants were assayed with 5-nitrobenzisoxazole (0.25 mM) in 96-well plates. This primary screening served to select variants that were subsequently prepared and tested on pure form. In most cases, this secondary screening implied the determination of profiles of activity *versus* substrate concentration.

### Stability determination

Thermal denaturation of the different β-lactamase variants studied in this work was studied using differential scanning calorimetry at a scan rate of 200 K per hour in HEPES 10 mM, 100 mM NaCl, pH 7 following protocols that have been previously described in detail.^71^ A single transition was observed in thermograms of heat capacity *versus* temperature. Denaturation temperature values correspond to the maximum of the calorimetric transition.

### Activity determination

Determination of Kemp elimination activity were carried out at 25 °C HEPES 10 mM or 10 mM sodium phosphate (in all cases with 100 mM NaCl), depending on the pH range, as has been previously described in ref. 35. Experiments were routinely carried out in the presence of acetonitrile to increase the solubility of the substrate and expand its experimental concentration range, thus facilitating the detection of curvature in Michaelis plots and, therefore, the reliable determination of turnover numbers. 5% acetonitrile was used in most cases, although experiments with higher and lower acetonitrile contents were also performed (see the Results and discussion for details). It is to be noted that, even in those cases in which no acetonitrile is added on purpose, a small amount of the cosolvent is present because the stock solution of the substrate is prepared in acetonitrile. The approximate substrate ranges used depend on acetonitrile concentration, reflecting the substrate solubility (Table S3†).

Product formation in activity determinations was followed by measuring the absorbance at 380 nm and an extinction coefficient of 15 800 M^–1^ cm^–1^ was used to calculate rates. All measurements were corrected by a blank performed under the same conditions. This is particularly critical at basic pH values, where catalysis by the hydroxyl anions may lead to substantial blank values. Still, we made sure that the level of enzyme catalysis was significantly above the blanks, even at the more alkaline pHs studied.

Catalytic parameters were determined from the fit of the Michaelis–Menten equation to the experimental rate *vs.* substrate concentration profiles. As mentioned above, solubility limits the experimentally available substrate concentration range, making it essentially impossible to experimentally reach saturation. This is, in fact, a common occurrence in studies of Kemp eliminases, and should not prevent the determination of reasonable estimate of the turnover number, *k*_cat_, provided that significant curvature is observed in the experimental Michaelis plots. For instance, the *k*_cat_ value of the best Kemp eliminase reported to date^38^ (∼700 ± 60 s^–1^) was determined from the analysis of a Michaelis plot in which saturation was not achieved and only moderate curvature was observed, as is apparent in Fig. 2C of ref. 38, and similar curvatures are seen in most Michaelis plots reported here. Still, in order to ensure that the catalytic rate enhancements reported here are not artefactual, we have performed an extensive amount of experimental work under different conditions, including at different pH and acetonitrile concentrations, to allow for increased ranges of substrate concentration. The catalytic enhancements reported here are consistent over this variety of conditions.

**Fig. 2:**
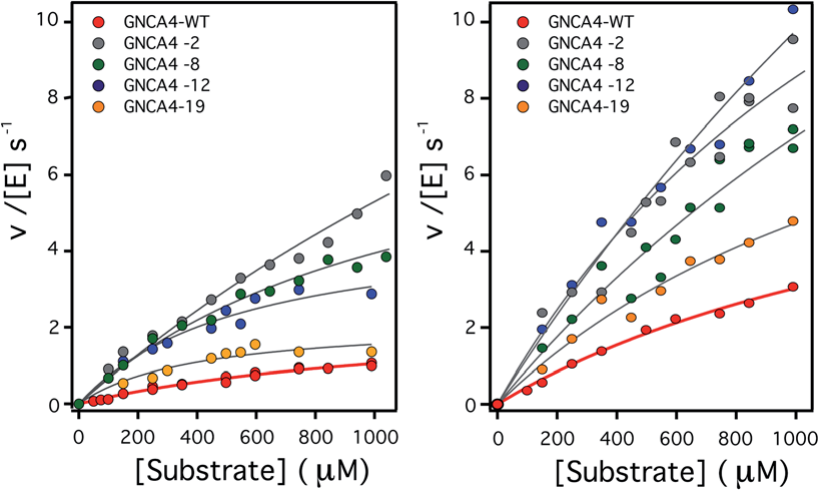
Plots of Kemp eliminase activity *vs.* substrate concentration at (left) pH 7 and (right) pH 8.4. Activities were measured here for the background protein (GNCA4-WT) and for the 4 variants that display substantially enhanced catalysis at both pH values. Michaelis plots for all the top 20 variants from the FuncLib prediction can be found in Fig. S5.† The lines are the best fits of the Michaelis–Menten equation.

### Crystallization, data collection and structure determination

In order to obtain single crystal structures of the three variants of the GNCA4 β-lactamases of interest to this work, we followed a similar protocol already described elsewhere.^35^ The three proteins were subject to crystallization assays by the capillary counterdiffusion techniques^72^ and by vapor-diffusion (VD) using the hanging drop set-up. We prepared a small screening around the known successful conditions previously used to crystallized GNCA4 and GNCA4-WT variants.^35^ In brief, for counterdiffusion experiments, each protein was concentrated to 23–25 mg mL^–1^, loaded in capillaries of 0.3 mm inner diameter and confronted to 5 M sodium formate in the pH range of 4.0 to 9.0. For VD 1 μL of protein solution was mixed with the reservoir, in a 1 : 1 ratio, and equilibrated against 500 μL of each precipitant cocktail (4 M sodium formate in the pH range of 4.0 to 9.0). The best-looking crystals of GNCA4-2 & GNCA4-12 were obtained at pH 4.0 using the counterdiffusion technique, while in the case of GNCA4-19, they grew at pH 7.0 in hanging drop.

Crystals were extracted from the capillary or fished directly from the drop, subject to cryo-protection by the equilibration with 15% (v/v) glycerol prepared in the mother liquid, with or without 1 mM of the transition-state analogue (5)6-nitrobenzotriazole (ST), flash-cooled in liquid nitrogen and stored until data collection. Crystals were diffracted at the XALOC beamline of the Spanish synchrotron light radiation source (ALBA, Barcelona). Data were indexed and integrated with XDS^73^ and scaled with SCALA^74^ of the CCP4 program suite.^75^ Molecular replacement was performed in Phaser,^76^ using the coordinates of GNCA4-WT (PDB ID: ; 5FQK 35) as the search model. Refinement was initiated with the phenix.refine^77^ module of the PHENIX suite,^78^ followed by manual building and water inspection in Coot.^79^ The final refinement of ligand coordinates, B-factors and occupancies was achieved following several cycles of refinement including Titration–Libration–Screw (TLS) parameterization. The final model coordinates were verified with Molprobity.^80^ The resulting coordinates and the experimental structure factors have been deposited in the Protein Data Bank^81^ (PDB IDs: ; 6TY6, ; 6TXD and ; 6TWW, for GNCA4-2, GNCA-12 and GNCA-19, respectively), and the corresponding crystallographic data statistics are provided in Table S4.†

## Results and discussion

### Attempting to increase *de novo* enzyme activity through random library screening

We previously used a minimalist approach (based on 1–2 mutations) to generate a completely new active site for Kemp elimination in ancestral β-lactamase scaffolds. We first attempted to enhance the activity level of our best *de novo* Kemp eliminase through using standard library screening procedures. A library of variants with random mutations and average mutational load of 3–5 mutations was prepared and 522 clones were tested, as we have described in the Materials and methods. The corresponding plot of activity relative to background *vs.* clone ranking is shown in Fig. S4.†

Of these clones, about 300 showed greatly diminished activity levels, suggesting that the encoded proteins may have failed to fold properly. We randomly chose 4 of these clones for protein preparation and, as expected, we found essentially no soluble protein. We also prepared the proteins for the top 10 clones shown in Fig. S4.† In the primary screening, these clones showed activity levels about twice or higher than that of the background variant. However, of these clones, only one was confirmed as a real positive in secondary screening carried out with the purified protein (Table 1). The corresponding variant included 6 mutations, with catalytic parameters that were only about two-fold higher than those of the background enzyme.

**Table 1:** Catalytic efficiencies and denaturation temperatures at pH 7 for the background GNCA4-WT variant, and the top 10 clones of the random library screening shown in Fig. S4^*a*^

Clone	*k*_cat_/*K*_M_ (M^–1^ s^–1^)	*T*_M_ (°C)
GNCA4-WT	3047 ± 282	80
3C11	608 ± 68	77
4B4	1770 ± 126	81
8F11	**5980 ± 117**	**80**
6D5	2476 ± 420	81
7C1	600 ± 56	72
8E12	2222 ± 167	70
6A12	1036 ± 159	79
7D1	1880 ± 155	67
2H4	2280 ± 146	ND
5H8	2066 ± 67	64

^*a*^The values in this table reflect secondary screening performed after purification of the corresponding proteins. Denaturation temperatures (*T*_M_) were derived from differential scanning calorimetry, and the catalytic parameters were obtained from fitting the Michaelis–Menten equation to the experimental rate *vs.* substrate concentration profiles. Note that only one of the variants (clone 8F11) shows mildly enhanced catalytic activity in this secondary screening. The *k*_cat_/*K*_M_ for the GNCA4-WT was originally presented in ref. 35. The *k*_cat_/*K*_M_ and *T*_M_ of the most efficient clone (8F11) is highlighted in bold. All kinetic measurements were performed at 25 °C.

In order to determinate whether this rather moderate enhancement was due to cancelation between enhancing and deleterious effects of the different mutations, we determined the effect of the single mutations on Kemp eliminase activity. However, no strong cancellation was found (Table 2). Overall, these results highlight the low efficiency and limited enhancements that are typical of non-focused library screening. There is little doubt, of course, that a directed evolution experiment would eventually lead to substantial enhancements in activity, but this will likely require many rounds of library preparation and screening, and also the focus of this study is the extent to which computational approaches can be used to enhance enzyme activity *in lieu* of (otherwise more costly) directed evolution experiments.

**Table 2:** A comparison of calculated and experimental activation free energies for the Kemp elimination of 5-nitrobenzisoxazole by the GNCA4-WT β-lactamase and a series of active site mutants^*a*^

Variant	*k*_cat_	*K*_m_	*k*_cat_/*K*_m_	Δ*G*‡exp	Δ*G*‡calc
GNCA4-WT (no His-tag)	2.6 ± 0.44	1.5 ± 0.4	1705 ± 139	16.7	16.2 ± 0.1
G62S	3.64 ± 0.83	1.25 ± 0.45	2911 ± 401	16.7	16.3 ± 0.2
A146G	5.44 ± 0.77	2.34 ± 0.44	2328 ± 112	16.5	16.5 ± 0.2
A173V	3.78 ± 0.19	1.53 ± 0.12	2464 ± 62	16.7	16.9 ± 0.3
L265Q	4.4 ± 1.01	1.8 ± 0.58	2447 ± 242	16.6	16.7 ± 0.2
R256K	6.13 ± 1.76	3.2 ± 1.1	1542 ± 369	16.4	16.9 ± 0.2
R256A	4.80 ± 1.40	4.7 ± 1.6	875 ± 15	16.5	16.6 ± 0.3

^*a*^The GNCA4-WT β-lactamase, which is used as the baseline for our study, is referred to in this table as “wild-type” (“GNCA4-WT”). Note that this data for the “wild type” was measured without a His-tag in ref. 35, which accounts for the small difference with the data given in Table 1 (taken also from ref. 35). Kinetic measurements were performed as described in the Methodology section, and *k*_cat_, *K*_M_, and *k*_cat_/*K*_M_ values are provided in s^–1^, mM, and M^–1^ s^–1^, respectively. Δ*G*‡exp and Δ*G*‡calc denote the experimental and calculated activation free energies for these enzymes, in kcal mol^–1^. Δ*G*‡exp was derived from *k*_cat_ using transition state theory, and Δ*G*‡calc is shown as averages and standard error of the mean over thirty individual EVB trajectories per system. All the values in this table were measured at pH 7 with no acetonitrile (other than the small amount coming from the substrate stock solution). All kinetic measurements were performed at 25 °C.

### Generation and preliminary assessment of FuncLib predictions

As described in ref. 28, the purpose of FuncLib is to be used to design a small set of stable, efficient, and functionally diverse multipoint active-site mutants that are suitable for low-throughput experimental testing. Our starting point for the FuncLib design was the crystal structure of the most active Kemp eliminase, GNCA4-WT, characterized in our previous work^35^ (*k*_cat_/*K*_M_ of 3047 ± 283 M^–1^ s^–1^ at pH 7 for the protein with a His-tag) (PDB ID: ; 5FQK 35). This structure was provided as a starting point to the FuncLib server, which is available at ; http://FuncLib.weizmann.ac.il. We selected 11 active site positions to diversify, comprising residues in close proximity to the substrate (Fig. 1). The resulting sequence space is shown in Table S1.† The diversification was performed using the default FuncLib parameters, and the transition state analog 5(6)-nitrobenzotriazole present in the crystal structure was retained as a proxy for the substrate 5-nitrobenzisoxazole. This yielded 3000 variants, ordered by the Rosetta scoring energy^82^ (see the Table S5 and the ESI†).

One obvious feature in the FuncLib results is the frequent prediction among the highly scored variants of a phenylalanine residue at position 260 (*vs.* the Leu residue present in the background “WT” protein, denoted here as GNCA4-WT). This is interesting, because, although close to the *de novo* active site, position 260 belongs to a β-strand and its side chain is actually opposite the active site. Therefore, as a first step to explore the FuncLib predictions we assessed the effect of a single L260F mutation on Kemp elimination catalysis. We observe that this L260F mutation by itself is able to enhance both the catalytic efficiency and turnover number by about 2-fold (data not shown). While this is only a moderate increase in activity, it is already comparable to those for the single improved variant obtained from the screening of a non-focused, random mutation library (Table 1).

### Detailed experimental assessment of the FuncLib predictions

For a more detailed assessment, we prepared and determined both the stability and the Kemp eliminase activity of the 20 twenty top FuncLib predictions. The amino acid substitutions included in these variants are shown in Table S6.†

As mentioned before, FuncLib combines phylogenetic analysis and Rosetta calculations to suggest multiple mutations that generate stabilizing interacting networks at the active site. Indeed, the denaturation temperatures of the top 20 variants, as determined by differential scanning calorimetry demonstrate that all enzymes are stable, and two variants even appear to be somewhat more stable than the background (Table 3). This confirms that, despite the substantial number of mutations introduced, the FuncLib predictions avoid substantial protein destabilization. This should be compared with the top ten variants derived from the random library screening (Table 1) which, in some cases, display substantially diminished denaturation temperatures.

**Table 3:** Catalytic parameters for the background and FuncLib variants of the GNCA4/W229F-F290W β-lactamase at pH 7 in the presence of 5% acetonitrile and denaturation temperatures at pH 7 for the same proteins^*a*^

Variant	*k*_cat_ (s^–1^)	*K*_M_ (mM)	*k*_cat_/*K*_M_ (M^–1^ s^–1^)	*T*_M_ (°C)
GNCA4-WT	5.1 ± 0.8	3.7 ± 0.8	1360 ± 101	78.0
GNCA4-1	0.22 ± 0.03	2.2 ± 0.6	102 ± 12	79.1
GNCA4-2	28.9 ± 15	8.12 ± 5	3519 ± 401	78.4
GNCA4-3	4.5 ± 1.6	3.3 ± 1.7	1348 ± 238	79.1
GNCA4-4	0.12 ± 0.14	14 ± 18	8.7 ± 1.2	78.0
GNCA4-5	2.8 ± 0.2	2.3 ± 0.3	1214 ± 11	77.5
GNCA4-6	23 ± 18	24 ± 20	944 ± 54	78.8
GNCA4-7	0.54 ± 0.06	2.8 ± 0.5	190 ± 12	77.7
GNCA4-8	8.2 ± 1.2	2.8 ± 0.7	2856 ± 247	77.6
GNCA4-9	0.17 ± 0.12	5.3 ± 5	31.7 ± 7.3	76.8
GNCA4-10	0.7 ± 0.23	4.8 ± 2	190 ± 22	79.6
GNCA4-11	2.7 ± 0.35	1.8 ± 0.4	1403 ± 153	76.4
GNCA4-12	28 ± 12	6.8 ± 3.7	4127 ± 460	76.0
GNCA4-13	0.4 ± 0.07	2.9 ± 0.7	132 ± 12	75.1
GNCA4-14	1.06 ± 0.07	1.9 ± 0.2	560 ± 32.5	79.6
GNCA4-15	3.1 ± 1.8	9.1 ± 6.3	339 ± 38	77.1
GNCA4-16	1.8 ± 0.07	3.4 ± 1.9	532 ± 96	81.2
GNCA4-17	0.06 ± 0.01	4.4 ± 1.5	15 ± 1.4	77.1
GNCA4-18	4.3 ± 0.4	8.2 ± 0.8	524 ± 9.3	80.9
GNCA4-19	7.1 ± 1.5	2.9 ± 0.9	2366 ± 271	77.9
GNCA4-20	0.3 ± 0.02	1.2 ± 0.1	232 ± 16	83.9

^*a*^Catalytic parameters were determined at pH 7 in the presence of 5% acetonitrile and the His-tag, from fits of the Michaelis–Menten equation to the experimental profiles of rate *vs.* substrate concentration. The use of 5% acetonitrile extends the experimentally available substrate concentration range, but has a slightly detrimental effect on activity (see Fig. 4). This explains the difference between the value given in this table for the “wild type” protein and that given in Table 1. Michaelis plots for variants GNCA4-4 and GNCA4-6 were almost linear, even with the extended substrate concentration range allowed by the addition of 5% acetonitrile. This explains the large uncertainty associated to the determination of *k*_cat_ and *K*_m_ for these variants, specifically. Note that the number following “GNCA” in the variant column corresponds to the ranking of the FuncLib prediction, based on the Rosetta score, as provided in the ESI and in Table S5. The GNCA4-WT baseline variant is referred to here as the “wild-type” (GNCA4-WT). Denaturation parameters were determined at pH 7 by differential scanning calorimetry. For a list of mutations for each variant, see the ESI. All kinetic measurements were performed at 25 °C.

**Fig. 3:**
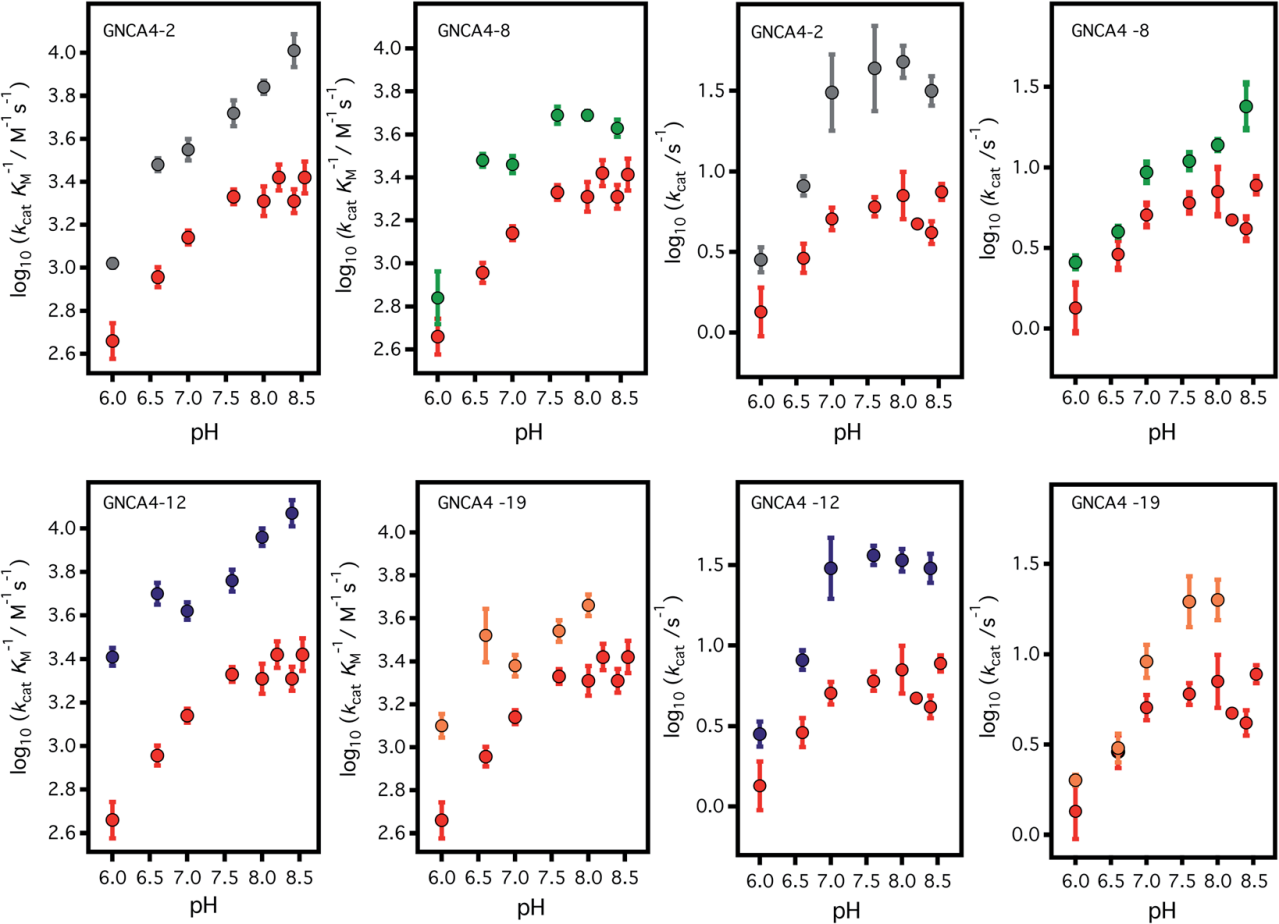
Profiles of (left) catalytic efficiency and (right) turnover number for the 4 best FuncLib variants. In all cases, the profiles are compared with that of the background GNCA4-WT (red data points). All data were obtained in the presence of 5% acetonitrile to increase the substrate concentration range, and to allow for a more accurate determination of the catalytic parameters (*k*_cat_ in particular). Acetonitrile, however, has a slightly detrimental effect on activity (Fig. 4) and, therefore, the values given here for the “wild type” protein are somewhat lower than those previously reported in ref. 35. Agreement is observed, however, upon extrapolation to 0% acetonitrile (Fig. 4).

**Fig. 4:**
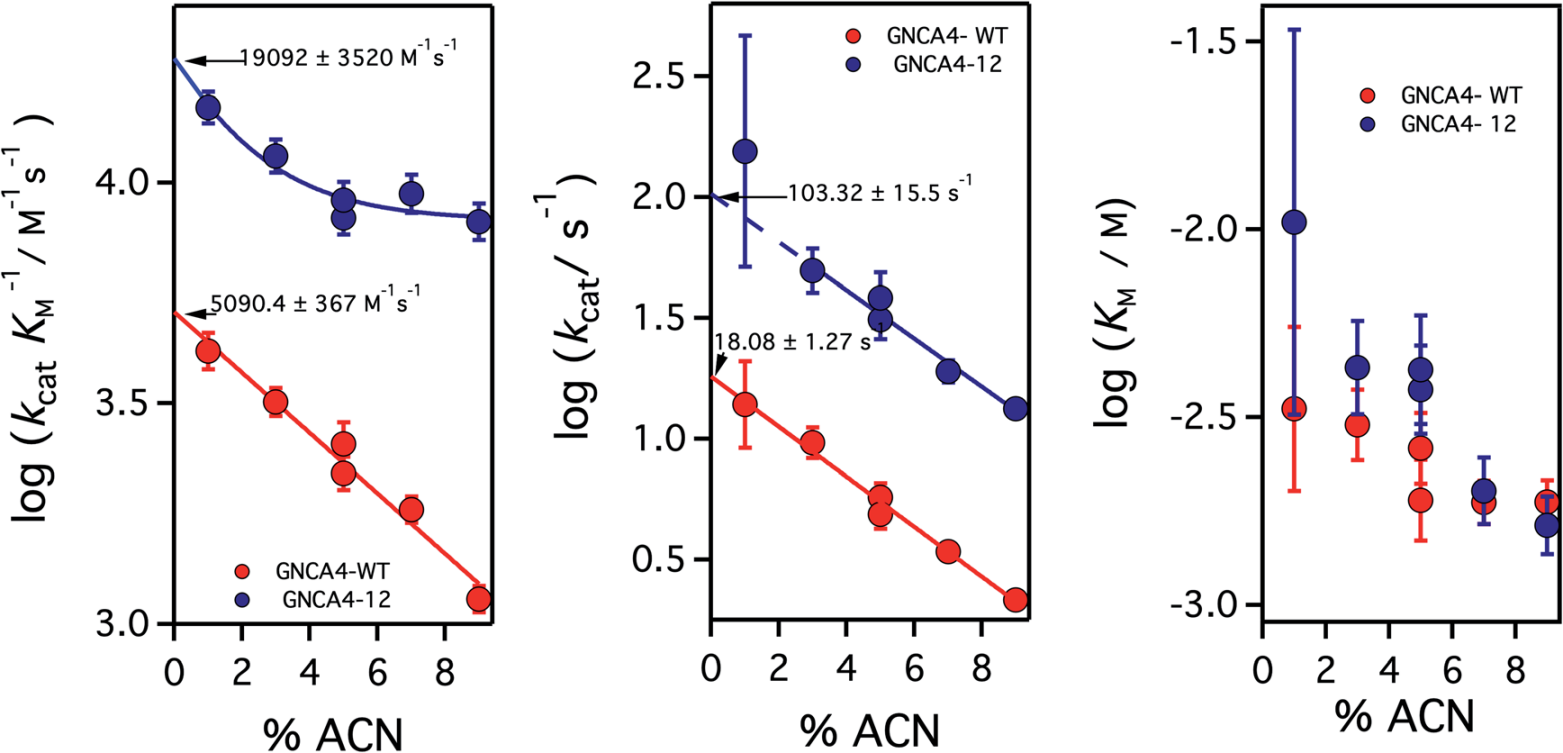
Catalytic parameters for the activity of the background GNCA4-WT protein (red) and the GNCA4-12 variant from the FuncLib prediction, measured at pH 8 and at different acetonitrile (ACN) concentrations. The values were derived from the fitting of the Michaelis–Menten equation to profiles of rate *vs.* substrate concentration. Values of the catalytic parameters in the absence of acetonitrile are obtained through a short extrapolation, as shown. The values extrapolated for the “wild type” protein (red data point) are in good agreement with those reported in ref. 35 at basic pH.

To assess the catalysis levels of the top 20 predicted FuncLib variants, we measured the kinetic activity of several of the predicted sequences at different substrate concentrations and at pH 7 and pH 8.4 (Fig. 2). The catalytic parameters for Kemp elimination catalyzed by the top 20 predicted variants span about two orders of magnitude. This wide range should not be surprising, because FuncLib is not intrinsically intended for predicting catalytically favorable mutations, but rather only to sharply focus the search to regions of the sequence space that encode stable proteins. Still, 4 out of the 20 variants tested display substantially enhanced Kemp eliminase activity with respect to the background variant, both at pH 7 and pH 8.4. The accurate determination of catalytic parameters (in particular the turnover number, *k*_cat_) from the fitting of the Michaelis–Menten equation to the experimental profiles shown in Fig. 2 is impaired in many cases by the available substrate concentration range, which is in turn limited by substrate solubility. Therefore, we additionally determined rate *vs.* substrate concentration profiles in the presence of 5% acetonitrile, which increases substrate solubility by about 3-fold. This allows for an extended substrate concentration range, but at the slight expense of catalytic efficiency. Such studies in the presence of 5% acetonitrile were performed at pH 7 for all the 20 top variants of the FuncLib ranking (Table 3) and, as a function of pH for the 4 best variants. The corresponding profiles of catalytic efficiency and turnover *vs.* pH are compared with those for our background protein, GNCA4-WT in Fig. 3 and 4. These data confirm an enhancement of catalysis over background of up to about one order of magnitude, in particular in the *k*_cat_ value.

It is to be noted, nevertheless that while the addition of 5% acetonitrile has the crucial advantage of increasing the solubility of the substrate for the Kemp elimination reaction, thus expanding the experimental concentration range and allowing for more accurate determination of catalytic parameters, the presence of such a small amount of acetonitrile has a small detrimental effect on catalysis (a decrease of about 2-fold), likely in part through a general solvent effect. Therefore, in order to provide an assessment of the achieved levels of catalytic activity that are not perturbed by cosolvent effects, we performed experiments for the GNCA4-12 variant at pH 8 and several different concentrations of acetonitrile, and we extrapolated the kinetic parameters to zero solvent concentration, as shown in Fig. 4.

Increasing acetonitrile concentrations somewhat depresses the catalytic activity. Two factors may contribute to this. First, since acetonitrile increases substrate solubility, it is also stabilizing the free (non-bound) substrate and thus potentially increasing some of the relevant kinetic free energy barriers. In addition, the interaction of acetonitrile molecules with the protein may directly modify such barriers, through small alterations in the structure or dynamics. This second effect is specific, and may depend on the molecular features of this variant, thus leading to the different extrapolation behaviours. The conjunction of these two factors could perhaps be behind the somewhat complex dependency seen for the catalytic efficiency of the GNCA-12 variant (left panel, Fig. 4). In any case, these speculative interpretations do not affect the main point of Fig. 4, namely that the extrapolations to zero acetonitrile concentration are rather short (even for *k*_cat_) and, therefore, there is little doubt about the reliability of the extrapolated values. The short extrapolation leads to a catalytic efficiency and a turnover number of about 2 × 10^4^ M^–1^ s^–1^ and 10^2^ s^–1^. These values are well within the ranges of catalytic parameters for modern natural enzymes and, in particular, the value 10^2^ s^–1^ for *k*_cat_ is about one order of magnitude higher than the median value of the *k*_cat_ distribution for modern enzymes.^19^

**Fig. 5:**
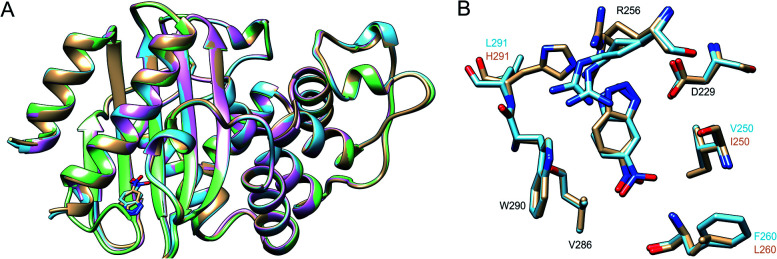
(A) Superposition of the 3D crystal structures of the background GNCA4-WT (tan, PDB ID: 5FQK 35) and the three FuncLib variants whose structure we have determined in this work, specifically the GNCA4-2 (light blue, PDB ID: ; 6TY6), GNCA4-12 (pink, PDB ID: ; 6TXD) and GNCA4-19 (green, PDB ID: ; 6TWW) variants. Highlighted here is also the position of the transition state analogue in the GNCA4-WT and GNCA4-2 variants. (B) A close-up of the *de novo* active site in these enzymes, superimposing the active sites of the background enzyme (tan) and the GNCA4-2 variant predicted from FuncLib (light blue, Table 3), with a transition state analogue bound in the active site. Note that we have changed the orientation of the active site compared to panel (A), to better highlight the changes in key active site side chains.

Finally, we have used X-ray crystallography to determine the 3D-structures of the catalytically optimized GNCA4-2, GNCA4-12 and GNCA4-19 variants, the first of which has a transition state analogue bound at the *de novo* active site. These particular structures were chosen as they are all highly active variants, in terms of the measured rates within the available substrate concentration range (Fig. 2), with improved catalytic parameters over GNCA4-WT (Table 3 and Fig. 3). The protein backbones of these new structures are essentially superimposable with that of the background GNCA4-WT variant (Fig. 5A) and, therefore, the observed enhancement of catalysis is likely linked to small rearrangements in the *de novo* active site (Fig. 5B).

### Empirical valence bond calculations on the FuncLib predictions

The enhancements in catalytic activity reported above have been obtained by following a procedure that did not explicitly take the structure or stabilization of the transition state into account. That is, we simply focused our screening to regions of the sequence space that are meaningful (positions near and at the active site) and also safe to mutate, in the sense that the predicted multiple-mutation variants are not stability-impaired and their folding is not compromised. We were then interested in exploring the extent to which computational calculations on the catalytic step itself could be used to further focus and guide the screening. To this end, we have used the empirical valence bond (EVB) approach^54^ to probe the catalytic activity of the FuncLib predictions, as this approach has been extensively used to successfully study enzyme catalysis in general,^56^ and Kemp elimination in particular.^48^,^57^–^59^ In particular, this allows us to build on our recent work,^59^ in which used the EVB approach to study the evolution of multiple active site configurations in the *de novo* designed Kemp eliminase, KE07.^15^ In the present work, we follow the protocol presented in ref. 59, as described in brief in the Materials and methods.

**Fig. 6:**
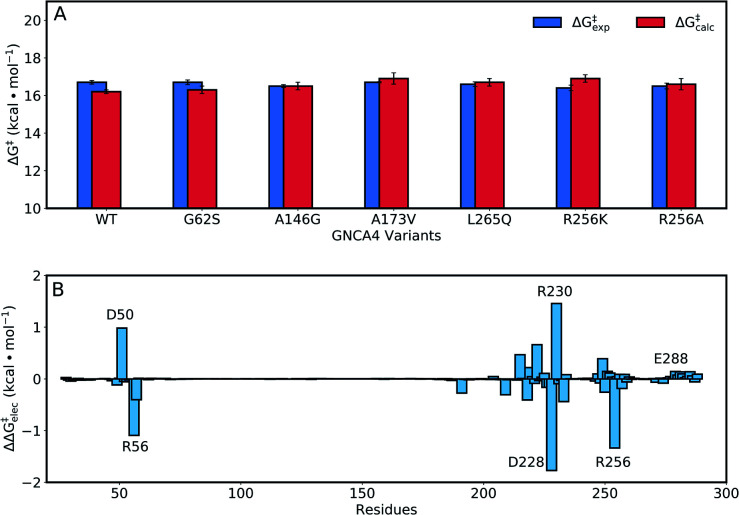
(A) A comparison of calculated (Δ*G*‡calc) and experimental (Δ*G*‡exp) activation free energies for the Kemp elimination of 5-nitrobenzisoxazole by the GNCA4-WT β-lactamase, and a series of its active site mutants (see also Table 1). (B) The electrostatic contributions of individual residues to the calculated activation free energies (ΔΔ*G*‡elec) for the Kemp elimination of 5-nitrobenzisoxazole by the GNCA4-WT β-lactamase (treated as the baseline ‘wild-type’ enzyme in this work). All values were obtained by applying the linear response approximation (LRA)^83^,^84^ to the calculated EVB trajectories, as in our previous works,^85^–^87^ and scaled assuming a dielectric constant of 4 for the highly hydrophobic environment of the *de novo* active site of this β-lactamase (Fig. 1). For the correlation between calculated and experimental values, see Fig. S6.†

As our starting point, we benchmarked our empirical valence bond (EVB) model by performing simulations of our baseline enzyme, GNCA4-WT, as well as six active site mutants: G62S, A146G, A173V, R256A, R256K, L265Q, described in the section Attempting to increase *de novo* enzyme activity through random library screening. As can be seen from Table 2, the effect of these mutations on the catalytic activity is minimal, with a mere 3.3-fold difference in *k*_cat_/*K*_M_ (M^–1^ s^–1^) between the most and least active variants, an effect which is mainly caused by differences in *K*_M_. The *k*_cat_ values are very similar, resulting in activation free energies that are within 0.3 kcal mol^–1^ of each other across the series. Following from this, our EVB simulations were able to reproduce the experimental activation free energy for both the GNCA β-lactamase W290D-F290W to within 0.5 kcal mol^–1^ (Fig. 6A and Table 2).

**Fig. 7:**
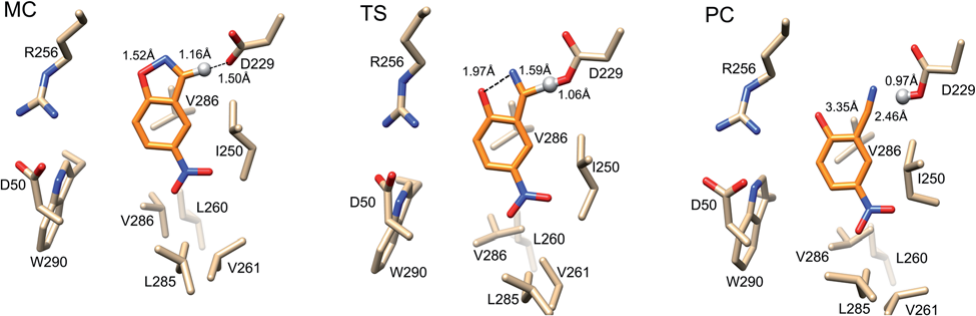
Representative structures of the GNCA4-WT β-lactamase at the Michaelis complex (MC), transition state (TS), and product complex (PC) for the Kemp elimination reaction catalysed by this enzyme, extracted from EVB trajectories of this reaction. Structures were selected based on clustering analysis using the method of Daura *et al.*^88^ as implemented in GROMACS 2016.4.^89^,^90^ The clustering was performed at the MC, TS and PC independently, in order to obtain representative structures for each reacting state. Highlighted here are the donor–hydrogen, acceptor–hydrogen and oxygen–nitrogen distances that are changing during the reaction, and the proton being transferred is shown as a sphere for clarity. Distances are shown as average distances over the entire simulation trajectory (for the corresponding distances for other variants see Tables S7 and S8†).

Representative structures from our simulations of the GNCA4-WT β-lactamase are shown in Fig. 7, with average donor–acceptor distances from our simulations highlighted. The corresponding donor–acceptor distances and donor–hydrogen–acceptor angles for all variants shown in Table 2 can be found in Table S7.† Finally, the electrostatic contributions of individual residues to the calculated activation free energies can be found in Fig. 6B. These contributions were calculated by applying the linear response approximation (LRA)^83^,^84^ to our calculated EVB trajectories, as in our previous work (*e.g.*ref. 85–87). From this data, it can be seen that the individual contributions of most residues to the calculated activation free energies is small (<2 kcal mol^–1^), in line with the fact that the transition state is very similar in structure and in charge distribution to the Michaelis complex.

Having established that our EVB calculations can reliably reproduce the activation free energies of known enzyme variants, we then turned our attention to the top 20 ranked variants from diversification of 11 active site residues (Fig. 1, ESI†), obtained using FuncLib^28^ as described in the Materials and methods. Note that the first variant in the ESI,† with serial number ‘0101010101010101010101’, corresponds to the wild-type enzyme. For simplicity, these variants will be henceforth labelled 1 to 20, starting with the first mutated system, and following the FuncLib ranking.

**Fig. 8:**
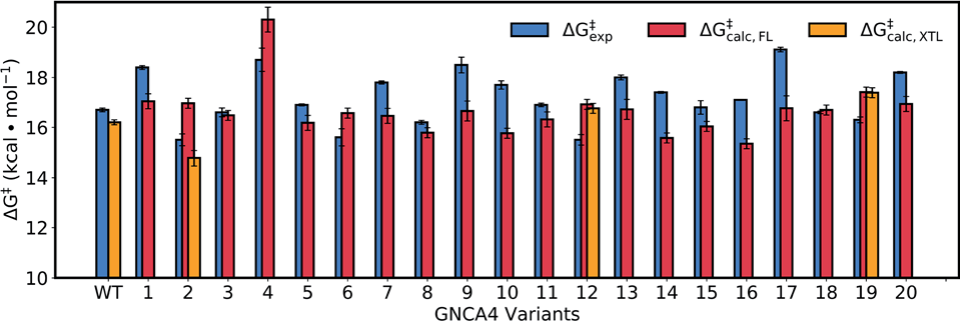
Calculated activation free energies of the Kemp elimination of 5-nitrobenzisoxazole by the GNCA4-WT β-lactamase and the top 20 best scoring variants predicted by FuncLib^28^ (labelled 1 through 20). Shown here are the experimental activation free energies (Δ*G*‡exp) derived from *k*_cat_ based on data presented in Table 3, as well as the corresponding calculated activation free energies based on either structures predicted from FuncLib (Δ*G*‡calc,FL) or, where available, directly from crystal structures (Δ*G*‡calc,XTL). All energies are presented in kcal mol^–1^, and the calculated activation free energies are averages and standard error of the mean over 30 individual EVB trajectories per system, as described in the Materials and methods. The raw data for this figure can be found in Table S5,† and the correlations between experimental and calculated data can be found in Fig. S7.†

Fig. 8 and Table S5† show an overview of the calculated activation free energies for the top 20 FuncLib variants. From this data, it can be seen that in the majority of variants, we obtain very little differences in activation free energy (similar to the prior results shown in Table 1), with at most 1 kcal mol^–1^ improvement compared to GNCA4-WT. The only exception to this is a variant (GNCA4-4) with a high activation free energy of 20.3 kcal mol^–1^. This is due to the introduction of an I250M substitution in this variant. Here, the longer side chain of methionine is located between the substrate and the catalytic D229 side chain, introducing steric hindrance in the active site that displaces the substrate from an optimal binding position and increases the D···A distance at the Michaelis complex substantially (see Table S8†). All other calculated values based on FuncLib predicted structures lie in the range of 15.3–17.4 kcal mol^–1^, compared to a calculation activation free energy of 16.2 kcal mol^–1^ for the wild-type enzyme (Table S5†). We note also that, in general, the 5 variants carrying the I250M substitution (GNCA4-4, GNCA-7, GNCA4-9, GNCA4-13 and GNCA4-17) show higher experimental activation free energies, in the range of 17.8–19.1 kcal mol^–1^ (Table S6†), suggesting that this substitution is kinetically unfavourable.

Overall, there is (from a computational perspective) good agreement with the experimental values, with the calculated values falling to within 2 kcal mol^–1^ of experiment, considering that unlike the calculations on the simpler single amino acid substitutions shown in Fig. 6, in the case of the FuncLib variants, we are now making predictions for the effect of multiple simultaneous variants using computationally predicted structures. Our data is also in agreement with other computational studies of Kemp elimination, that report activation free energies within 2 (or sometimes more) kcal mol^–1^ from experiment.^48^,^49^,^91^–^95^ We note that we have attempted to further refine our EVB calculations by exploring other approaches to generate the starting structures, such as predicting mutations using SCWRL^63^ or inserting point mutations manually using the Dunbrack rotamer library,^96^ as implemented in Chimera.^97^ We tried comparing all three approaches using the GNCA4-2, GNCA4-10 and GNCA4-17 variants as model systems, as these variants show some of the greatest deviations from the experimental values (Table S5†). However, the resulting activation free energies were within 0.4 kcal mol^–1^ of the values obtained using the FuncLib structures (Table S5†), with the exception of GNCA4-2/SCWRL which yielded an activation free energy of 16.0 ± 0.3 kcal mol^–1^ in better agreement with the experimental value of 15.5 kcal mol^–1^ (note however that in the case of GNCA4-2, there was a TSA bound in the active site to guide substrate placement). Therefore, we did not pursue these avenues further as we did not observe systematic improvement in our calculated activation free energies by using alternate approaches to generate the starting structures.

**Fig. 9:**
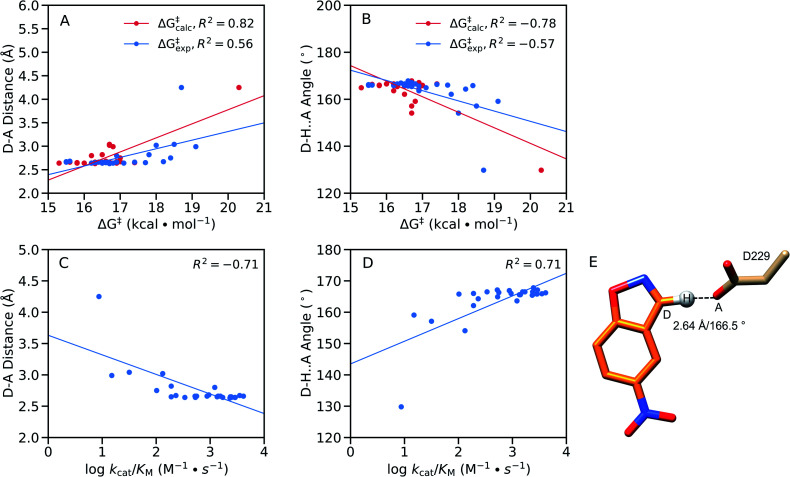
Correlations between the calculated and experimental activation free energies and the (A and C) donor–acceptor (D–A) distances (Å) and (B and D) donor–hydrogen–acceptor (D–H···A) angles (°) in our EVB simulations, calculated based on the data presented in Tables 2, 3, S5, S7 and S8,† using linear regression analysis. Correlations between the geometric parameters and (A and B) calculated activation free energies or (C and D) log *k*_cat_/*K*_M_ are shown here for all variants considered in this work, both single-point mutations and FuncLib predictions. (E) Schematic overview of the orientation of the reacting fragments in the wild-type enzyme. The annotated distance and angle are the average values from our EVB simulations of the wild-type enzyme (Tables S7 and S8†).

From a structural perspective, it can be seen from Table S8† that our EVB calculated transition states are very similar for the wild-type and all twenty simulated FuncLib variants, in terms of D–A distance and D–H···A angle. In addition, the electrostatic contributions of different residues are also relatively similar (Fig. S8†), which is unsurprising in light of the fact that, as discussed elsewhere,^48^ the change in charge distribution between Michaelis complex and transition state is very small, making it hard to obtain any significant gains from electrostatic stabilization in this reaction. Where there are larger differences are in the structures of the reacting atoms at the Michaelis complex, where the D–A distance ranges from 2.64–4.25 Å, and the D–H···A angle ranges from 129.8–167.1°, with significant correlation between the calculated activation free energy and the D–H distance and D–H···A angle (Fig. 9A and B). That is, *R*^2^ = 0.84, and –0.81 for the correlation between the calculated activation free energy and the D–H···A angle when taking into account only the wild-type enzyme and the FuncLib variants, and 0.82 and –0.78 for distances and angles, respectively, when including also the single residue substitutions considered in Table 2. In the case of the experimental data, we still have moderate correlation between the calculated and experimental activation free energies, still, *R*^2^ = 0.56, and –0.57 for the correlation between the experimental activation free energies and to the calculated D–H distances and D–H···A angles.

We note that GNCA4-4 appears to be an outlier on this plot, with a D–A distance of >4.0 Å and a D–H···A angle of ∼130°. Removing this variant from the analysis (Fig. S9†) yields poor correlation between the geometric parameters and the calculated activation free energies. However, removing this variant still gives good correlations of *R*^2^ = 0.68, and –0.64 for the correlation between the experimental activation free energies and to the calculated D–H distances and D–H···A angles, respectively. Finally, we obtain good correlations between log *k*_cat_/*K*_M_ and the calculated D–H distances and D–H···A angles, at *R*^2^ = –0.71, and 0.71 respectively, when the GNCA4-4 variant is included in the analysis (Fig. 9), and *R*^2^ = –0.76 and 0.69 when the GNCA4-4 variant is omitted (Fig. S9†).

We note also that unlike in the case of these geometric parameters (Fig. 9), we do not observe significant correlations with other energetic features of the reaction such as the p*K*_a_ of the catalytic base (predicted using PROPKA 3.1 (ref. 66)) or the reorganization energies. Therefore, it is likely that a significant component of the calculated changes in activity observed upon introduction of the amino acid substitutions predicted by FuncLib is better geometric preorganization of the active site for efficient proton abstraction from the substrate, as was also observed in the case of the crystal structure of the directed-evolution optimized Kemp eliminase HG3.17, compared to the computationally designed HG3.^38^

Finally, one additional feature that can be reducing the quality of our predictions is the fact that the FuncLib variants involve the introduction of up to nine mutations into each structure (of the eleven positions that were selected for randomization, see the ESI†), which is likely to compromise the quality of the FuncLib generated protein structures. To assess this, we also performed EVB simulations on the variants for which crystal structures were available: GNCA-2, GNCA-12 and GNCA-19. For these variants, the calculated values fall to within 1.3 kcal mol^–1^ of the experimental values, and can deviate by up to 2.2 kcal mol^–1^ from the values calculated from the FuncLib predicted structures. In the case of GNCA-2, which has the largest deviation between the calculated activation free energies using the crystal and FuncLib structures (ΔΔ*G*‡calc = 2.2 kcal mol^–1^ with the crystal structure giving better agreement with experiment), we observed subtle structural differences the crystal and FuncLib structures (Fig. S10†). Specifically, we observe different rotamers of the R256 and L291 side chains, as well as also subtle displacements of both the side chain of the catalytic base D229 (which is further from the substrate in the FuncLib predicted structure). The shift in the position of the catalytic base D229 in particular likely plays a significant role in the higher calculated activation free energy for this variant when using the FuncLib predicted structure as a starting point.

Therefore, as can be seen from Table 2 and Fig. 6 and S7(C),† when only a few simultaneous substitutions are involved in generating the computationally predicted structure (as in our prior work^59^,^85^–^87^,^98^,^99^), or where a crystal structure of a variant with multiple amino acid substitutions is available, the EVB approach can reproduce experimental data with high fidelity in a wide range of systems. In addition, considering the potentially large structural perturbations involved, agreement within 2 kcal mol^–1^ of experiment is still respectable, in line with or better than the agreement with experiment obtained in other computational studies of Kemp elimination,^48^,^49^,^91^–^95^ and thus gives EVB great potential as a predictive tool for more complex reactions where the introduction of mutations have a larger energetic impact on the system, and thus better correlation with experiment would be expected as observed for example in ref. 85, 86 and 98–100. Based on this, we believe the EVB simulations can already act as a first step filter over the Rosetta scores predicted by FuncLib, as the latter in this case provided no correlation with experimental activities, despite being able to effectively predict variants with improved activity.

## Concluding remarks

Kemp elimination is a straightforward proton–abstraction reaction that can be performed by a simple molecular machinery consisting, at the bare minimum, of a catalytic base. Accordingly, *de novo* generation of enzyme active sites for Kemp elimination has proved amenable to rational design.^15^,^35^,^38^,^44^,^46^,^94^,^101^ On the other hand, enhancing an already existing Kemp eliminase activity is challenging because of the similarity of the substrate and the transition state for the reaction,^48^ which makes it difficult to find mutations that preferentially stabilize the transition state. Indeed, the best Kemp eliminases reported to date are the results of many rounds of directed evolution starting with rational designs.^38^,^102^

The starting point of the engineering efforts reported here is a Kemp eliminase we previously obtained through minimalist design on a β-lactamase background.^35^ Our design took advantage of the conformational flexibility of an ancestral β-lactamase scaffold to produce both a suitable cavity and a catalytic base within it through a single mutation, while a second mutation enhanced relevant interactions at the *de novo* active site. This led to a *k*_cat_ value of ∼10 s^–1^, which is about the turnover number for an average modern enzyme.^19^ Such a comparatively high starting level of catalysis should further contribute to the (already difficult) task of enhancing Kemp eliminase activity and, indeed, as reported here, screening of 500 clones from a random library led to only one variant with a moderate catalysis improvement. It is remarkable against this backdrop, then, that screening of the 20 top variants from the FuncLib ranking produced 4 variants with improved catalysis (in terms of both *k*_cat_ and *k*_cat_/*K*_M_), of which two showed order-of-magnitude enhancements, bringing *k*_cat_ to the region of 10^2^ s^–1^ (Fig. 4). This value compares well with the best Kemp eliminases reported to date, derived from extensive directed evolution efforts on complex rationally-designed backgrounds. It is in fact somewhat higher than values reported in ref. 102, and it is in the same range as the value (700 s^–1^) reported in ref. 38, in both cases as the outcome of many rounds of directed evolution. Finally, the catalytic efficiency of our best Kemp eliminase (*k*_cat_/*K*_M_ of ∼2 × 10^4^ M^–1^ s^–1^) is only about one order of magnitude below the values obtained from intensive directed evolution, namely 2.3 × 10^5^ M^–1^ s^–1^ by Hilvert and coworkers (HG3.17),^38^ and 5.7 × 10^5^ M^–1^ s^–1^ reported by Tawfik and coworkers using a 5,7-dichloro Kemp substrate,^102^ as well as 1.2 × 10^5^ M^–1^ s^–1^ obtained by computational design using a minimalist approach using the HG3 eliminase as a starting scaffold while incorporating key mutations from the HG3 evolutionary trajectory towards HG3.17 into the design process towards the new Kemp eliminase, HG4.^103^ This is significant because our crystal structures show that, unlike other Kemp eliminases such as HG3 (ref. 38) or KE07,^59^ in the present case it was possible to obtain significant enhancements in catalytic activity without the need for major structural reorganization of the active site.

The striking efficiency of our success with FuncLib-based optimization can be put down to several factors. First, FuncLib is intended to predict stable enzyme variants, a prediction which is in fact confirmed by our thermal denaturation experiments on our Kemp eliminases (Table 3). Therefore, screening effort is not wasted in probing unstable variants that may not fold properly. Secondly, FuncLib can be used to target regions that are expected to be relevant for catalysis (the active site region in this work) and, therefore, screening efforts is not wasted in testing variants with mutations that do not impact catalysis (“neutral” variants). In fact, most of the tested 20 FuncLib predictions show Kemp elimination activities that differ substantially from that of the background used (Table 3 and Fig. 2–4). Thirdly, the fact that FuncLib directly predicts multi-point variants bypasses issues related to epistatic interactions between mutations.

Our results support, overall, that FuncLib predictions may provide an efficient computational methodology to speed up directed evolution by guiding screening to regions of the sequence space that are safe and catalytically-relevant. We have further shown here that the experimental free energy barriers for the optimized eliminases can be reproduced to within ∼2 kcal mol^–1^ by the empirical valence bond calculations. This is impressive in light of the very small changes in activity involved (from a thermodynamic perspective, Table 3) and thus the associated challenges of optimizing Kemp eliminase activity using electrostatics alone.^48^,^58^ We note that other computational studies of Kemp elimination also report activation free energies with deviations within this range or up to several kcal mol^–1^ from experiment.^48^,^49^,^91^–^95^ In addition, whereas we and others have been able to obtain high fidelity with experimental values across a wide range of enzymes and enzyme variants even in the case of far more complex systems than the current Kemp eliminase.^98^–^100^,^104^–^109^ This makes EVB useful as a predictive tool for systems where the changes in energy involved are not as subtle as in the case of Kemp elimination.

In addition, while the FuncLib algorithm focuses on optimizing stability and carries no information about the transition states involved, nevertheless, the best performing FuncLib variants do so due to improved geometric preorganization of the active site through optimizing of the D–H distance and D–H···A angle. This suggests that, in particular for more complex systems where mutations can introduce larger changes in activity, the FuncLib-based stability-guidance could be further refined and focused on the basis of the computational prediction of catalysis, at least in the initial stages of the directed evolution process, during which larger jumps in activity may be possible. This is significant, as FuncLib does not take any information about the substrate or transition state into account in the design process, and therefore while it targets the stability of the overall protein, it does not provide insight into how mutations will affect transition state stabilization.^28^ Clearly, FuncLib can also be used as to generate stable scaffolds that can then be used as a basis for rational design efforts to insert specific physio-chemical properties (such as, for instance, engineering an oxyanion hole) into the active site of the enzyme of interest.

Taken together, the combination of experimental and computational work presented here both showcases the tremendous potential of FuncLib's evolutionary-based stability-screening protocol as a valuable tool in computational enzyme design, as well as the potential of ancestral enzymes as starting scaffolds for artificial enzyme engineering. Here, our crystal structures illustrate that significant gains in activity can be achieved without the need for corresponding significant active site rearrangement. Finally, it is important to note that FuncLib is based on sequence alignment, and thus it would be logical to assume that it would work best for enhancing the reactivity of an enzyme towards its native substrate(s). There remains, however, the question of whether it would also enable the design of function scaffolds that were not designed for those functions. By targeting a non-natural reaction in a *de novo* active site, we demonstrate that FuncLib is a broadly useful tool, that can also be used to design biological catalysts for anthropogenic substrates.

## Author contributions

VAR, ARR, JMSR, and SCLK conceptualized this work. JMSR and SCLK are responsible for data curation. All authors were responsible for investigation and formal analysis of data. JMSR and SCLK were responsible for funding acquisition. VAR, ARR, JMSR, MOM, FSG and JAG were responsible for design of methodology. SCLK and JMSR were responsible for project administration. SCLK, JMSR, JAG and FSG provided resources for this work. SCLK was responsible for software for the computational part of the manuscript. SCLK, JMSR, JAG and FSG were responsible for supervision of this work. VAR, ARR, JMSR and SCRLK were responsible for validation, visualization, writing and review and editing of the manuscript, with input from all authors.

## Conflicts of interest

There are no conflicts to declare.

## Supplementary Material

Supplementary information

Supplementary information
