# Supplementary material for: Enhancing a de novo enzyme activity by computationally-focused ultra-low-throughput screening
Source: Chem Sci. 2020 May 19;11(24):6134–48. doi: 10.1039/d0sc01935f (PMC7407621; doi:10.1039/d0sc01935f)
Supplement: Supplementary file 1 [file SC-011-D0SC01935F-s001.pdf]

Electronic Supplementary Material (ESI) for Chemical Science.
This journal is © The Royal Society of Chemistry 2020

Supporting Information for:

Enhancing a *De Novo* Enzyme Activity by Computationally-Focused, Ultra-Low-Throughput Sequence Screening

Valeria A. Risso,[1] Adrian Romero-Rivera,[2] Luis I. Gutierrez-Rus,[1] Mariano Ortega-Muñoz,[3] Francisco Santoyo-Gonzalez,[3] Jose A. Gavira,[4] Jose M. Sanchez-Ruiz,[*,1] Shina C. L. Kamerlin[*,2]

1. Departamento de Química Física, Facultad de Ciencias, University of Granada, 18071-Granada, Spain
2. Science for Life Laboratory, Department of Chemistry-BMC, Uppsala University, BMC Box 576, S-751 23 Uppsala, Sweden
3. Departamento de Química Organica, Facultad de Ciencias, University of Granada, 18071-Granada, Spain
4. Laboratorio de Estudios Cristalograficos, Instituto Andaluz de Ciencias de la Tierra, CSIC-University of Granada, Avenida de las Palmeras 4, Granada 18100 Armilla, Spain

Corresponding author email addresses: sanchezr@ugr.es and lynn.kamerlin@kemi.uu.se

Table of Contents

Supplementary Figures……..……………………..…………………………..………………………. S4

Figure S1. Root mean square deviations during equilibration of simulations of the GNCA4-WT and of variants with single amino acid substitutions……………………………………………………….....S4

Figure S2. Root mean square deviations during equilibration of simulations of the twenty FuncLib variants..………….……………….. ………………………………………………………………..S5

Figure S3. Root mean square deviations during equilibration of simulations of the crystal structures of the three FuncLib variants……………………………………………...…………..………. S6

Figure S4. Kemp eliminase activity of 522 clones from a random library on the *de novo* GNCA4-WT *β*-lactamase..……………………………………………..……………….…………………S6

Figure S5. Plots of Kemp eliminase activity *vs.* substrate concentration for FuncLib variants………S7

Figure S6. Correlation between calculated and experimental activation free energies for the Kemp elimination of 5-nitrobenzisoxazole by the GNCA4-WT and a series of active site mutants…………...S8

Figure S7. Correlation between calculated energetics and experimental activation free energies for the Kemp elimination of 5-nitrobenzisoxazole by the GNCA4-WT and the top twenty variants predicted from the FuncLib webserver…………………………………………………………………...S9

Figure S8. Electrostatic contributions of individual residues to the calculated activation free energies for the Kemp elimination of 5-nitrobenzisoxazole by the top 20 best scoring GNCA4 variants predicted by FuncLib……..…..…..S10

Figure S9. Correlation between geometric parameters and calculated and experimental activation free energies and experimentally measured kinetics……………………………………………………S11

Figure S10. Comparison of the X-ray and FuncLib predicted structures of the GNCA4-2 variant….S12

Supplementary Tables ……………………………………………………..…………………….... S13

Table S1. Sequence space explored after diversification of 11 active site residues by the FuncLib webserver..…………………………………………………………………………………………S13

Table S2. Ionized residues and protonation patterns of histidine residues during EVB simulations of the β-lactamase catalyzed Kemp elimination of 5-nitrobenzisoxazole............……………………......…...S14

Table S3. Percentage acetonitrile used at different substrate concentrations…………………………S14

Table S4. Data collection and refinement statistics of the 3D structural models..………………...…. S15

Table S5. Rosetta scores and calculated and experimental activation free energies for the GNCA4-WT β-lactamase, as well as the top twenty variants predicted from FuncLib..…………………...…….. S16

Table S6. Amino acid substitutions introduced in the twenty top-ranked FuncLib variants…………..S17

Table S7. Average donor-acceptor (D-A) distances and donor-hydrogen-acceptor (D-H…A) angles obtained from EVB simulations of different experimentally characterized variants of the GNCA4-WT β-lactamase..…………………..……………………………………………………………….. S18

Table S8. Average donor-acceptor (D-A) distances and donor-hydrogen-acceptor (D-H…A) angles obtained from EVB simulations of the GNCA4-WT β-lactamase, as well as of the top twenty variants predicted from FuncLib. ………………………………………………….……………………. S19

Supplementary References ………………………………………………….……………………S20

Supplementary Figures

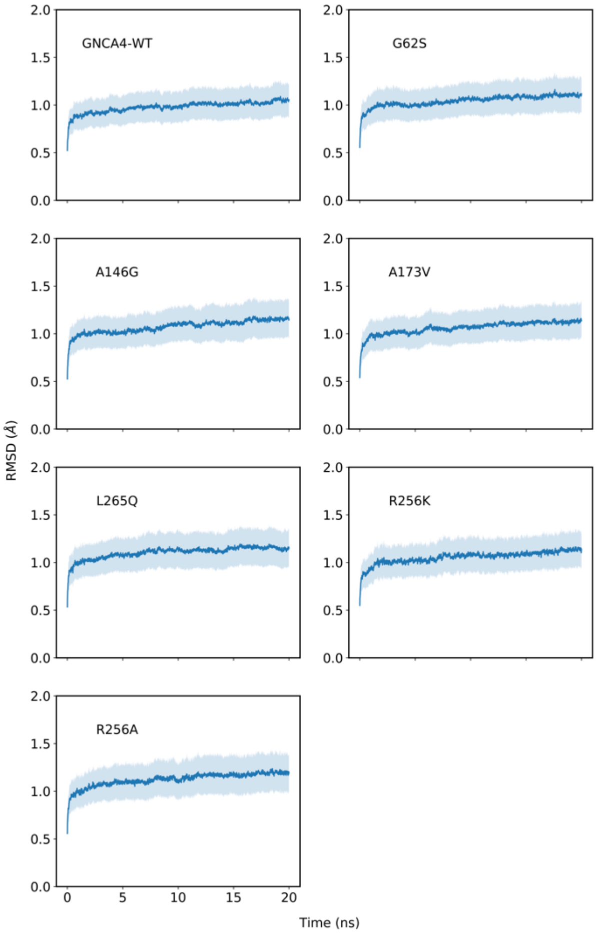

Figure S1. The root mean square deviations (RMSD, Å) of all backbone atoms of the GNCA4-WT and single amino acid substitutions used for the calibration of our EVB model, at the approximate EVB transition state (λ = 0.5) for the Kemp elimination reaction catalyzed by these enzymes. Data was collected every 10 ps from the initial equilibration runs, and is shown as averages and standard deviations over ten individual 20 ns MD simulations per system (*i.e.* 200 ns cumulative simulation time per system). The average RMSD per system is denoted by solid blue lines, and the standard deviations per point over all trajectories are illustrated by the shaded area on each plot.

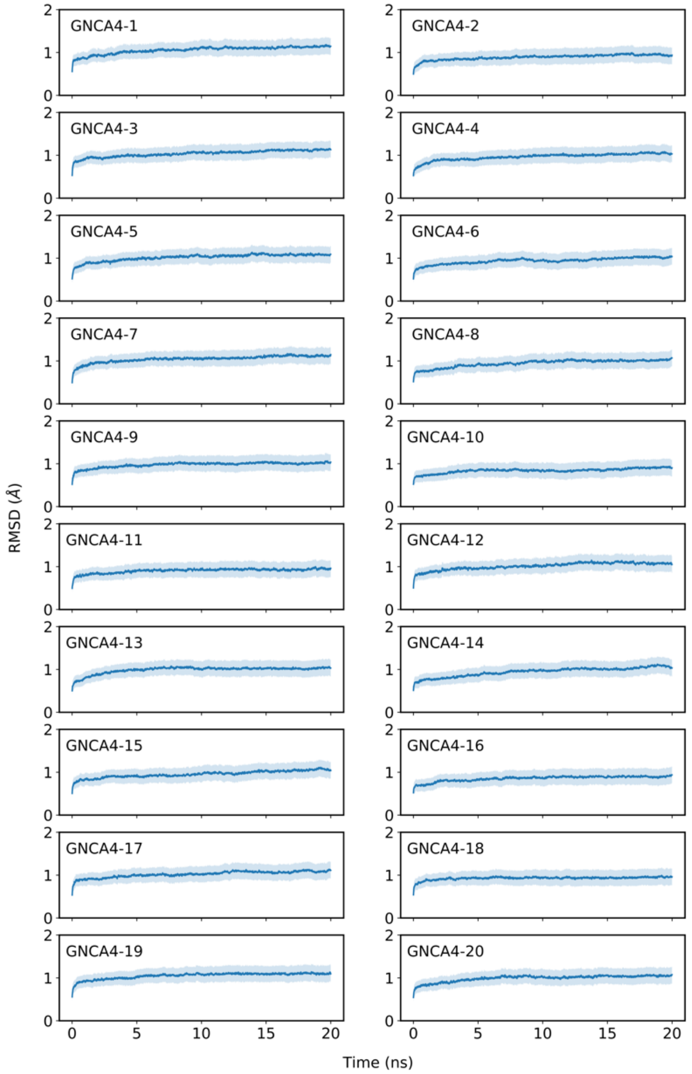

Figure S2. The root mean square deviations (RMSD, Å) of all backbone atoms of the twenty FuncLib variants studied in this work (computationally predicted structures), at the approximate EVB transition state ($\lambda = 0.5$) for the Kemp elimination reaction catalyzed by these enzymes. Data was collected every 10 ps from the initial equilibration runs, and is shown as averages and standard deviations over ten individual 20 ns MD simulations per system (*i.e.* 200 ns cumulative simulation time per system). The average RMSD per system is denoted by solid blue lines, and the standard deviations per point over all trajectories are illustrated by the shaded area on each plot.

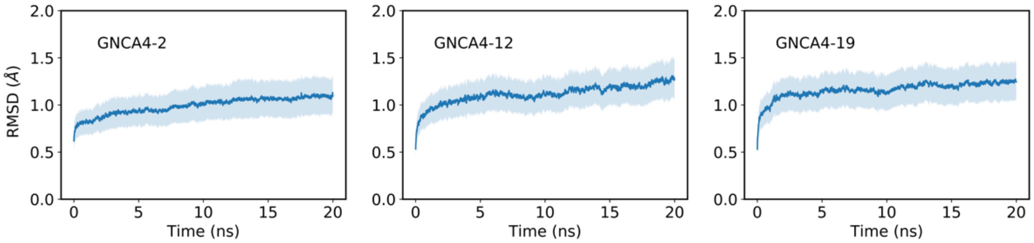

Figure S3. The root mean square deviations (RMSD, Å) of all backbone atoms of the three FuncLib variants studied in this work for which crystal structures are available, at the approximate EVB transition state (λ = 0.5) for the Kemp elimination reaction catalyzed by these enzymes. Data was collected every 10 ps from the initial equilibration runs, and is shown as averages and standard deviations over ten individual 20 ns MD simulations per system (*i.e.* 200 ns cumulative simulation time per system). The average RMSD per system is denoted by solid blue lines, and the standard deviations per point over all trajectories are illustrated by the shaded area on each plot.

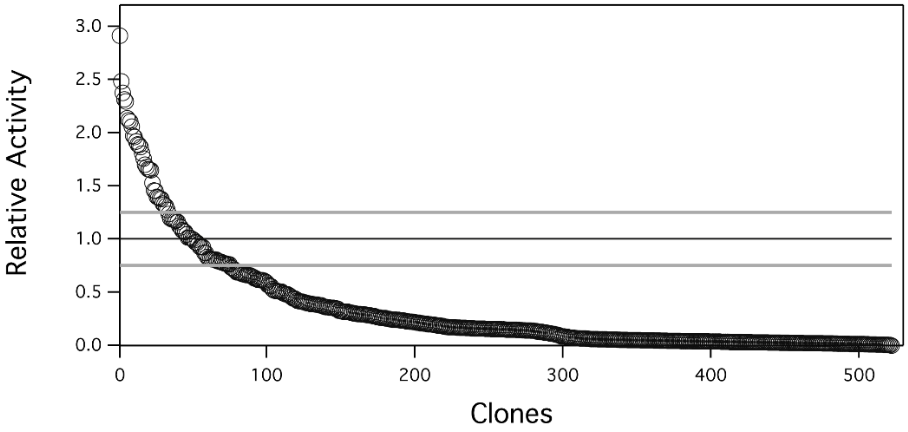

Figure S4. The Kemp eliminase activity of 522 clones from a random library prepared on the *de novo* GNCA4-WT *β*-lactamase (mutational load 3-5 mutations). The activity of these clones is shown relative to the activity of the background enzyme (shown as a black horizonal line). The grey horizontal lines represent the standard deviation interval for the background variant derived from measurements performed on 52 clones.

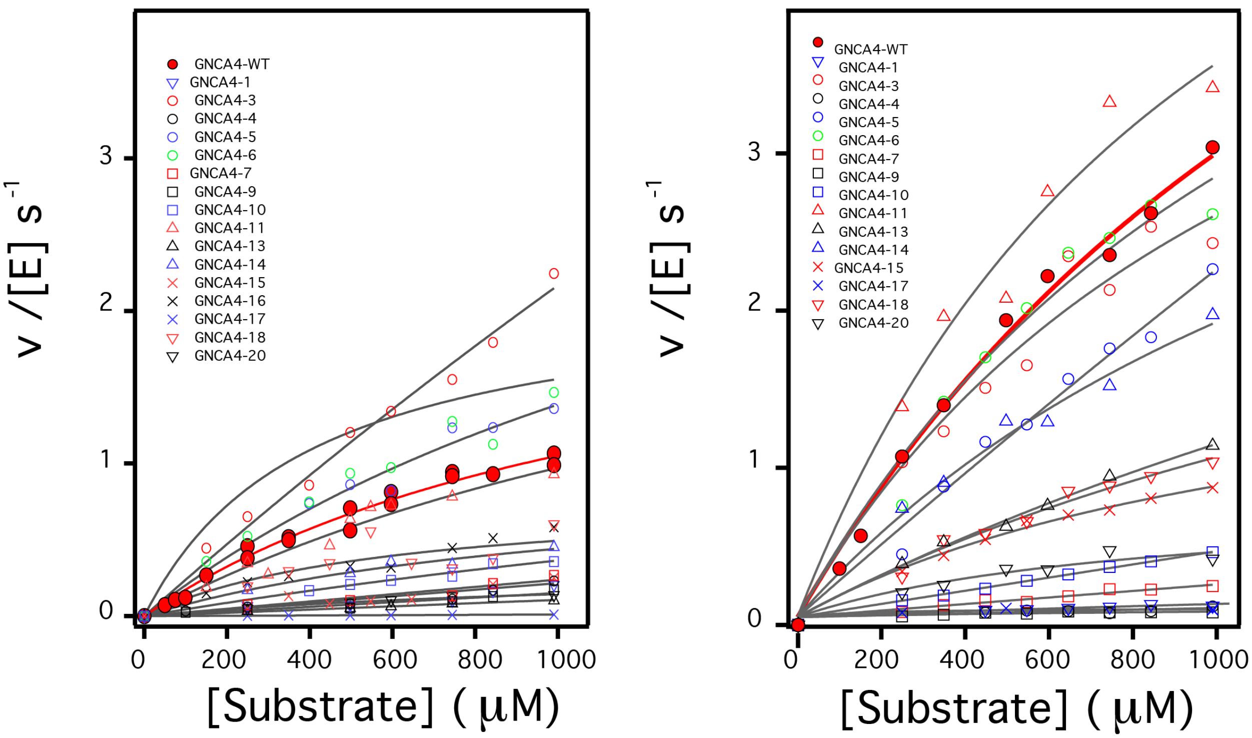

Figure S5. Plots of Kemp eliminase activity *vs*. substrate concentration at (**left**) pH 7 and (**right**) pH 8.4. Activities for the background protein (GNCA4-WT), as well as the 4 variants that display substantially enhanced catalysis at both pH values are found in **Figure 2**. Shown here are the activities of the GNCA4-WT and the remaining 16 variants from the top 20 variants from the FuncLib prediction (**Table S5**). The lines are the best fits of the Michaelis-Menten equation.

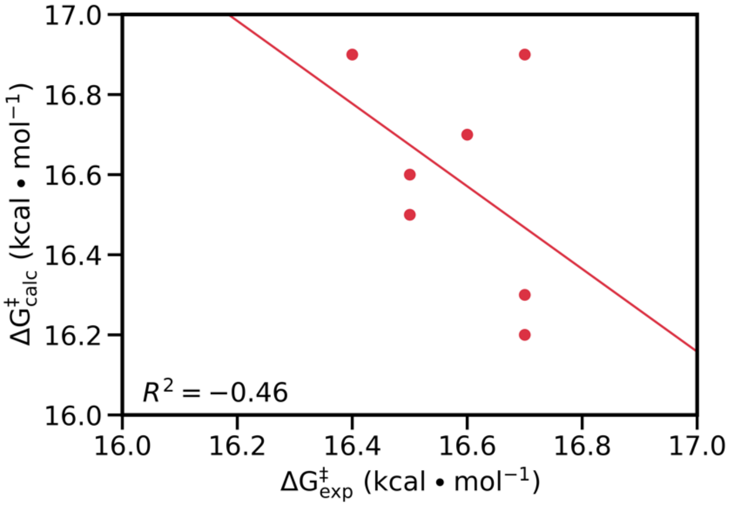

Figure S6. Correlation between calculated and experimental activation free energies for the Kemp elimination of 5-nitrobenzisoxazole by the GNCA4-WT and a series of active site mutants, calculated using linear regression analysis. The raw data for this figure is shown in **Table 2**. The correlation between the calculated and experimental activation free energies, calculated using linear regression analysis, is -0.46. Note that the differences in energies for each system are so small, that even very small thermodynamic differences can lead to weaker correlation with the experimental values.

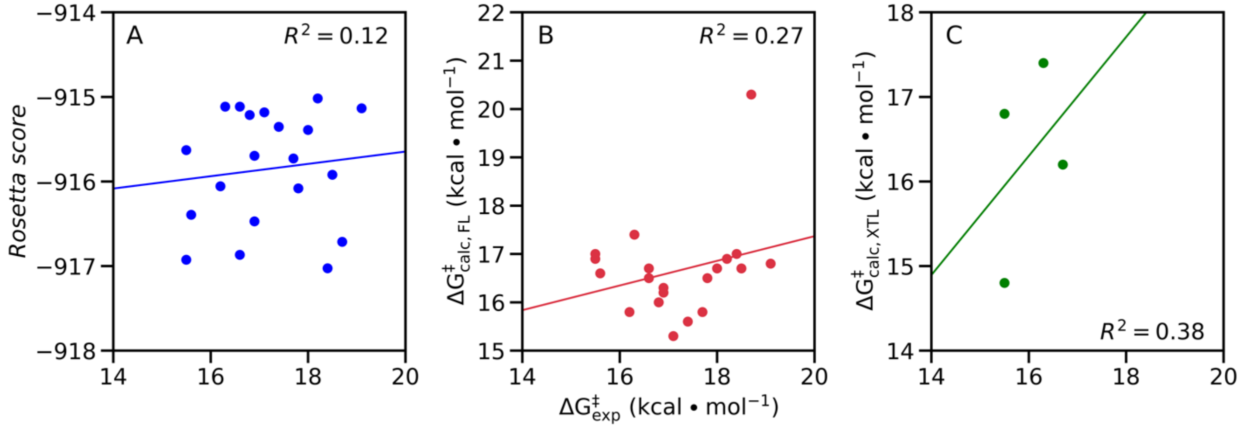

Figure S7. Correlation between (**A**) the Rosetta score from FuncLib and the experimental activation free energies ($\Delta G^{\ddagger}_{exp}$), (**B**) the activation free energies calculated using the structure predictions from FuncLib ($\Delta G^{\ddagger}_{calc,FL}$) and $\Delta G^{\ddagger}_{exp}$, and (**C**) the activation free energies calculated directly from crystal structures, where available, and $\Delta G^{\ddagger}_{exp}$. The raw data for this figure can be found in **Table S5**. Note that, for consistency, we did not include the GNCA4-WT in the correlation calculations for panels (**A**) and (**B**), as this is not a FuncLib predicted variant. As can be seen, in terms of the correlation between the calculated and experimental values, there is a weak correlation between calculated and experimental activation free energies (R^2 = 0.27, calculated using linear regression analysis, note that we have removed the GNCA4-WT from this correlation as this is not a FuncLib predicted structure). This is, however, due to the fact that the energy differences involved are, from a computational perspective, so small that even small deviations from the experimental value will lead to weak correlation with experiment. In terms of the comparison between the Rosetta score obtained from FuncLib (**Table S4**) and the experimental activation free energy, we obtain essentially no correlation with experiment (R^2 = 0.12, again omitting the GNCA4-WT for the same reason as above), which likely reflects the fact that the FuncLib ranking does not include any information about the substrate or transition state, and is based exclusively on the stability of the scaffold.[1] Similarly, for the variants where we have crystal structures available (GNCA4-WT, GNCA4-2, GNCA4-12 and GNCA4-19), we obtain similar correlation between calculated and experimental activation free energies (R^2 = 0.38), although this is a correlation over only 4 enzyme variants, and the energy difference between the calculated and experimental values is always within ~1 kcal·mol^{-1} of the experimental value,

indicating again that the weak correlation coefficients in this specific case are mainly due to the very small energy differences involved (which are within the resolution of EVB and other QM/MM methodologies as described in the main text), rather than a problem with the method.

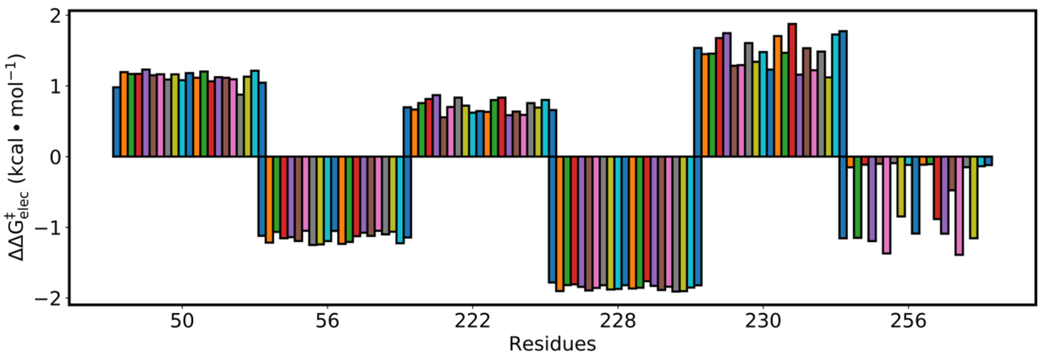

Figure S8. The electrostatic contributions of individual residues to the calculated activation free energies (ΔΔG‡elec) for the Kemp elimination of 5-nitrobenzisoxazole by the top 20 best scoring GNCA4 variants predicted by FuncLib.[1] All values were obtained by applying the linear response approximation (LRA)[2, 3] to the calculated EVB trajectories, as in our previous works,[4-6] and scaled assuming a dielectric constant of 4 for the highly hydrophobic environment of the *de novo* active site of this *β*-lactamase (**Figure 1**). Note that the deviations observed for residue 256 are due to the mutation of this residue (**Table S1**).

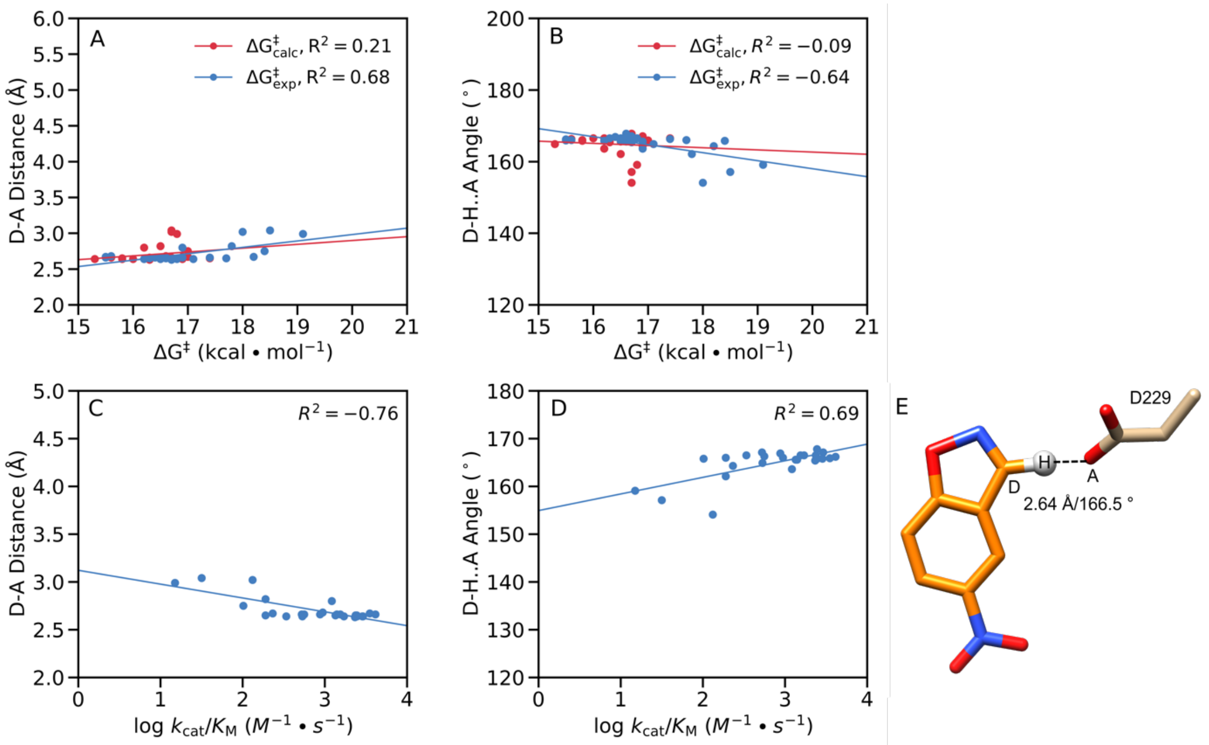

Figure S9. Correlations between the calculated and experimental activation free energies and the (**A**, **C**) donor-acceptor (D-A) distances (Å) and (**B**, **D**) donor-hydrogen-acceptor (D-H…A) angles (°) in our EVB simulations, calculated based on the data presented in **Tables 2, 3, S5, S7** and **S8**, using linear regression analysis. Correlations between the geometric parameters and (**A**, **B**) calculated activation free energies or (**C**, **D**) log k_{cat}/K_M are shown here for all variants considered in this work, both single-point mutations and FuncLib predictions, with the exception of the GNCA4-4 variant, which is an outlier in the data as shown in **Figure 9**. (**E**) Schematic overview of the orientation of the reacting fragments in the wild-type enzyme. The annotated distance and angle are the average values from our EVB simulations of the wild-type enzyme (**Tables S7** and **S8**).

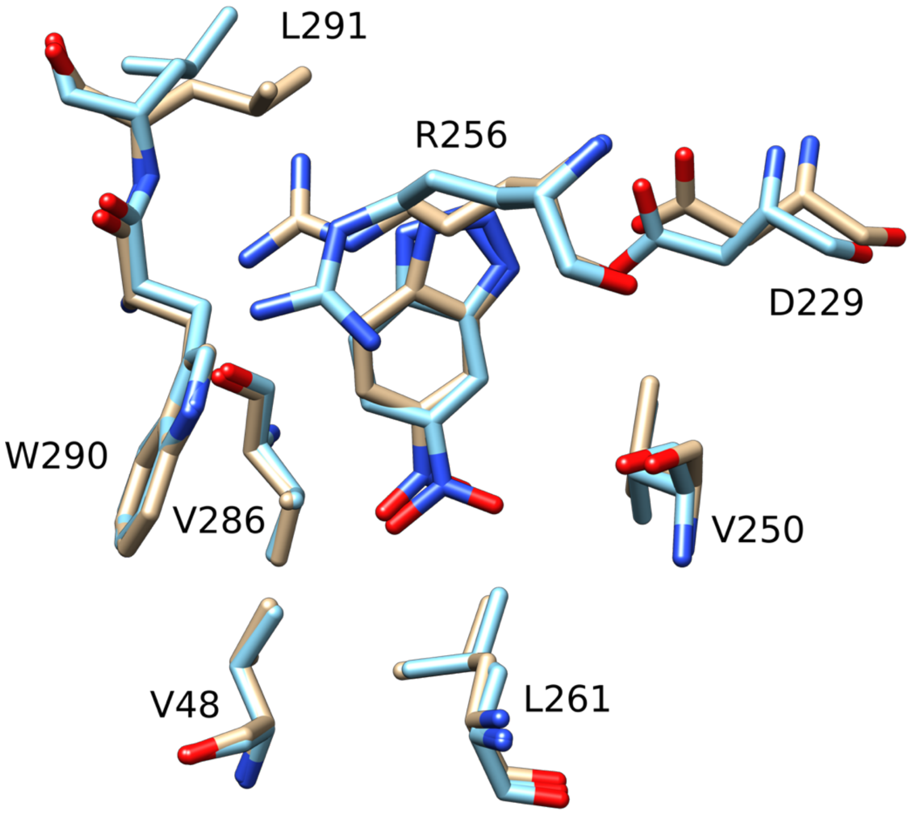

Figure S10. Overlay of the crystal structures of the GNCA4-2 variant obtained *via* (**blue**) X-ray crystallography (PDB ID: 6TY6) and (**tan**) FuncLib prediction.

Supplementary Tables

Table S1. Sequence space explored after diversification of 11 active site residues by the FuncLib webserver.[1]

**Original Residue**	**FuncLib Predictions**
V48	VIL
D50	D
I250	ILMV
R256	RHKQ
L260	LFIMV
V261	VILM
L285	LAVW
V286	VAILM
V287	VAILMST
W290	W
H291	HEFIKLMNQRTV

Table S2. List of ionized residues as well as the protonation patterns of histidine residues in EVB simulations of the *β*-lactamase catalyzed cleavage of 5-nitrobenzisoxazole *via* Kemp elimination.[a]

Residue Type	Residue Number
Asp	50, 209, 218, 228, 229, 233, 246, 273, 276
Glu	281
Arg	55, 56, 191, 204, 220, 222, 230, 256, 284
Lys	215, 219, 234
His-δ	122, 241
His-ε	None

[a] All residues not listed here were kept in their unionized forms during the simulations, as they fell outside the explicit simulation sphere (see the **Methodology** section of the main text). Protonation states and numbering based on residue numbering in PDB ID: 5FQK.[7]

Table S3. Percentage acetonitrile (%ACN) used during kinetic measurements at different substrate concentrations.

%ACN	5-Nitrobenzisoxazole Concentration Range (mM)
1	0-1
3	0-2.2
5	0-2.4
7	0-2.9
9	0-2.9

Table S4. Data collection and refinement statistics of the 3D structural models.[a]

	GNCA4-2	GNCA4-12	GNCA4-19
PDB ID	6TY6	6TXD	6TWW
Wavelength (Å)	0.97926	0.97926	0.97926
Resolution range	47.35 - 1.8 (1.864 - 1.8)	71.74 - 2.0 (2.071 - 2.0)	47.31 - 1.381 (1.431 - 1.381)
Space group	*I*222	*I*222	$P6_522$
Unit cell (Å, °)	78.38 148.35 246.01 90 90 90	77.31 148.23 245.96 90 90 90	78.23 78.2 198.32 90 90 120
Total reflections	678420 (70746)	425809 (42214)	1134923 (70380)
Unique reflections	130796 (13114)	95172 (9368)	74330 (7295)
Multiplicity	5.2 (5.4)	4.5 (4.5)	15.3 (9.6)
Completeness (%)	98.56 (99.39)	98.99 (98.87)	99.98 (100.00)
Mean I/sigma(I)	10.77 (1.20)	11.89 (2.63)	27.24 (2.43)
Wilson B-factor (Å^2)	30.31	28.62	15.91
R-merge	0.08955 (1.443)	0.1031 (0.6784)	0.04978 (0.6767)
CC1/2	0.997 (0.678)	0.993 (0.81)	1 (0.887)
Refinement			
R-work	0.2005 (0.3722)	0.2125 (0.2954)	0.1505 (0.2004)
R-free	0.2280 (0.3997)	0.2373 (0.3297)	0.1659 (0.2132)
CC(work)	0.965 (0.831)	0.953 (0.877)	0.969 (0.930)
CC(free)	0.952 (0.798)	0.951 (0.838)	0.975 (0.919)
Number of atoms	7290	6729	2617
protein	6548	6248	2209
ligands	138	59	36
solvent	604	422	372
Number of chains	3	3	1
RMS(bonds) (Å)	0.019	0.003	0.013
RMS(angles) (°)	1.44	0.60	1.27
Ramachandran favored (%)	98.35	98.48	98.11
Ramachandran outliers (%)	0.00	0.00	0.00
Rotamer outliers (%)	2.38	1.25	0.87
Average B-factor (Å^2)	40.03	38.25	20.22
macromolecules (Å^2)	39.04	37.96	17.35
ligands (Å^2)	56.53	51.32	37.30
solvent (Å^2)	46.90	40.63	35.59
Number of TLS groups	18	21	5

[a] Statistics for the highest-resolution shell are shown in parentheses.

Table S5. Rosetta scores[8] and calculated and experimental activation free energies for the GNCA4-WT *β*-lactamase, as well as the top twenty variants predicted from FuncLib.[a]

Variant	Rosetta Score	$\Delta G^{\ddagger}_{exp}$	$\Delta G^{\ddagger}_{calc,XTL}$	$\Delta G^{\ddagger}_{calc,FL}$	$\Delta\Delta G^{\ddagger}_{exp\to calc,XTL}$	$\Delta\Delta G^{\ddagger}_{exp\to calc,FL}$
GNCA4-WT	-906.616	16.7	16.2 ± 0.1	-	-0.5	-
GNCA4-1	-917.026	18.4	-	17.0 ± 0.3	-	-1.4
GNCA4-2	-916.926	15.5	14.8 ± 0.3	17.0 ± 0.2	-0.7	1.5
GNCA4-3	-916.868	16.6	-	16.5 ± 0.2	-	-0.1
GNCA4-4	-916.714	18.7	-	20.3 ± 0.5	-	1.6
GNCA4-5	-916.472	16.9	-	16.2 ± 0.3	-	-0.7
GNCA4-6	-916.394	15.6	-	16.6 ± 0.2	-	1.0
GNCA4-7	-916.082	17.8	-	16.5 ± 0.3	-	-1.3
GNCA4-8	-916.056	16.2	-	15.8 ± 0.2	-	-0.4
GNCA4-9	-915.920	18.5	-	16.7 ± 0.4	-	-1.8
GNCA4-10	-915.728	17.7	-	15.8 ± 0.2	-	-1.9
GNCA4-11	-915.696	16.9	-	16.3 ± 0.3	-	-0.6
GNCA4-12	-915.629	15.5	16.8 ± 0.2	16.9 ± 0.2	1.3	1.4
GNCA4-13	-915.391	18.0	-	16.7 ± 0.4	-	-1.3
GNCA4-14	-915.354	17.4	-	15.6 ± 0.2	-	-1.8
GNCA4-15	-915.214	16.8	-	16.0 ± 0.2	-	-0.8
GNCA4-16	-915.183	17.1	-	15.3 ± 0.2	-	-1.8
GNCA4-17	-915.135	19.1	-	16.8 ± 0.5	-	-2.3
GNCA4-18	-915.116	16.6	-	16.7 ± 0.2	-	0.1
GNCA4-19	-915.115	16.3	17.4 ± 0.2	17.4 ± 0.2	1.1	1.1
GNCA4-20	-915.018	18.2	-	16.9 ± 0.3	-	-1.3

[a] The GNCA4-WT *β*-lactamase, which is used as the baseline for our study, is referred to in this table as "wild-type" ("GNCA4-WT"). Experimental activation free energies ($\Delta G^{\ddagger}_{exp}$) were derived from k_{cat}, where available, based on kinetic data presented in **Tables 2** and **3** (note that all calculations were performed without a His-tag, and therefore kinetic data from **Table 2** was used for the GNCA4-WT). Calculated activation free energies ($\Delta G^{\ddagger}_{calc,XTL}$ if calculated based on an available crystal structure, and ($\Delta G^{\ddagger}_{calc,FL}$ if calculated based on the structure predicted by FuncLib) are presented as average values and standard error of the mean over thirty independent EVB trajectories per system. The $\Delta\Delta G^{\ddagger}$ values represent the difference between the experimental activation free energy and the calculated activation free energy based on crystal structures or structures obtained from FuncLib, respectively. All energies are presented in kcal·mol^{-1}. '-' indicates 'data not available'. For the full list of FuncLib[1] predictions, see the **Supplementary Data**.

Table S6. Amino acid substitutions introduced in the twenty top-ranked FuncLib[1] variants.

Variant	48	50	250	256	260	261	285	286	287	290	291
GNCA4-WT	V	D	I	R	L	V	L	V	V	W	H
GNCA4-1	V	D	I	H	F	I	L	V	L	W	I
GNCA4-2	V	D	V	R	F	L	L	V	V	W	L
GNCA4-3	V	D	I	H	F	I	L	V	V	W	H
GNCA4-4	V	D	M	R	F	I	L	V	L	W	I
GNCA4-5	V	D	I	H	F	L	L	V	V	W	I
GNCA4-6	V	D	V	R	F	I	L	I	V	W	I
GNCA4-7	V	D	M	H	F	L	L	V	V	W	K
GNCA4-8	V	D	I	R	F	I	L	V	V	W	K
GNCA4-9	V	D	M	H	F	I	L	V	A	W	H
GNCA4-10	V	D	I	R	F	L	L	V	L	W	I
GNCA4-11	V	D	V	H	F	L	L	V	T	W	H
GNCA4-12	V	D	V	H	F	L	L	V	V	W	V
GNCA4-13	V	D	M	R	F	L	L	V	V	W	H
GNCA4-14	V	D	I	R	F	L	L	V	A	W	Q
GNCA4-15	I	D	I	R	F	L	L	I	V	W	I
GNCA4-16	V	D	V	R	V	L	L	V	L	W	N
GNCA4-17	V	D	M	H	V	I	L	V	V	W	I
GNCA4-18	V	D	V	R	I	I	L	I	V	W	L
GNCA4-19	V	D	V	H	F	I	L	I	V	W	H
GNCA4-20	V	D	I	H	I	I	L	V	T	W	N

Table S7. Average donor-acceptor (D-A) distances and donor-hydrogen-acceptor (D-H…A) angles obtained from EVB simulations of different experimentally characterized variants of the GNCA4-WT *β*-lactamase.[a]

**Variants**	**D-A**			**D-H..A**		
	**MC**	**TS**	**PC**	**MC**	**TS**	**PC**
GNCA4-WT	2.64±0.06	2.63±0.07	3.30±0.26	166.5±6.3	167.6±5.9	148.3±19.0
A146G	2.63±0.07	2.63±0.06	3.24±0.20	165.4±6.3	166.2±6.4	155.3±12.1
A173V	2.64±0.08	2.63±0.06	3.24±0.19	165.8±6.7	168.2±5.9	156.1±12.7
G62S	2.64±0.07	2.63±0.06	3.22±0.21	167.1±6.0	167.3±6.5	154.1±12.5
L265Q	2.65±0.08	2.63±0.06	3.25±0.19	167.8±6.9	169.1±6.1	155.0±14.9
R256A	2.66±0.07	2.64±0.06	3.29±0.20	166.9±6.0	167.8±6.4	155.3±10.6
R256K	2.66±0.08	2.65±0.07	3.29±0.22	166.5±6.6	166.3±5.7	151.3±19.2

[a] D-A distances are presented in Å and D-H..A angles are presented in °. Data is shown as average values and standard deviations over 30 independent trajectories. MC, TS and PC denote the Michaelis complex, transition state, and product complex, respectively.

Table S8. Average donor-acceptor (D-A) distances and donor-hydrogen-acceptor (D-H…A) angles obtained from EVB simulations of the GNCA4-WT *β*-lactamase, as well as of the top twenty variants predicted from FuncLib.[a]

Variants	D-A			D-H..A		
	MC	TS	PC	MC	TS	PC
GNCA4-WT	2.64 ±0.06	2.63 ±0.07	3.30 ±0.26	166.5 ±6.3	167.6 ±5.9	148.3 ±19.0
GNCA4-1	2.75 ±0.49	2.64 ±0.07	3.33 ±0.19	165.8 ±8.3	167.7 ±5.8	153.1 ±11.9
GNCA4-2	2.67 ±0.07	2.64 ±0.07	3.32 ±0.18	165.9 ±6.9	169.0 ±5.6	154.2 ±12.7
GNCA4-3	2.65 ±0.07	2.65 ±0.06	3.37 ±0.20	165.6 ±7.3	167.0 ±6.1	156.2 ±12.2
GNCA4-4	4.25 ±0.96	2.65 ±0.07	3.37 ±0.20	129.8 ±21.5	167.1 ±7.1	151.0 ±15.3
GNCA4-5	2.80 ±0.62	2.64 ±0.07	3.36 ±0.18	163.6 ±10.3	167.6 ±6.0	154.7 ±10.1
GNCA4-6	2.68 ±0.10	2.64 ±0.06	3.23 ±0.16	166.0 ±6.5	167.4 ±6.0	154.9 ±10.2
GNCA4-7	2.82 ±0.64	2.64 ±0.06	3.28 ±0.21	162.1 ±10.8	168.0 ±5.7	155.1 ±13.0
GNCA4-8	2.64 ±0.07	2.64 ±0.07	3.33 ±0.20	165.8 ±6.3	167.7 ±5.6	154.7 ±12.7
GNCA4-9	3.04 ±0.87	2.64 ±0.06	3.25 ±0.17	157.1 ±17.7	168.7 ±5.4	155.1 ±11.9
GNCA4-10	2.65 ±0.08	2.64 ±0.07	3.32 ±0.23	166.0 ±5.8	168.0 ±5.3	154.0 ±11.6
GNCA4-11	2.66 ±0.08	2.64 ±0.06	3.30 ±0.17	165.6 ±7.0	167.2 ±6.6	154.4 ±9.4
GNCA4-12	2.66 ±0.08	2.64 ±0.07	3.25 ±0.16	166.2 ±6.7	168.5 ±5.5	153.9 ±10.0
GNCA4-13	3.02 ±0.77	2.65 ±0.06	3.32 ±0.24	154.1 ±20.3	167.7 ±6.1	149.5 ±21.7
GNCA4-14	2.66 ±0.08	2.65 ±0.06	3.27 ±0.19	166.3 ±5.7	167.4 ±6.1	152.6 ±10.9
GNCA4-15	2.64 ±0.07	2.64 ±0.07	3.34 ±0.18	166.5 ±6.3	167.3 ±6.4	155.4 ±11.5
GNCA4-16	2.64 ±0.07	2.64 ±0.06	3.23 ±0.15	164.9 ±6.4	168.4 ±5.8	152.2 ±9.0
GNCA4-17	2.99 ±0.78	2.64 ±0.06	3.30 ±0.19	159.1 ±16.7	168.0 ±6.4	154.7 ±16.3
GNCA4-18	2.66 ±0.08	2.64 ±0.07	3.26 ±0.19	167.1 ±5.9	167.8 ±5.6	152.0 ±14.4
GNCA4-19	2.65 ±0.08	2.66 ±0.08	3.26 ±0.19	166.5 ±6.0	166.7 ±6.5	155.0 ±10.2
GNCA4-20	2.67 ±0.07	2.65 ±0.07	3.24 ±0.17	164.3 ±8.0	166.7 ±6.3	154.0 ±10.0

[a] D-A distances are presented in Å and D-H..A angles are presented in °. Data is shown as average values and standard deviations over 30 independent trajectories. MC, TS and PC denote the Michaelis complex, transition state, and product complex, respectively.

Supplementary References

1. O. Khersonsky, R. Lipsh, Z. Avizemer, Y. Ashani, M. Goldsmith, H. Leader, O. Dym, S. Rogotner, D. L. Trudeau, J. Prilusky, P. Amengual-Rigo, V. Guallar, D. S. Tawfik and S. J. Fleishman, *Mol. Cell*, 2018, **72**, 178-186.e175.
2. F. S. Lee and A. Warshel, *J. Chem. Phys.*, 1992, **97**, 3100.
3. I. Muegge, H. Tao and A. Warshel, *Protein Eng. Des. Sel.*, 1997, **10**, 1363-1372.
4. Y. S. Kulkarni, Q. Liao, D. Petrović, D. M. Krüger, B. Strodel, T. L. Amyes, J. P. Richard and S. C. L. Kamerlin, *J. Am. Chem. Soc.*, 2017, **139**, 10514-10525.
5. Y. S. Kulkarni, Q. Liao, F. Byléhn, T. L. Amyes, J. P. Richard and S. C. L. Kamerlin, *J. Am. Chem. Soc.*, 2018, **140**, 3854-3857.
6. Y. S. Kulkarni, T. L. Amyes , J. P. Richard and S. C. L. Kamerlin, *J. Am. Chem. Soc.*, 2019, **141**, 16139-16150.
7. V. A. Risso, S. Martinez-Rodriguez, A. M. Candel, D. M. Krüger, D. Pantoja-Uceda, M. Ortega-Muñoz, F. Santoyo-Gonzlez, E. A. Gaucher, S. C. L. Kamerlin, M. Bruix, J. A. Gavira and J. M. Sanchez-Ruiz, *Nat. Commun.*, 2017, **8**, 16113.
8. A. V. Morozov and T. Kortemme, *Adv. Protein Chem.*, 2005, **72**, 1-38.
