# Supplementary material for: Enhancing a de novo enzyme activity by computationally-focused ultra-low-throughput screening
Source: Chem Sci. 2020 May 19;11(24):6134–48. doi: 10.1039/d0sc01935f (PMC7407621; doi:10.1039/d0sc01935f)
Supplement: Supplementary file 2 [file SC-011-D0SC01935F-s002.pdf]

This journal is © The Royal Society of Chemistry 2020

SupplementaryData

serial_number	48A	50A	250A	256A	260A	261A	285A	286A	287A	290A	291A	total_score
'01010101010101010101	V	D	I	R	L	V	L	V	V	W	H	-906.616
'0101010202020101040104	V	D	I	H	F	I	L	V	L	W	I	-917.026
'0101040102030101010106	V	D	V	R	F	L	L	V	V	W	L	-916.926
'0101010202020101010101	V	D	I	H	F	I	L	V	V	W	H	-916.868
'0101030102020101040104	V	D	M	R	F	I	L	V	L	W	I	-916.714
'0101010202030101010104	V	D	I	H	F	L	L	V	V	W	I	-916.472
'0101040102020103010104	V	D	V	R	F	I	L	I	V	W	I	-916.394
'0101030202030101010105	V	D	M	H	F	L	L	V	V	W	K	-916.082
'0101010102020101010105	V	D	I	R	F	I	L	V	V	W	K	-916.056
'0101030202020101020101	V	D	M	H	F	I	L	V	A	W	H	-915.92
'0101010102030101040104	V	D	I	R	F	L	L	V	L	W	I	-915.728
'0101040202030101070101	V	D	V	H	F	L	L	V	T	W	H	-915.696
'0101040202030101010112	V	D	V	H	F	L	L	V	V	W	V	-915.629
'0101030102030101010101	V	D	M	R	F	L	L	V	V	W	H	-915.391
'0101010102030101020109	V	D	I	R	F	L	L	V	A	W	Q	-915.354
'0201010102030103010104	I	D	I	R	F	L	L	I	V	W	I	-915.214
'0101040105030101040108	V	D	V	R	V	L	L	V	L	W	N	-915.183
'0101030205020101010104	V	D	M	H	V	I	L	V	V	W	I	-915.135
'0101040103020103010106	V	D	V	R	I	I	L	I	V	W	L	-915.116
'0101040202020103010101	V	D	V	H	F	I	L	I	V	W	H	-915.115
'0101010203020101070108	V	D	I	H	I	I	L	V	T	W	N	-915.018
'0101010202030101070110	V	D	I	H	F	L	L	V	T	W	R	-914.945
'0101040103030101040112	V	D	V	R	I	L	L	V	L	W	V	-914.942
'0201010103020101010106	I	D	I	R	I	I	L	V	V	W	L	-914.936
'0201040103030101010104	I	D	V	R	I	L	L	V	V	W	I	-914.906
'0101030105020103010105	V	D	M	R	V	I	L	I	V	W	K	-914.852
'0101010202030101040105	V	D	I	H	F	L	L	V	L	W	K	-914.823
'0101040102030101050105	V	D	V	R	F	L	L	V	M	W	K	-914.8
'0101010202020101070112	V	D	I	H	F	I	L	V	T	W	V	-914.796
'0101010403020101040106	V	D	I	Q	I	I	L	V	L	W	L	-914.775
'0101010102010103010108	V	D	I	R	F	V	L	I	V	W	N	-914.763
'0101010103020101040112	V	D	I	R	I	I	L	V	L	W	V	-914.76
'0101040102020103070101	V	D	V	R	F	I	L	I	T	W	H	-914.715
'0101040102010103040101	V	D	V	R	F	V	L	I	L	W	H	-914.689
'0101010102020101020112	V	D	I	R	F	I	L	V	A	W	V	-914.683

**'0101030102020101020105**	V	D	M	R	F	I	L	V	A	W	K	-914.627
**'0101010102020103030105**	V	D	I	R	F	I	L	I	I	W	K	-914.601
**'0101030102030101070106**	V	D	M	R	F	L	L	V	T	W	L	-914.582
**'0101010402020103010105**	V	D	I	Q	F	I	L	I	V	W	K	-914.579
**'0101030102010103040104**	V	D	M	R	F	V	L	I	L	W	I	-914.498
**'0101010202030103010109**	V	D	I	H	F	L	L	I	V	W	Q	-914.462
**'0101030201020101010105**	V	D	M	H	L	I	L	V	V	W	K	-914.441
**'0101030103020101010112**	V	D	M	R	I	I	L	V	V	W	V	-914.42
**'0101030202030101050101**	V	D	M	H	F	L	L	V	M	W	H	-914.41
**'0101010102020103020111**	V	D	I	R	F	I	L	I	A	W	T	-914.408
**'0101030102020103010112**	V	D	M	R	F	I	L	I	V	W	V	-914.4
**'0101040102010103010110**	V	D	V	R	F	V	L	I	V	W	R	-914.375
**'0101040203010103010101**	V	D	V	H	I	V	L	I	V	W	H	-914.352
**'0101010302040101040104**	V	D	I	K	F	M	L	V	L	W	I	-914.346
**'0101010102030101030110**	V	D	I	R	F	L	L	V	I	W	R	-914.339
**'0101010205020101020101**	V	D	I	H	V	I	L	V	A	W	H	-914.319
**'0101040102030101040107**	V	D	V	R	F	L	L	V	L	W	M	-914.272
**'0101030203030101010104**	V	D	M	H	I	L	L	V	V	W	I	-914.261
**'0101030102020101070109**	V	D	M	R	F	I	L	V	T	W	Q	-914.25
**'0101010105020103030106**	V	D	I	R	V	I	L	I	I	W	L	-914.227
**'0101020102020101010106**	V	D	L	R	F	I	L	V	V	W	L	-914.186
**'0101030103020101040109**	V	D	M	R	I	I	L	V	L	W	Q	-914.16
**'0101010302030101020101**	V	D	I	K	F	L	L	V	A	W	H	-914.149
**'0101040105030101020104**	V	D	V	R	V	L	L	V	A	W	I	-914.108
**'0101010102020103040101**	V	D	I	R	F	I	L	I	L	W	H	-914.103
**'0101010105020103040102**	V	D	I	R	V	I	L	I	L	W	E	-914.094
**'0101010302030103010105**	V	D	I	K	F	L	L	I	V	W	K	-914.061
**'0101040105020103010101**	V	D	V	R	V	I	L	I	V	W	H	-914.01
**'0101030105020101070106**	V	D	M	R	V	I	L	V	T	W	L	-913.984
**'0101030105020101010108**	V	D	M	R	V	I	L	V	V	W	N	-913.93
**'0101010102040101070101**	V	D	I	R	F	M	L	V	T	W	H	-913.897
**'0101040103030101020108**	V	D	V	R	I	L	L	V	A	W	N	-913.896
**'0101030105010103040106**	V	D	M	R	V	V	L	I	L	W	L	-913.879
**'0101040102010104040104**	V	D	V	R	F	V	L	L	L	W	I	-913.879
**'0101040102030201010108**	V	D	V	R	F	L	A	V	V	W	N	-913.87
**'0101030102030101040110**	V	D	M	R	F	L	L	V	L	W	R	-913.858
**'0101040105010103040108**	V	D	V	R	V	V	L	I	L	W	N	-913.849

**'0101040102030103010105**	V	D	V	R	F	L	L	I	V	W	K	-913.841
**'0101030102020101050106**	V	D	M	R	F	I	L	V	M	W	L	-913.836
**'0101010102030103030104**	V	D	I	R	F	L	L	I	I	W	I	-913.823
**'0101040102010101040106**	V	D	V	R	F	V	L	V	L	W	L	-913.812
**'0201040105030101010109**	I	D	V	R	V	L	L	V	V	W	Q	-913.799
**'0101030302020101010106**	V	D	M	K	F	I	L	V	V	W	L	-913.773
**'0101040105020101010106**	V	D	V	R	V	I	L	V	V	W	L	-913.77
**'0101030205020101040101**	V	D	M	H	V	I	L	V	L	W	H	-913.764
**'0101010102030103020106**	V	D	I	R	F	L	L	I	A	W	L	-913.757
**'0101010102030101050101**	V	D	I	R	F	L	L	V	M	W	H	-913.75
**'0201010105010103040106**	I	D	I	R	V	V	L	I	L	W	L	-913.698
**'0101030402020101070101**	V	D	M	Q	F	I	L	V	T	W	H	-913.677
**'0101010102020101040111**	V	D	I	R	F	I	L	V	L	W	T	-913.665
**'0101040402030101040101**	V	D	V	Q	F	L	L	V	L	W	H	-913.649
**'0201010103020101070104**	I	D	I	R	I	I	L	V	T	W	I	-913.634
**'0101010203020101030110**	V	D	I	H	I	I	L	V	I	W	R	-913.618
**'0101010202040101010108**	V	D	I	H	F	M	L	V	V	W	N	-913.604
**'0101040103010103040109**	V	D	V	R	I	V	L	I	L	W	Q	-913.595
**'0101010105020101030110**	V	D	I	R	V	I	L	V	I	W	R	-913.592
**'0101010202030101030108**	V	D	I	H	F	L	L	V	I	W	N	-913.581
**'0101040203030101020101**	V	D	V	H	I	L	L	V	A	W	H	-913.554
**'0101040103010103030105**	V	D	V	R	I	V	L	I	I	W	K	-913.542
**'0101030103020101030104**	V	D	M	R	I	I	L	V	I	W	I	-913.527
**'0101040103030101030104**	V	D	V	R	I	L	L	V	I	W	I	-913.521
**'0101030105030101040104**	V	D	M	R	V	L	L	V	L	W	I	-913.518
**'0101010302020103010108**	V	D	I	K	F	I	L	I	V	W	N	-913.516
**'0101010102010103030102**	V	D	I	R	F	V	L	I	I	W	E	-913.507
**'0101040103020101040105**	V	D	V	R	I	I	L	V	L	W	K	-913.484
**'0101010203030101020112**	V	D	I	H	I	L	L	V	A	W	V	-913.481
**'0201010105030101010106**	I	D	I	R	V	L	L	V	V	W	L	-913.465
**'0201040103030101020101**	I	D	V	R	I	L	L	V	A	W	H	-913.444
**'0101030102030103010110**	V	D	M	R	F	L	L	I	V	W	R	-913.44
**'0101010105020101020106**	V	D	I	R	V	I	L	V	A	W	L	-913.425
**'0101030103010103010110**	V	D	M	R	I	V	L	I	V	W	R	-913.417
**'0101010102020101030107**	V	D	I	R	F	I	L	V	I	W	M	-913.413
**'0101030105010103030104**	V	D	M	R	V	V	L	I	I	W	I	-913.403
**'0101040102010103030104**	V	D	V	R	F	V	L	I	I	W	I	-913.396

**'0101010102030201040106**	V	D	I	R	F	L	A	V	L	W	L	-913.365
**'0101030102010103010106**	V	D	M	R	F	V	L	I	V	W	L	-913.359
**'0101010202040101040106**	V	D	I	H	F	M	L	V	L	W	L	-913.351
**'0201010202030101010112**	I	D	I	H	F	L	L	V	V	W	V	-913.334
**'0101030105010103020105**	V	D	M	R	V	V	L	I	A	W	K	-913.33
**'0101020102030101040106**	V	D	L	R	F	L	L	V	L	W	L	-913.322
**'0101020202020101010104**	V	D	L	H	F	I	L	V	V	W	I	-913.319
**'0101030202010103010112**	V	D	M	H	F	V	L	I	V	W	V	-913.3
**'0101010402030103010104**	V	D	I	Q	F	L	L	I	V	W	I	-913.294
**'0101010205040101040104**	V	D	I	H	V	M	L	V	L	W	I	-913.271
**'0101010202020101020108**	V	D	I	H	F	I	L	V	A	W	N	-913.242
**'0101010105020101050101**	V	D	I	R	V	I	L	V	M	W	H	-913.24
**'0201010102030101040107**	I	D	I	R	F	L	L	V	L	W	M	-913.239
**'0101010105020103050112**	V	D	I	R	V	I	L	I	M	W	V	-913.236
**'0201010102020101040105**	I	D	I	R	F	I	L	V	L	W	K	-913.228
**'0201010105030101020105**	I	D	I	R	V	L	L	V	A	W	K	-913.225
**'0101040105020101070104**	V	D	V	R	V	I	L	V	T	W	I	-913.218
**'0101030102040101040106**	V	D	M	R	F	M	L	V	L	W	L	-913.213
**'0101040203030101010106**	V	D	V	H	I	L	L	V	V	W	L	-913.2
**'0101010102010103040106**	V	D	I	R	F	V	L	I	L	W	L	-913.198
**'0101030205030101070101**	V	D	M	H	V	L	L	V	T	W	H	-913.168
**'0101010402020101070111**	V	D	I	Q	F	I	L	V	T	W	T	-913.167
**'0101040103030103040101**	V	D	V	R	I	L	L	I	L	W	H	-913.166
**'0101040203020101010104**	V	D	V	H	I	I	L	V	V	W	I	-913.165
**'0101020102030101010109**	V	D	L	R	F	L	L	V	V	W	Q	-913.15
**'0101040105020101040110**	V	D	V	R	V	I	L	V	L	W	R	-913.146
**'0201010102030101010111**	I	D	I	R	F	L	L	V	V	W	T	-913.085
**'0101010402030101040112**	V	D	I	Q	F	L	L	V	L	W	V	-913.069
**'0301040105020101010101**	L	D	V	R	V	I	L	V	V	W	H	-913.054
**'0101040102020101010102**	V	D	V	R	F	I	L	V	V	W	E	-913.051
**'0101010103020103040109**	V	D	I	R	I	I	L	I	L	W	Q	-913.022
**'0101040105010103030110**	V	D	V	R	V	V	L	I	I	W	R	-913.01
**'0101040105030101070107**	V	D	V	R	V	L	L	V	T	W	M	-912.997
**'0101030105020101040105**	V	D	M	R	V	I	L	V	L	W	K	-912.992
**'0101010405020101040107**	V	D	I	Q	V	I	L	V	L	W	M	-912.985
**'0101010105010103030109**	V	D	I	R	V	V	L	I	I	W	Q	-912.983
**'0101010402020101040101**	V	D	I	Q	F	I	L	V	L	W	H	-912.928

'0101020105020101040101	V	D	L	R	V	I	L	V	L	W	H	-912.923
'0101030402010101040104	V	D	M	Q	F	V	L	V	L	W	I	-912.92
'0101010202010103030106	V	D	I	H	F	V	L	I	I	W	L	-912.918
'0101010305010103040104	V	D	I	K	V	V	L	I	L	W	I	-912.914
'0201040105030103010101	I	D	V	R	V	L	L	I	V	W	H	-912.911
'0101010205020201040101	V	D	I	H	V	I	A	V	L	W	H	-912.872
'0101040105020104010104	V	D	V	R	V	I	L	L	V	W	I	-912.866
'0101030103030101020105	V	D	M	R	I	L	L	V	A	W	K	-912.86
'0101010302020101060105	V	D	I	K	F	I	L	V	S	W	K	-912.856
'0101030205040101010106	V	D	M	H	V	M	L	V	V	W	L	-912.851
'0101030201030101010108	V	D	M	H	L	L	L	V	V	W	N	-912.844
'0201010102030101070104	I	D	I	R	F	L	L	V	T	W	I	-912.802
'0101040105010103070102	V	D	V	R	V	V	L	I	T	W	E	-912.798
'0101010103020101030108	V	D	I	R	I	I	L	V	I	W	N	-912.796
'0101010102030103040112	V	D	I	R	F	L	L	I	L	W	V	-912.792
'0101040205030101040101	V	D	V	H	V	L	L	V	L	W	H	-912.786
'0101040102010103050105	V	D	V	R	F	V	L	I	M	W	K	-912.781
'0101040105030101050106	V	D	V	R	V	L	L	V	M	W	L	-912.775
'0101020102010103010101	V	D	L	R	F	V	L	I	V	W	H	-912.773
'0101030402030101010108	V	D	M	Q	F	L	L	V	V	W	N	-912.761
'0201010105020101040101	I	D	I	R	V	I	L	V	L	W	H	-912.755
'0101030102020103050101	V	D	M	R	F	I	L	I	M	W	H	-912.749
'0101030102030101020111	V	D	M	R	F	L	L	V	A	W	T	-912.748
'0101030201020101040106	V	D	M	H	L	I	L	V	L	W	L	-912.741
'0101010105020103010109	V	D	I	R	V	I	L	I	V	W	Q	-912.739
'0101040102010101070105	V	D	V	R	F	V	L	V	T	W	K	-912.693
'0101030103020201010101	V	D	M	R	I	I	A	V	V	W	H	-912.677
'0101030201020101020108	V	D	M	H	L	I	L	V	A	W	N	-912.667
'0101030103020101070110	V	D	M	R	I	I	L	V	T	W	R	-912.666
'0101010105020104040104	V	D	I	R	V	I	L	L	L	W	I	-912.662
'0101030102010101040109	V	D	M	R	F	V	L	V	L	W	Q	-912.658
'0101010102030101010107	V	D	I	R	F	L	L	V	V	W	M	-912.652
'0201040102010103010104	I	D	V	R	F	V	L	I	V	W	I	-912.649
'0101010102020101060104	V	D	I	R	F	I	L	V	S	W	I	-912.636
'0101010102010203010102	V	D	I	R	F	V	A	I	V	W	E	-912.636
'0101030103030101040102	V	D	M	R	I	L	L	V	L	W	E	-912.62
'0101030103010103040108	V	D	M	R	I	V	L	I	L	W	N	-912.617

'0201010102020101070110	I	D	I	R	F	I	L	V	T	W	R	-912.592
'0101030202030201010101	V	D	M	H	F	L	A	V	V	W	H	-912.569
'0101010203020103010106	V	D	I	H	I	I	L	I	V	W	L	-912.546
'0101010402010103030110	V	D	I	Q	F	V	L	I	I	W	R	-912.54
'0101010303020101010103	V	D	I	K	I	I	L	V	V	W	F	-912.538
'0101010205020101070102	V	D	I	H	V	I	L	V	T	W	E	-912.531
'0101010102010101040112	V	D	I	R	F	V	L	V	L	W	V	-912.526
'0201010105030101040108	I	D	I	R	V	L	L	V	L	W	N	-912.519
'0101010103020101010110	V	D	I	R	I	I	L	V	V	W	R	-912.513
'0201010202020101020101	I	D	I	H	F	I	L	V	A	W	H	-912.5
'0101040302030103010101	V	D	V	K	F	L	L	I	V	W	H	-912.497
'0101040303030101010110	V	D	V	K	I	L	L	V	V	W	R	-912.493
'0201010205020101010104	I	D	I	H	V	I	L	V	V	W	I	-912.483
'0201010202010103010104	I	D	I	H	F	V	L	I	V	W	I	-912.475
'0101010405020101030109	V	D	I	Q	V	I	L	V	I	W	Q	-912.459
'0101030102010101020108	V	D	M	R	F	V	L	V	A	W	N	-912.449
'0101030205020103010101	V	D	M	H	V	I	L	I	V	W	H	-912.449
'0101010205020101030112	V	D	I	H	V	I	L	V	I	W	V	-912.446
'0101030105020101030102	V	D	M	R	V	I	L	V	I	W	E	-912.434
'0101030102010104040105	V	D	M	R	F	V	L	L	L	W	K	-912.432
'0101010203010103030110	V	D	I	H	I	V	L	I	I	W	R	-912.432
'0201010205030103010101	I	D	I	H	V	L	L	I	V	W	H	-912.432
'0101040102020101070110	V	D	V	R	F	I	L	V	T	W	R	-912.423
'0101030102010103070109	V	D	M	R	F	V	L	I	T	W	Q	-912.408
'0201030105020101010105	I	D	M	R	V	I	L	V	V	W	K	-912.402
'0201010102030102010110	I	D	I	R	F	L	L	A	V	W	R	-912.381
'0101040105010103060105	V	D	V	R	V	V	L	I	S	W	K	-912.37
'0101040302010101040104	V	D	V	K	F	V	L	V	L	W	I	-912.365
'0101010303020101040109	V	D	I	K	I	I	L	V	L	W	Q	-912.365
'0101010205010104010101	V	D	I	H	V	V	L	L	V	W	H	-912.355
'0101040105030201010104	V	D	V	R	V	L	A	V	V	W	I	-912.348
'0101030103030101010110	V	D	M	R	I	L	L	V	V	W	R	-912.346
'0301040102020101010112	L	D	V	R	F	I	L	V	V	W	V	-912.342
'0101040302020101010101	V	D	V	K	F	I	L	V	V	W	H	-912.332
'0101010402030103070101	V	D	I	Q	F	L	L	I	T	W	H	-912.322
'0101030305010103010104	V	D	M	K	V	V	L	I	V	W	I	-912.32
'0101030102040101010104	V	D	M	R	F	M	L	V	V	W	I	-912.319

'0101010103020101050103	V	D	I	R	I	I	L	V	M	W	F	-912.295
'0101020102020101040105	V	D	L	R	F	I	L	V	L	W	K	-912.291
'0101010202030103020101	V	D	I	H	F	L	L	I	A	W	H	-912.282
'0101040104010103040110	V	D	V	R	M	V	L	I	L	W	R	-912.279
'0201040105010101040104	I	D	V	R	V	V	L	V	L	W	I	-912.269
'0101030102030101030108	V	D	M	R	F	L	L	V	I	W	N	-912.252
'0101040103010104010108	V	D	V	R	I	V	L	L	V	W	N	-912.25
'0101010202030203010101	V	D	I	H	F	L	A	I	V	W	H	-912.248
'0101040102010201040104	V	D	V	R	F	V	A	V	L	W	I	-912.246
'0101040202010101040102	V	D	V	H	F	V	L	V	L	W	E	-912.242
'0101010105030103040101	V	D	I	R	V	L	L	I	L	W	H	-912.234
'0101030402010103010110	V	D	M	Q	F	V	L	I	V	W	R	-912.232
'0301040103020101010110	L	D	V	R	I	I	L	V	V	W	R	-912.219
'0101010102020401010106	V	D	I	R	F	I	W	V	V	W	L	-912.214
'0101040205010101040105	V	D	V	H	V	V	L	V	L	W	K	-912.209
'0101030202020101010109	V	D	M	H	F	I	L	V	V	W	Q	-912.207
'0101020102020101020108	V	D	L	R	F	I	L	V	A	W	N	-912.203
'0101040102020101030106	V	D	V	R	F	I	L	V	I	W	L	-912.199
'0101010105040101020111	V	D	I	R	V	M	L	V	A	W	T	-912.197
'0101030102030101050109	V	D	M	R	F	L	L	V	M	W	Q	-912.191
'0101040103030101050107	V	D	V	R	I	L	L	V	M	W	M	-912.19
'0101010102010104070108	V	D	I	R	F	V	L	L	T	W	N	-912.17
'0101010105020104010108	V	D	I	R	V	I	L	L	V	W	N	-912.166
'0101010205020103010111	V	D	I	H	V	I	L	I	V	W	T	-912.162
'0101010305020101020104	V	D	I	K	V	I	L	V	A	W	I	-912.16
'0101030202010101010106	V	D	M	H	F	V	L	V	V	W	L	-912.16
'0201010102020101020104	I	D	I	R	F	I	L	V	A	W	I	-912.159
'0201030102030102010101	I	D	M	R	F	L	L	A	V	W	H	-912.152
'0101040102020101020104	V	D	V	R	F	I	L	V	A	W	I	-912.141
'0101030102030101060112	V	D	M	R	F	L	L	V	S	W	V	-912.136
'0101040102020104010110	V	D	V	R	F	I	L	L	V	W	R	-912.135
'0101030205010101010101	V	D	M	H	V	V	L	V	V	W	H	-912.134
'0101030105040101010101	V	D	M	R	V	M	L	V	V	W	H	-912.129
'0201030102010101040106	I	D	M	R	F	V	L	V	L	W	L	-912.093
'0101010102020101070106	V	D	I	R	F	I	L	V	T	W	L	-912.092
'0101010203010103040104	V	D	I	H	I	V	L	I	L	W	I	-912.091
'0101040103020101010101	V	D	V	R	I	I	L	V	V	W	H	-912.07

'0101010102020102040106	V	D	I	R	F	I	L	A	L	W	L	-912.066
'0101010102030201070108	V	D	I	R	F	L	A	V	T	W	N	-912.052
'0201030102020101010101	I	D	M	R	F	I	L	V	V	W	H	-912.049
'0101010205030101040109	V	D	I	H	V	L	L	V	L	W	Q	-912.049
'0301010105020101040104	L	D	I	R	V	I	L	V	L	W	I	-912.043
'0101040303020103010101	V	D	V	K	I	I	L	I	V	W	H	-912.043
'0101030305020101070101	V	D	M	K	V	I	L	V	T	W	H	-912.019
'0101010103030101040111	V	D	I	R	I	L	L	V	L	W	T	-912.018
'0101040102020101060101	V	D	V	R	F	I	L	V	S	W	H	-912.017
'0101030203020101010111	V	D	M	H	I	I	L	V	V	W	T	-911.99
'0101010102030301010104	V	D	I	R	F	L	V	V	V	W	I	-911.987
'0101040302010103010104	V	D	V	K	F	V	L	I	V	W	I	-911.986
'0101040302030101010105	V	D	V	K	F	L	L	V	V	W	K	-911.982
'0101040205030101010104	V	D	V	H	V	L	L	V	V	W	I	-911.978
'0101010203010101040110	V	D	I	H	I	V	L	V	L	W	R	-911.97
'0201040405030101010101	I	D	V	Q	V	L	L	V	V	W	H	-911.966
'0101030105020103020101	V	D	M	R	V	I	L	I	A	W	H	-911.96
'0101030105030101070111	V	D	M	R	V	L	L	V	T	W	T	-911.958
'0101010103030301010106	V	D	I	R	I	L	V	V	V	W	L	-911.948
'0101010205040101070109	V	D	I	H	V	M	L	V	T	W	Q	-911.928
'0101010202010104020101	V	D	I	H	F	V	L	L	A	W	H	-911.923
'0301010103020101040105	L	D	I	R	I	I	L	V	L	W	K	-911.92
'0201010102010103010101	I	D	I	R	F	V	L	I	V	W	H	-911.915
'0101010105020201040112	V	D	I	R	V	I	A	V	L	W	V	-911.908
'0201030202030101010101	I	D	M	H	F	L	L	V	V	W	H	-911.902
'0101030102010103030112	V	D	M	R	F	V	L	I	I	W	V	-911.9
'0201010102020102010108	I	D	I	R	F	I	L	A	V	W	N	-911.894
'0101010205040101030105	V	D	I	H	V	M	L	V	I	W	K	-911.892
'0101030205030101010102	V	D	M	H	V	L	L	V	V	W	E	-911.888
'0101020102030101070110	V	D	L	R	F	L	L	V	T	W	R	-911.886
'0101040103020103050101	V	D	V	R	I	I	L	I	M	W	H	-911.882
'0101020105020101010105	V	D	L	R	V	I	L	V	V	W	K	-911.879
'0101040102020105010101	V	D	V	R	F	I	L	M	V	W	H	-911.878
'0101040103010103050110	V	D	V	R	I	V	L	I	M	W	R	-911.876
'0101010102010103050104	V	D	I	R	F	V	L	I	M	W	I	-911.873
'0101040102010104020101	V	D	V	R	F	V	L	L	A	W	H	-911.871
'0101010103030201040101	V	D	I	R	I	L	A	V	L	W	H	-911.871

**'0101010205040101010110**	V	D	I	H	V	M	L	V	V	W	R	-911.867
**'0101040103030301020101**	V	D	V	R	I	L	V	V	A	W	H	-911.85
**'0101040105010201040106**	V	D	V	R	V	V	A	V	L	W	L	-911.845
**'0101020302010103010105**	V	D	L	K	F	V	L	I	V	W	K	-911.826
**'0101010103020101070105**	V	D	I	R	I	I	L	V	T	W	K	-911.825
**'0101010203030101050111**	V	D	I	H	I	L	L	V	M	W	T	-911.825
**'0101040402010101020108**	V	D	V	Q	F	V	L	V	A	W	N	-911.819
**'0101010205020103040101**	V	D	I	H	V	I	L	I	L	W	H	-911.809
**'0101010102020103070104**	V	D	I	R	F	I	L	I	T	W	I	-911.808
**'0101010105030201070112**	V	D	I	R	V	L	A	V	T	W	V	-911.808
**'0101010205010103040105**	V	D	I	H	V	V	L	I	L	W	K	-911.795
**'0101030201030101040112**	V	D	M	H	L	L	L	V	L	W	V	-911.794
**'0101010102030101060105**	V	D	I	R	F	L	L	V	S	W	K	-911.785
**'0201010102040101010109**	I	D	I	R	F	M	L	V	V	W	Q	-911.782
**'0101040102010103020108**	V	D	V	R	F	V	L	I	A	W	N	-911.78
**'0101010103020103010101**	V	D	I	R	I	I	L	I	V	W	H	-911.779
**'0101010102020104040105**	V	D	I	R	F	I	L	L	L	W	K	-911.779
**'0101020202010103010109**	V	D	L	H	F	V	L	I	V	W	Q	-911.775
**'0201010105020101070106**	I	D	I	R	V	I	L	V	T	W	L	-911.769
**'0101030105010101050105**	V	D	M	R	V	V	L	V	M	W	K	-911.733
**'0101030103010104010106**	V	D	M	R	I	V	L	L	V	W	L	-911.731
**'0101010405040101040106**	V	D	I	Q	V	M	L	V	L	W	L	-911.728
**'0301010202020101010104**	L	D	I	H	F	I	L	V	V	W	I	-911.725
**'0201010105010103020104**	I	D	I	R	V	V	L	I	A	W	I	-911.723
**'0101030204020101010112**	V	D	M	H	M	I	L	V	V	W	V	-911.722
**'0101040105030101030102**	V	D	V	R	V	L	L	V	I	W	E	-911.717
**'0101040305010101070104**	V	D	V	K	V	V	L	V	T	W	I	-911.716
**'0101030201030101070104**	V	D	M	H	L	L	L	V	T	W	I	-911.715
**'0101030203020101030101**	V	D	M	H	I	I	L	V	I	W	H	-911.71
**'0101030205010104010112**	V	D	M	H	V	V	L	L	V	W	V	-911.709
**'0101010203010203010101**	V	D	I	H	I	V	A	I	V	W	H	-911.705
**'0101030103020103010108**	V	D	M	R	I	I	L	I	V	W	N	-911.704
**'0101010305030101040105**	V	D	I	K	V	L	L	V	L	W	K	-911.702
**'0101030104020101040101**	V	D	M	R	M	I	L	V	L	W	H	-911.701
**'0101040105010104010102**	V	D	V	R	V	V	L	L	V	W	E	-911.701
**'0101010202010103040109**	V	D	I	H	F	V	L	I	L	W	Q	-911.7
**'0101010105020104020105**	V	D	I	R	V	I	L	L	A	W	K	-911.699

'0101040205020101010105	V	D	V	H	V	I	L	V	V	W	K	-911.697
'0301040102010101040104	L	D	V	R	F	V	L	V	L	W	I	-911.695
'0101010103020301010105	V	D	I	R	I	I	V	V	V	W	K	-911.687
'0101030103030101030101	V	D	M	R	I	L	L	V	I	W	H	-911.685
'0101040405010103040101	V	D	V	Q	V	V	L	I	L	W	H	-911.68
'0101020102020101070104	V	D	L	R	F	I	L	V	T	W	I	-911.678
'0101040103020101070108	V	D	V	R	I	I	L	V	T	W	N	-911.674
'0101010103030101010104	V	D	I	R	I	L	L	V	V	W	I	-911.674
'0101010302040101010103	V	D	I	K	F	M	L	V	V	W	F	-911.672
'0101040105010103020101	V	D	V	R	V	V	L	I	A	W	H	-911.669
'0201010303020101010105	I	D	I	K	I	I	L	V	V	W	K	-911.66
'0101010105040101040107	V	D	I	R	V	M	L	V	L	W	M	-911.647
'0101040203010101070104	V	D	V	H	I	V	L	V	T	W	I	-911.646
'0101010202020102010105	V	D	I	H	F	I	L	A	V	W	K	-911.646
'0101020102010101010108	V	D	L	R	F	V	L	V	V	W	N	-911.646
'0201040103010103010101	I	D	V	R	I	V	L	I	V	W	H	-911.644
'0101040102010104010106	V	D	V	R	F	V	L	L	V	W	L	-911.642
'0101030205010101040102	V	D	M	H	V	V	L	V	L	W	E	-911.642
'0101040103010101050106	V	D	V	R	I	V	L	V	M	W	L	-911.636
'0301010105020103010104	L	D	I	R	V	I	L	I	V	W	I	-911.632
'0201040105030101040101	I	D	V	R	V	L	L	V	L	W	H	-911.621
'0101030403020101020101	V	D	M	Q	I	I	L	V	A	W	H	-911.619
'0101010302030101070111	V	D	I	K	F	L	L	V	T	W	T	-911.616
'0101010103040101040104	V	D	I	R	I	M	L	V	L	W	I	-911.61
'0101030204030103010101	V	D	M	H	M	L	L	I	V	W	H	-911.597
'0101010203020101010112	V	D	I	H	I	I	L	V	V	W	V	-911.597
'0101020102030101020101	V	D	L	R	F	L	L	V	A	W	H	-911.595
'0101040102030301070101	V	D	V	R	F	L	V	V	T	W	H	-911.59
'0101020203020101010102	V	D	L	H	I	I	L	V	V	W	E	-911.588
'0101010102030103070105	V	D	I	R	F	L	L	I	T	W	K	-911.584
'0201040102040101010104	I	D	V	R	F	M	L	V	V	W	I	-911.569
'0201010102020103010106	I	D	I	R	F	I	L	I	V	W	L	-911.566
'0101010103030103050105	V	D	I	R	I	L	L	I	M	W	K	-911.564
'0101010102030104010112	V	D	I	R	F	L	L	L	V	W	V	-911.556
'0101040405030101010110	V	D	V	Q	V	L	L	V	V	W	R	-911.554
'0101040102030201040101	V	D	V	R	F	L	A	V	L	W	H	-911.554
'0101010102010303010104	V	D	I	R	F	V	V	I	V	W	I	-911.554

**'0301010205020101010112**	L	D	I	H	V	I	L	V	V	W	V	-911.542
**'0101010102020201040109**	V	D	I	R	F	I	A	V	L	W	Q	-911.534
**'0101030402010101010105**	V	D	M	Q	F	V	L	V	V	W	K	-911.531
**'0101030405020101010112**	V	D	M	Q	V	I	L	V	V	W	V	-911.527
**'0101010105040101030104**	V	D	I	R	V	M	L	V	I	W	I	-911.515
**'0101010102010105010109**	V	D	I	R	F	V	L	M	V	W	Q	-911.511
**'0101010302030101050105**	V	D	I	K	F	L	L	V	M	W	K	-911.51
**'0201010405020101010108**	I	D	I	Q	V	I	L	V	V	W	N	-911.51
**'0101030103010101040101**	V	D	M	R	I	V	L	V	L	W	H	-911.506
**'0101030405010103010112**	V	D	M	Q	V	V	L	I	V	W	V	-911.501
**'0101020402020101010101**	V	D	L	Q	F	I	L	V	V	W	H	-911.5
**'0101010402020101030112**	V	D	I	Q	F	I	L	V	I	W	V	-911.496
**'0101030105010104040104**	V	D	M	R	V	V	L	L	L	W	I	-911.49
**'0101040102030101030101**	V	D	V	R	F	L	L	V	I	W	H	-911.485
**'0101010102020101050109**	V	D	I	R	F	I	L	V	M	W	Q	-911.483
**'0101030103020101020102**	V	D	M	R	I	I	L	V	A	W	E	-911.479
**'0201030102030101010110**	I	D	M	R	F	L	L	V	V	W	R	-911.464
**'0101010302030101010106**	V	D	I	K	F	L	L	V	V	W	L	-911.456
**'0101040205020101030101**	V	D	V	H	V	I	L	V	I	W	H	-911.454
**'0101040105020101020102**	V	D	V	R	V	I	L	V	A	W	E	-911.447
**'0101040102030103020101**	V	D	V	R	F	L	L	I	A	W	H	-911.444
**'0201010105030103010103**	I	D	I	R	V	L	L	I	V	W	F	-911.439
**'0101010102040103010105**	V	D	I	R	F	M	L	I	V	W	K	-911.439
**'0201010203020101040101**	I	D	I	H	I	I	L	V	L	W	H	-911.433
**'0201010202040101010104**	I	D	I	H	F	M	L	V	V	W	I	-911.432
**'0101010302020101010102**	V	D	I	K	F	I	L	V	V	W	E	-911.428
**'0101010102020104070101**	V	D	I	R	F	I	L	L	T	W	H	-911.427
**'0201030103020101010108**	I	D	M	R	I	I	L	V	V	W	N	-911.422
**'0101010302040101030106**	V	D	I	K	F	M	L	V	I	W	L	-911.419
**'0101040105030104010105**	V	D	V	R	V	L	L	L	V	W	K	-911.417
**'0101010202010104010105**	V	D	I	H	F	V	L	L	V	W	K	-911.413
**'0101010102020201070104**	V	D	I	R	F	I	A	V	T	W	I	-911.411
**'0101040102010201010109**	V	D	V	R	F	V	A	V	V	W	Q	-911.409
**'0201010102010105010108**	I	D	I	R	F	V	L	M	V	W	N	-911.4
**'0201010105040101040105**	I	D	I	R	V	M	L	V	L	W	K	-911.399
**'0301010202020102010101**	L	D	I	H	F	I	L	A	V	W	H	-911.394
**'0201040105010105010101**	I	D	V	R	V	V	L	M	V	W	H	-911.392

**'010103010504010102010 6**	V	D	M	R	V	M	L	V	A	W	L	-911.375
**'01010104020301010501 06**	V	D	I	Q	F	L	L	V	M	W	L	-911.375
**'02010401050101030701 01**	I	D	V	R	V	V	L	I	T	W	H	-911.373
**'01010302020101010401 08**	V	D	M	H	F	V	L	V	L	W	N	-911.362
**'01010101040101030301 10**	V	D	I	R	M	V	L	I	I	W	R	-911.353
**'01010101050401010501 05**	V	D	I	R	V	M	L	V	M	W	K	-911.343
**'01010102020201010501 07**	V	D	I	H	F	I	L	V	M	W	M	-911.342
**'01010303020101030101 07**	V	D	M	K	F	V	L	I	V	W	M	-911.335
**'01010401050101040201 08**	V	D	V	R	V	V	L	L	A	W	N	-911.334
**'01010103050201010701 09**	V	D	I	K	V	I	L	V	T	W	Q	-911.334
**'01010101030202010301 12**	V	D	I	R	I	I	A	V	I	W	V	-911.328
**'01010301050301030101 01**	V	D	M	R	V	L	L	I	V	W	H	-911.326
**'01010203020201010101 09**	V	D	L	K	F	I	L	V	V	W	Q	-911.325
**'01010402030201010701 01**	V	D	V	H	I	I	L	V	T	W	H	-911.321
**'02010301050101010401 05**	I	D	M	R	V	V	L	V	L	W	K	-911.319
**'02010401020101050101 09**	I	D	V	R	F	V	L	M	V	W	Q	-911.318
**'01010101020101040101 02**	V	D	I	R	F	V	L	L	V	W	E	-911.314
**'01010401020101050101 05**	V	D	V	R	F	V	L	M	V	W	K	-911.302
**'01010201020201010301 12**	V	D	L	R	F	I	L	V	I	W	V	-911.298
**'01010301050401010501 10**	V	D	M	R	V	M	L	V	M	W	R	-911.289
**'01010302040201010701 01**	V	D	M	H	M	I	L	V	T	W	H	-911.272
**'01010101020101010701 04**	V	D	I	R	F	V	L	V	T	W	I	-911.267
**'01010101030201010201 01**	V	D	I	R	I	I	L	V	A	W	H	-911.26
**'01010103020201010701 07**	V	D	I	K	F	I	L	V	T	W	M	-911.258
**'01010102020101050101 04**	V	D	I	H	F	V	L	M	V	W	I	-911.254
**'03010102030201010101 01**	L	D	I	H	I	I	L	V	V	W	H	-911.25
**'01010303020101030701 01**	V	D	M	K	F	V	L	I	T	W	H	-911.25
**'01010401050101040701 04**	V	D	V	R	V	V	L	L	T	W	I	-911.241
**'01010201050101030101 06**	V	D	L	R	V	V	L	I	V	W	L	-911.239
**'01010302010201010701 10**	V	D	M	H	L	I	L	V	T	W	R	-911.224
**'01010301020101040101 09**	V	D	M	R	F	V	L	L	V	W	Q	-911.223
**'01010404020301010101 09**	V	D	V	Q	F	L	L	V	V	W	Q	-911.188
**'01010304020201010101 03**	V	D	M	Q	F	I	L	V	V	W	F	-911.184
**'01010402020303010101 01**	V	D	V	H	F	L	V	V	V	W	H	-911.184
**'01010301020301030401 01**	V	D	M	R	F	L	L	I	L	W	H	-911.18
**'01010101040401010101 06**	V	D	I	R	M	M	L	V	V	W	L	-911.178
**'01010401030201010301 10**	V	D	V	R	I	I	L	V	I	W	R	-911.171

**'0101040201030101040106**	V	D	V	H	L	L	L	V	L	W	L	-911.166
**'0101040105010403010104**	V	D	V	R	V	V	W	I	V	W	I	-911.153
**'0101040304010101040106**	V	D	V	K	M	V	L	V	L	W	L	-911.152
**'0101030102040101070110**	V	D	M	R	F	M	L	V	T	W	R	-911.152
**'0101040105040101040105**	V	D	V	R	V	M	L	V	L	W	K	-911.145
**'0101010102040101050104**	V	D	I	R	F	M	L	V	M	W	I	-911.141
**'0101040103010104020111**	V	D	V	R	I	V	L	L	A	W	T	-911.14
**'0101030105020101020107**	V	D	M	R	V	I	L	V	A	W	M	-911.136
**'0101040105010101040101**	V	D	V	R	V	V	L	V	L	W	H	-911.135
**'0101030105010101010104**	V	D	M	R	V	V	L	V	V	W	I	-911.131
**'0101020205010101040104**	V	D	L	H	V	V	L	V	L	W	I	-911.131
**'0101040105010105010110**	V	D	V	R	V	V	L	M	V	W	R	-911.13
**'0101010202020104010106**	V	D	I	H	F	I	L	L	V	W	L	-911.128
**'0101010405020101070104**	V	D	I	Q	V	I	L	V	T	W	I	-911.124
**'0301010102020101010110**	L	D	I	R	F	I	L	V	V	W	R	-911.111
**'0101030102020102010104**	V	D	M	R	F	I	L	A	V	W	I	-911.109
**'0101030205010103010103**	V	D	M	H	V	V	L	I	V	W	F	-911.108
**'0201010103030101030105**	I	D	I	R	I	L	L	V	I	W	K	-911.106
**'0101010205020101010106**	V	D	I	H	V	I	L	V	V	W	L	-911.104
**'0101030103010101050104**	V	D	M	R	I	V	L	V	M	W	I	-911.1
**'0101030303020101010105**	V	D	M	K	I	I	L	V	V	W	K	-911.094
**'0101010103010103010106**	V	D	I	R	I	V	L	I	V	W	L	-911.089
**'0101040102020203010101**	V	D	V	R	F	I	A	I	V	W	H	-911.087
**'0101030102020201040101**	V	D	M	R	F	I	A	V	L	W	H	-911.086
**'0201010105010105040104**	I	D	I	R	V	V	L	M	L	W	I	-911.08
**'0201040102030101010108**	I	D	V	R	F	L	L	V	V	W	N	-911.073
**'0101030201020103010112**	V	D	M	H	L	I	L	I	V	W	V	-911.072
**'0101020205010103010101**	V	D	L	H	V	V	L	I	V	W	H	-911.066
**'0101030205010201040101**	V	D	M	H	V	V	A	V	L	W	H	-911.064
**'0101010102030103010103**	V	D	I	R	F	L	L	I	V	W	F	-911.057
**'0101040102030101020102**	V	D	V	R	F	L	L	V	A	W	E	-911.046
**'0101020102020101050102**	V	D	L	R	F	I	L	V	M	W	E	-911.045
**'0101020203020101020101**	V	D	L	H	I	I	L	V	A	W	H	-911.044
**'0301010102020101020105**	L	D	I	R	F	I	L	V	A	W	K	-911.041
**'0101040102010103070106**	V	D	V	R	F	V	L	I	T	W	L	-911.04
**'0101010402010201040106**	V	D	I	Q	F	V	A	V	L	W	L	-911.039
**'0101010102030203010108**	V	D	I	R	F	L	A	I	V	W	N	-911.034

'0301010102020101040108	L	D	I	R	F	I	L	V	L	W	N	-911.033
'0101010304010103040101	V	D	I	K	M	V	L	I	L	W	H	-911.026
'0201010201030103010112	I	D	I	H	L	L	L	I	V	W	V	-911.026
'0101040104030101070110	V	D	V	R	M	L	L	V	T	W	R	-911.024
'0201010101030101040106	I	D	I	R	L	L	L	V	L	W	L	-911.024
'0101010202030101050103	V	D	I	H	F	L	L	V	M	W	F	-911.019
'0201020102020101010105	I	D	L	R	F	I	L	V	V	W	K	-911.019
'0201010103030101020111	I	D	I	R	I	L	L	V	A	W	T	-911.019
'0101040302010101020106	V	D	V	K	F	V	L	V	A	W	L	-911.011
'0101040102030101070103	V	D	V	R	F	L	L	V	T	W	F	-911.01
'0101010302040101020105	V	D	I	K	F	M	L	V	A	W	K	-911.007
'0101010103030103010108	V	D	I	R	I	L	L	I	V	W	N	-911.006
'0101010302020101040106	V	D	I	K	F	I	L	V	L	W	L	-911.005
'0101010302010103030112	V	D	I	K	F	V	L	I	I	W	V	-911.005
'0101040104020104010105	V	D	V	R	M	I	L	L	V	W	K	-910.999
'0101030103020103040101	V	D	M	R	I	I	L	I	L	W	H	-910.987
'0101010205020203010101	V	D	I	H	V	I	A	I	V	W	H	-910.975
'0101010205020101050110	V	D	I	H	V	I	L	V	M	W	R	-910.973
'0101030405010103070101	V	D	M	Q	V	V	L	I	T	W	H	-910.968
'0101010105020203040101	V	D	I	R	V	I	A	I	L	W	H	-910.968
'0201030103010101040104	I	D	M	R	I	V	L	V	L	W	I	-910.968
'0101010202010104040110	V	D	I	H	F	V	L	L	L	W	R	-910.964
'0101040403030101010104	V	D	V	Q	I	L	L	V	V	W	I	-910.953
'0101010103020201040106	V	D	I	R	I	I	A	V	L	W	L	-910.951
'0101010203040101040109	V	D	I	H	I	M	L	V	L	W	Q	-910.948
'0101010202010103020108	V	D	I	H	F	V	L	I	A	W	N	-910.943
'0101010102030101070112	V	D	I	R	F	L	L	V	T	W	V	-910.94
'0101040105010104040109	V	D	V	R	V	V	L	L	L	W	Q	-910.926
'0201010102010103040104	I	D	I	R	F	V	L	I	L	W	I	-910.911
'0101030102010101070112	V	D	M	R	F	V	L	V	T	W	V	-910.906
'0201010102020101030101	I	D	I	R	F	I	L	V	I	W	H	-910.905
'0101040102030301010112	V	D	V	R	F	L	V	V	V	W	V	-910.904
'0101010402010103070106	V	D	I	Q	F	V	L	I	T	W	L	-910.903
'0101040304020101010110	V	D	V	K	M	I	L	V	V	W	R	-910.902
'0101010402030201010105	V	D	I	Q	F	L	A	V	V	W	K	-910.898
'0101030102020101010111	V	D	M	R	F	I	L	V	V	W	T	-910.897
'0101030105040101070112	V	D	M	R	V	M	L	V	T	W	V	-910.897

**'0101030105010104010101**	V	D	M	R	V	V	L	L	V	W	H	-910.897
**'0101040103010101040104**	V	D	V	R	I	V	L	V	L	W	I	-910.894
**'0101010405010103030112**	V	D	I	Q	V	V	L	I	I	W	V	-910.887
**'0201040203030101010101**	I	D	V	H	I	L	L	V	V	W	H	-910.885
**'0101010302040101070109**	V	D	I	K	F	M	L	V	T	W	Q	-910.883
**'0101010405030101010111**	V	D	I	Q	V	L	L	V	V	W	T	-910.865
**'0101030105010203010101**	V	D	M	R	V	V	A	I	V	W	H	-910.861
**'0101010205020104010104**	V	D	I	H	V	I	L	L	V	W	I	-910.858
**'0101020202010101040110**	V	D	L	H	F	V	L	V	L	W	R	-910.845
**'0101010402010103010102**	V	D	I	Q	F	V	L	I	V	W	E	-910.842
**'0101030101030103040110**	V	D	M	R	L	L	L	I	L	W	R	-910.839
**'0101010102040101010112**	V	D	I	R	F	M	L	V	V	W	V	-910.832
**'0201010103010103020112**	I	D	I	R	I	V	L	I	A	W	V	-910.83
**'0101030302030101010112**	V	D	M	K	F	L	L	V	V	W	V	-910.829
**'0301040102010101010101**	L	D	V	R	F	V	L	V	V	W	H	-910.825
**'0101030402010103040101**	V	D	M	Q	F	V	L	I	L	W	H	-910.822
**'0301030103020101010104**	L	D	M	R	I	I	L	V	V	W	I	-910.809
**'0101010102040101040110**	V	D	I	R	F	M	L	V	L	W	R	-910.807
**'0101020102010104040106**	V	D	L	R	F	V	L	L	L	W	L	-910.801
**'0101030105010101040110**	V	D	M	R	V	V	L	V	L	W	R	-910.798
**'0101040101020103010105**	V	D	V	R	L	I	L	I	V	W	K	-910.798
**'0101010302010104010112**	V	D	I	K	F	V	L	L	V	W	V	-910.797
**'0101030102010104050101**	V	D	M	R	F	V	L	L	M	W	H	-910.784
**'0101020202030101010107**	V	D	L	H	F	L	L	V	V	W	M	-910.779
**'0101030103030101050106**	V	D	M	R	I	L	L	V	M	W	L	-910.772
**'0201040102020103010101**	I	D	V	R	F	I	L	I	V	W	H	-910.768
**'0101010305020101030108**	V	D	I	K	V	I	L	V	I	W	N	-910.764
**'0101010104030101040106**	V	D	I	R	M	L	L	V	L	W	L	-910.762
**'0101030105010101030112**	V	D	M	R	V	V	L	V	I	W	V	-910.758
**'0101030105040101040109**	V	D	M	R	V	M	L	V	L	W	Q	-910.751
**'0101010105020403010104**	V	D	I	R	V	I	W	I	V	W	I	-910.75
**'0101010303010103040108**	V	D	I	K	I	V	L	I	L	W	N	-910.738
**'0101010203030101030107**	V	D	I	H	I	L	L	V	I	W	M	-910.737
**'0101040202020101010103**	V	D	V	H	F	I	L	V	V	W	F	-910.736
**'0101030203040101010110**	V	D	M	H	I	M	L	V	V	W	R	-910.725
**'0201010103030101040109**	I	D	I	R	I	L	L	V	L	W	Q	-910.722
**'0101010202030104010110**	V	D	I	H	F	L	L	L	V	W	R	-910.72

'0201010105030101070109	I	D	I	R	V	L	L	V	T	W	Q	-910.717
'0101010402010104040106	V	D	I	Q	F	V	L	L	L	W	L	-910.712
'0101030102020101060110	V	D	M	R	F	I	L	V	S	W	R	-910.701
'0101030103030103010105	V	D	M	R	I	L	L	I	V	W	K	-910.697
'0101040103040101040106	V	D	V	R	I	M	L	V	L	W	L	-910.694
'0301030102020101010106	L	D	M	R	F	I	L	V	V	W	L	-910.692
'0101010202040103010112	V	D	I	H	F	M	L	I	V	W	V	-910.69
'0101010102010201010104	V	D	I	R	F	V	A	V	V	W	I	-910.688
'0101010402020103020101	V	D	I	Q	F	I	L	I	A	W	H	-910.687
'0101040105030301050101	V	D	V	R	V	L	V	V	M	W	H	-910.678
'0101020102010101040101	V	D	L	R	F	V	L	V	L	W	H	-910.677
'0101030301030101040106	V	D	M	K	L	L	L	V	L	W	L	-910.666
'0101010203040101030104	V	D	I	H	I	M	L	V	I	W	I	-910.664
'0101010102020103010110	V	D	I	R	F	I	L	I	V	W	R	-910.662
'0101010103010101040106	V	D	I	R	I	V	L	V	L	W	L	-910.662
'0101010204020101020106	V	D	I	H	M	I	L	V	A	W	L	-910.661
'0101030305010103040101	V	D	M	K	V	V	L	I	L	W	H	-910.657
'0101030202040101040101	V	D	M	H	F	M	L	V	L	W	H	-910.649
'0101010102010203030112	V	D	I	R	F	V	A	I	I	W	V	-910.643
'0201010101030103040105	I	D	I	R	L	L	L	I	L	W	K	-910.641
'0101010405020101050102	V	D	I	Q	V	I	L	V	M	W	E	-910.64
'0101030202010101050110	V	D	M	H	F	V	L	V	M	W	R	-910.64
'0101040102010101010107	V	D	V	R	F	V	L	V	V	W	M	-910.636
'0101010202010201040104	V	D	I	H	F	V	A	V	L	W	I	-910.636
'0201040102040102010101	I	D	V	R	F	M	L	A	V	W	H	-910.627
'0101020102010103040108	V	D	L	R	F	V	L	I	L	W	N	-910.626
'0101030303030101020101	V	D	M	K	I	L	L	V	A	W	H	-910.623
'0101010103010101030112	V	D	I	R	I	V	L	V	I	W	V	-910.621
'0101040203010104070101	V	D	V	H	I	V	L	L	T	W	H	-910.62
'0101040105020104020101	V	D	V	R	V	I	L	L	A	W	H	-910.614
'0101010402010101040105	V	D	I	Q	F	V	L	V	L	W	K	-910.613
'0301010405020101010104	L	D	I	Q	V	I	L	V	V	W	I	-910.605
'0301040202010101010106	L	D	V	H	F	V	L	V	V	W	L	-910.601
'0101010103030101070108	V	D	I	R	I	L	L	V	T	W	N	-910.599
'0101040102010101020109	V	D	V	R	F	V	L	V	A	W	Q	-910.596
'0201010102030101050109	I	D	I	R	F	L	L	V	M	W	Q	-910.589
'0301040105010104070101	L	D	V	R	V	V	L	L	T	W	H	-910.589

'0201030102010101020104	I	D	M	R	F	V	L	V	A	W	I	-910.588
'0101020202030101040101	V	D	L	H	F	L	L	V	L	W	H	-910.586
'0101030405030101010109	V	D	M	Q	V	L	L	V	V	W	Q	-910.58
'0101010102020104010109	V	D	I	R	F	I	L	L	V	W	Q	-910.574
'0201040105010101020108	I	D	V	R	V	V	L	V	A	W	N	-910.569
'0101020102040101010105	V	D	L	R	F	M	L	V	V	W	K	-910.565
'0101010204040101040110	V	D	I	H	M	M	L	V	L	W	R	-910.561
'0101010205020101040105	V	D	I	H	V	I	L	V	L	W	K	-910.56
'0101010204040101070104	V	D	I	H	M	M	L	V	T	W	I	-910.555
'0101010105020201020110	V	D	I	R	V	I	A	V	A	W	R	-910.555
'0101040103010303010105	V	D	V	R	I	V	V	I	V	W	K	-910.546
'0101010102010304010106	V	D	I	R	F	V	V	L	V	W	L	-910.541
'0301010202010104010101	L	D	I	H	F	V	L	L	V	W	H	-910.539
'0101040202010204010101	V	D	V	H	F	V	A	L	V	W	H	-910.539
'0101030102010101030104	V	D	M	R	F	V	L	V	I	W	I	-910.537
'0101040204010104010109	V	D	V	H	M	V	L	L	V	W	Q	-910.533
'0101010303020101030106	V	D	I	K	I	I	L	V	I	W	L	-910.531
'0101010103030301070109	V	D	I	R	I	L	V	V	T	W	Q	-910.525
'0101010203020101020104	V	D	I	H	I	I	L	V	A	W	I	-910.524
'0301010103020102010112	L	D	I	R	I	I	L	A	V	W	V	-910.521
'0301010403020101010106	L	D	I	Q	I	I	L	V	V	W	L	-910.5
'0101020302030101070101	V	D	L	K	F	L	L	V	T	W	H	-910.496
'0201010102040101070112	I	D	I	R	F	M	L	V	T	W	V	-910.489
'0101010403010103030109	V	D	I	Q	I	V	L	I	I	W	Q	-910.488
'0201010102020201010109	I	D	I	R	F	I	A	V	V	W	Q	-910.486
'0101020104030101040104	V	D	L	R	M	L	L	V	L	W	I	-910.484
'0101030202010101070105	V	D	M	H	F	V	L	V	T	W	K	-910.477
'0101040104010105040106	V	D	V	R	M	V	L	M	L	W	L	-910.476
'0101010102010103060105	V	D	I	R	F	V	L	I	S	W	K	-910.476
'0301010105020104010106	L	D	I	R	V	I	L	L	V	W	L	-910.474
'0101030202010104010102	V	D	M	H	F	V	L	L	V	W	E	-910.473
'0101040105020101060111	V	D	V	R	V	I	L	V	S	W	T	-910.463
'0101040402020101020101	V	D	V	Q	F	I	L	V	A	W	H	-910.456
'0101030105020201020101	V	D	M	R	V	I	A	V	A	W	H	-910.456
'0101030102020201010105	V	D	M	R	F	I	A	V	V	W	K	-910.453
'0101040103010104040105	V	D	V	R	I	V	L	L	L	W	K	-910.452
'0201010201010103040104	I	D	I	H	L	V	L	I	L	W	I	-910.452

'010103020202020104010101	V	D	M	H	F	I	L	L	V	W	H	-910.45
'010101010503030104010 4	V	D	I	R	V	L	V	V	L	W	I	-910.448
'010104010201040301011 2	V	D	V	R	F	V	W	I	V	W	V	-910.447
'010102040201010301010 6	V	D	L	Q	F	V	L	I	V	W	L	-910.447
'010104030503010101010 3	V	D	V	K	V	L	L	V	V	W	F	-910.442
'020101010203010103010 2	I	D	I	R	F	L	L	V	I	W	E	-910.439
'020103010303010101010 6	I	D	M	R	I	L	L	V	V	W	L	-910.436
'010101010203030104011 0	V	D	I	R	F	L	V	V	L	W	R	-910.435
'010101010302040104010 4	V	D	I	R	I	I	W	V	L	W	I	-910.435
'010104010504010301010 4	V	D	V	R	V	M	L	I	V	W	I	-910.434
'020101010501010501010 6	I	D	I	R	V	V	L	M	V	W	L	-910.432
'010101010501010404010 7	V	D	I	R	V	V	L	L	L	W	M	-910.432
'010102010302010104010 8	V	D	L	R	I	I	L	V	L	W	N	-910.427
'010104040103010104011 2	V	D	V	Q	L	L	L	V	L	W	V	-910.423
'020104010504010101011 0	I	D	V	R	V	M	L	V	V	W	R	-910.423
'030104010202010104010 1	L	D	V	R	F	I	L	V	L	W	H	-910.422
'010104020301010105010 9	V	D	V	H	I	V	L	V	M	W	Q	-910.421
'010101020201020107011 2	V	D	I	H	F	V	A	V	T	W	V	-910.417
'010104010302010501010 4	V	D	V	R	I	I	L	M	V	W	I	-910.412
'010104020201010101010 8	V	D	V	H	F	V	L	V	V	W	N	-910.41
'020101010203010207010 1	I	D	I	R	F	L	L	A	T	W	H	-910.41
'010101020201010504010 5	V	D	I	H	F	V	L	M	L	W	K	-910.405
'010101030203020101011 0	V	D	I	K	F	L	A	V	V	W	R	-910.404
'010101010202020103011 0	V	D	I	R	F	I	A	V	I	W	R	-910.399
'010101010502020103010 8	V	D	I	R	V	I	A	V	I	W	N	-910.397
'010103030201010104010 1	V	D	M	K	F	V	L	V	L	W	H	-910.396
'010102010302010301011 0	V	D	L	R	I	I	L	I	V	W	R	-910.395
'020101010401010307010 6	I	D	I	R	M	V	L	I	T	W	L	-910.392
'020103010501010107010 6	I	D	M	R	V	V	L	V	T	W	L	-910.388
'020104010502010201010 1	I	D	V	R	V	I	L	A	V	W	H	-910.388
'010103030301010104010 6	V	D	M	K	I	V	L	V	L	W	L	-910.386
'010104020201010301011 1	V	D	V	H	F	V	L	I	V	W	T	-910.383
'010101030402010104010 1	V	D	I	K	M	I	L	V	L	W	H	-910.381
'020101020203010105010 1	I	D	I	H	F	L	L	V	M	W	H	-910.38
'010103010203020102010 1	V	D	M	R	F	L	A	V	A	W	H	-910.378
'010104040502010101010 4	V	D	V	Q	V	I	L	V	V	W	I	-910.377
'030101010502010102011 2	L	D	I	R	V	I	L	V	A	W	V	-910.376

**'0101010102010201040101**	V	D	I	R	F	V	A	V	L	W	H	-910.371
**'0101020103010103040106**	V	D	L	R	I	V	L	I	L	W	L	-910.369
**'0101010302020101020103**	V	D	I	K	F	I	L	V	A	W	F	-910.367
**'0301030202020101010101**	L	D	M	H	F	I	L	V	V	W	H	-910.365
**'0301010105010101010104**	L	D	I	R	V	V	L	V	V	W	I	-910.358
**'0101030302020101060101**	V	D	M	K	F	I	L	V	S	W	H	-910.355
**'0101020105020103050101**	V	D	L	R	V	I	L	I	M	W	H	-910.355
**'0101040403010103010106**	V	D	V	Q	I	V	L	I	V	W	L	-910.354
**'0101010305020104010110**	V	D	I	K	V	I	L	L	V	W	R	-910.352
**'0101030305010104010110**	V	D	M	K	V	V	L	L	V	W	R	-910.348
**'0101040305020101040101**	V	D	V	K	V	I	L	V	L	W	H	-910.343
**'0101040105030401010101**	V	D	V	R	V	L	W	V	V	W	H	-910.334
**'0101010102020203010104**	V	D	I	R	F	I	A	I	V	W	I	-910.332
**'0201010105030103050101**	I	D	I	R	V	L	L	I	M	W	H	-910.332
**'0101030102010104070110**	V	D	M	R	F	V	L	L	T	W	R	-910.331
**'0201040402040101010101**	I	D	V	Q	F	M	L	V	V	W	H	-910.33
**'0201010105040101010104**	I	D	I	R	V	M	L	V	V	W	I	-910.33
**'0101030103040101040111**	V	D	M	R	I	M	L	V	L	W	T	-910.329
**'0201040103010101070101**	I	D	V	R	I	V	L	V	T	W	H	-910.328
**'0101030302040101010108**	V	D	M	K	F	M	L	V	V	W	N	-910.327
**'0201020102030103010101**	I	D	L	R	F	L	L	I	V	W	H	-910.327
**'0101040203010104010107**	V	D	V	H	I	V	L	L	V	W	M	-910.325
**'0101040202040101010101**	V	D	V	H	F	M	L	V	V	W	H	-910.324
**'0101040105010301070104**	V	D	V	R	V	V	V	V	T	W	I	-910.322
**'0101010202020101030105**	V	D	I	H	F	I	L	V	I	W	K	-910.321
**'0101040405010101040107**	V	D	V	Q	V	V	L	V	L	W	M	-910.321
**'0101010105010104020101**	V	D	I	R	V	V	L	L	A	W	H	-910.319
**'0201010105020102010103**	I	D	I	R	V	I	L	A	V	W	F	-910.317
**'0101010102020201010103**	V	D	I	R	F	I	A	V	V	W	F	-910.317
**'0101030203010104010104**	V	D	M	H	I	V	L	L	V	W	I	-910.311
**'0201040202010101010104**	I	D	V	H	F	V	L	V	V	W	I	-910.311
**'0101040103020101020112**	V	D	V	R	I	I	L	V	A	W	V	-910.296
**'0101010105020303010108**	V	D	I	R	V	I	V	I	V	W	N	-910.293
**'0101040403020101010108**	V	D	V	Q	I	I	L	V	V	W	N	-910.293
**'0101010202010103010101**	V	D	I	H	F	V	L	I	V	W	H	-910.288
**'0201010205010101010105**	I	D	I	H	V	V	L	V	V	W	K	-910.287
**'0101010102030401010110**	V	D	I	R	F	L	W	V	V	W	R	-910.284

**'0201010402030102010101**	I	D	I	Q	F	L	L	A	V	W	H	-910.284
**'0101010405020101060111**	V	D	I	Q	V	I	L	V	S	W	T	-910.282
**'0101030402020201010101**	V	D	M	Q	F	I	A	V	V	W	H	-910.281
**'0101030202010103060101**	V	D	M	H	F	V	L	I	S	W	H	-910.28
**'0101030305020101010107**	V	D	M	K	V	I	L	V	V	W	M	-910.27
**'0101040102030101060110**	V	D	V	R	F	L	L	V	S	W	R	-910.27
**'0101030102020104010105**	V	D	M	R	F	I	L	L	V	W	K	-910.266
**'0101010405030101050104**	V	D	I	Q	V	L	L	V	M	W	I	-910.258
**'0201040103020101010105**	I	D	V	R	I	I	L	V	V	W	K	-910.256
**'0201010105020101020107**	I	D	I	R	V	I	L	V	A	W	M	-910.251
**'0101030102020401010101**	V	D	M	R	F	I	W	V	V	W	H	-910.25
**'0101030205010101050109**	V	D	M	H	V	V	L	V	M	W	Q	-910.248
**'0101030305030101010104**	V	D	M	K	V	L	L	V	V	W	I	-910.243
**'0101030201020101030104**	V	D	M	H	L	I	L	V	I	W	I	-910.242
**'0101020203010101040106**	V	D	L	H	I	V	L	V	L	W	L	-910.242
**'0101010203030101010109**	V	D	I	H	I	L	L	V	V	W	Q	-910.242
**'0101030205010101030108**	V	D	M	H	V	V	L	V	I	W	N	-910.241
**'0101040103030104010106**	V	D	V	R	I	L	L	L	V	W	L	-910.236
**'0101010102030104040102**	V	D	I	R	F	L	L	L	L	W	E	-910.234
**'0101010403030101040108**	V	D	I	Q	I	L	L	V	L	W	N	-910.234
**'0101020302020101040101**	V	D	L	K	F	I	L	V	L	W	H	-910.234
**'0101040103030103010104**	V	D	V	R	I	L	L	I	V	W	I	-910.228
**'0101040105030103050101**	V	D	V	R	V	L	L	I	M	W	H	-910.227
**'0101010105010104030105**	V	D	I	R	V	V	L	L	I	W	K	-910.227
**'0101020102010103020110**	V	D	L	R	F	V	L	I	A	W	R	-910.227
**'0101010202040101050105**	V	D	I	H	F	M	L	V	M	W	K	-910.225
**'0201040104010105010104**	I	D	V	R	M	V	L	M	V	W	I	-910.224
**'0101010302020201010106**	V	D	I	K	F	I	A	V	V	W	L	-910.22
**'0101010102010101020106**	V	D	I	R	F	V	L	V	A	W	L	-910.218
**'0101030102010301040104**	V	D	M	R	F	V	V	V	L	W	I	-910.216
**'0201030105010101010101**	I	D	M	R	V	V	L	V	V	W	H	-910.215
**'0201010302010103010109**	I	D	I	K	F	V	L	I	V	W	Q	-910.214
**'0201040102040101070101**	I	D	V	R	F	M	L	V	T	W	H	-910.206
**'0101040202010101020103**	V	D	V	H	F	V	L	V	A	W	F	-910.205
**'0101040102010301010105**	V	D	V	R	F	V	V	V	V	W	K	-910.203
**'0101040102010101050108**	V	D	V	R	F	V	L	V	M	W	N	-910.199
**'0101010302020103030101**	V	D	I	K	F	I	L	I	I	W	H	-910.196

'0101010105010101030108	V	D	I	R	V	V	L	V	I	W	N	-910.195
'0101040203030401010101	V	D	V	H	I	L	W	V	V	W	H	-910.193
'0101030405040101010104	V	D	M	Q	V	M	L	V	V	W	I	-910.185
'0101010203020401010106	V	D	I	H	I	I	W	V	V	W	L	-910.182
'0101030303020101040101	V	D	M	K	I	I	L	V	L	W	H	-910.179
'0101040102010104030110	V	D	V	R	F	V	L	L	I	W	R	-910.176
'0101010103010103020108	V	D	I	R	I	V	L	I	A	W	N	-910.172
'0101010203030201020101	V	D	I	H	I	L	A	V	A	W	H	-910.17
'0101040205010104010108	V	D	V	H	V	V	L	L	V	W	N	-910.168
'0101030103010104070112	V	D	M	R	I	V	L	L	T	W	V	-910.163
'0101040202010105010101	V	D	V	H	F	V	L	M	V	W	H	-910.161
'0201010103030101070106	I	D	I	R	I	L	L	V	T	W	L	-910.161
'0101020402030101010106	V	D	L	Q	F	L	L	V	V	W	L	-910.16
'0101010203010105020105	V	D	I	H	I	V	L	M	A	W	K	-910.157
'0101030405010101040101	V	D	M	Q	V	V	L	V	L	W	H	-910.154
'0101010305010101030112	V	D	I	K	V	V	L	V	I	W	V	-910.15
'0101010105010201040102	V	D	I	R	V	V	A	V	L	W	E	-910.144
'0101040102010103060102	V	D	V	R	F	V	L	I	S	W	E	-910.136
'0101040105020101030112	V	D	V	R	V	I	L	V	I	W	V	-910.134
'0101010202030401010101	V	D	I	H	F	L	W	V	V	W	H	-910.134
'0101010202010403010108	V	D	I	H	F	V	W	I	V	W	N	-910.131
'0101010204030103040101	V	D	I	H	M	L	L	I	L	W	H	-910.13
'0101030103030101070104	V	D	M	R	I	L	L	V	T	W	I	-910.121
'0301040105010101010112	L	D	V	R	V	V	L	V	V	W	V	-910.115
'0101010102040201070106	V	D	I	R	F	M	A	V	T	W	L	-910.113
'0101030103010103050101	V	D	M	R	I	V	L	I	M	W	H	-910.109
'0101010103020104040101	V	D	I	R	I	I	L	L	L	W	H	-910.107
'0201030105020101070101	I	D	M	R	V	I	L	V	T	W	H	-910.105
'0101010102010201020111	V	D	I	R	F	V	A	V	A	W	T	-910.105
'0101030203020301010101	V	D	M	H	I	I	V	V	V	W	H	-910.103
'0301040102010104010108	L	D	V	R	F	V	L	L	V	W	N	-910.103
'0101040105020105010105	V	D	V	R	V	I	L	M	V	W	K	-910.097
'0101010103030301040112	V	D	I	R	I	L	V	V	L	W	V	-910.094
'0101020103030101010108	V	D	L	R	I	L	L	V	V	W	N	-910.088
'0101030203010101040111	V	D	M	H	I	V	L	V	L	W	T	-910.085
'0101010103020401010112	V	D	I	R	I	I	W	V	V	W	V	-910.085
'0101040302010103060101	V	D	V	K	F	V	L	I	S	W	H	-910.081

'0101030105010201040112	V	D	M	R	V	V	A	V	L	W	V	-910.081
'0101010302030103040101	V	D	I	K	F	L	L	I	L	W	H	-910.077
'0101020202040101010112	V	D	L	H	F	M	L	V	V	W	V	-910.076
'0201010402010103010112	I	D	I	Q	F	V	L	I	V	W	V	-910.076
'0101020102020201010109	V	D	L	R	F	I	A	V	V	W	Q	-910.074
'0201030105010103010102	I	D	M	R	V	V	L	I	V	W	E	-910.073
'0101010103010104040104	V	D	I	R	I	V	L	L	L	W	I	-910.071
'0201040104020105010101	I	D	V	R	M	I	L	M	V	W	H	-910.071
'0201010402040101010105	I	D	I	Q	F	M	L	V	V	W	K	-910.063
'0101040105030101060105	V	D	V	R	V	L	L	V	S	W	K	-910.06
'0101030103020401010108	V	D	M	R	I	I	W	V	V	W	N	-910.05
'0101020302020103010101	V	D	L	K	F	I	L	I	V	W	H	-910.049
'0201040105010103010109	I	D	V	R	V	V	L	I	V	W	Q	-910.046
'0201040105040101020101	I	D	V	R	V	M	L	V	A	W	H	-910.046
'0201040103010101040105	I	D	V	R	I	V	L	V	L	W	K	-910.045
'0201010102020101010107	I	D	I	R	F	I	L	V	V	W	M	-910.044
'0101010102010204040110	V	D	I	R	F	V	A	L	L	W	R	-910.042
'0101030405020101050101	V	D	M	Q	V	I	L	V	M	W	H	-910.04
'0101040304020101020101	V	D	V	K	M	I	L	V	A	W	H	-910.04
'0101010105010105040106	V	D	I	R	V	V	L	M	L	W	L	-910.039
'0101030102010303010112	V	D	M	R	F	V	V	I	V	W	V	-910.03
'0101010202010104070107	V	D	I	H	F	V	L	L	T	W	M	-910.022
'0101010102020301020109	V	D	I	R	F	I	V	V	A	W	Q	-910.021
'0101010202030301040101	V	D	I	H	F	L	V	V	L	W	H	-910.018
'0101040403010101040106	V	D	V	Q	I	V	L	V	L	W	L	-910.011
'0101010102020201020106	V	D	I	R	F	I	A	V	A	W	L	-910.009
'0101030103030201010104	V	D	M	R	I	L	A	V	V	W	I	-909.999
'0101010302010103010103	V	D	I	K	F	V	L	I	V	W	F	-909.998
'0101010202020401010105	V	D	I	H	F	I	W	V	V	W	K	-909.997
'0101040102020102010106	V	D	V	R	F	I	L	A	V	W	L	-909.996
'0301030102020101070101	L	D	M	R	F	I	L	V	T	W	H	-909.986
'0301020105020103010101	L	D	L	R	V	I	L	I	V	W	H	-909.985
'0101010402020104010101	V	D	I	Q	F	I	L	L	V	W	H	-909.975
'0101030102020301010104	V	D	M	R	F	I	V	V	V	W	I	-909.974
'0101010402030101010110	V	D	I	Q	F	L	L	V	V	W	R	-909.973
'0101010303040101040108	V	D	I	K	I	M	L	V	L	W	N	-909.971
'0101010102040301040106	V	D	I	R	F	M	V	V	L	W	L	-909.968

'0101010202040201010106	V	D	I	H	F	M	A	V	V	W	L	-909.967
'0101010102020301010112	V	D	I	R	F	I	V	V	V	W	V	-909.966
'0301040102020104010101	L	D	V	R	F	I	L	L	V	W	H	-909.965
'0101010103020301070104	V	D	I	R	I	I	V	V	T	W	I	-909.964
'0101010105030301070105	V	D	I	R	V	L	V	V	T	W	K	-909.963
'0101010203030101070105	V	D	I	H	I	L	L	V	T	W	K	-909.962
'0101010105010204010106	V	D	I	R	V	V	A	L	V	W	L	-909.957
'0201010105010103030112	I	D	I	R	V	V	L	I	I	W	V	-909.956
'0101040202010101070106	V	D	V	H	F	V	L	V	T	W	L	-909.955
'0201010102010105020101	I	D	I	R	F	V	L	M	A	W	H	-909.954
'0101030105030101020103	V	D	M	R	V	L	L	V	A	W	F	-909.951
'0101030102030201010107	V	D	M	R	F	L	A	V	V	W	M	-909.951
'0101040202010403010101	V	D	V	H	F	V	W	I	V	W	H	-909.948
'0101010103030301020105	V	D	I	R	I	L	V	V	A	W	K	-909.946
'0101040105010204010104	V	D	V	R	V	V	A	L	V	W	I	-909.937
'0201030103020101040101	I	D	M	R	I	I	L	V	L	W	H	-909.937
'0101040203010101010102	V	D	V	H	I	V	L	V	V	W	E	-909.935
'0301040103010104010101	L	D	V	R	I	V	L	L	V	W	H	-909.933
'0301010105010101040105	L	D	I	R	V	V	L	V	L	W	K	-909.927
'0101040104020101040104	V	D	V	R	M	I	L	V	L	W	I	-909.92
'0101010102030201020107	V	D	I	R	F	L	A	V	A	W	M	-909.919
'0201010405030101010109	I	D	I	Q	V	L	L	V	V	W	Q	-909.915
'0101040105040101010112	V	D	V	R	V	M	L	V	V	W	V	-909.913
'0101030201030101030101	V	D	M	H	L	L	L	V	I	W	H	-909.912
'0201010202010105010106	I	D	I	H	F	V	L	M	V	W	L	-909.912
'0301040102010101020106	L	D	V	R	F	V	L	V	A	W	L	-909.909
'0101030105030101010106	V	D	M	R	V	L	L	V	V	W	L	-909.908
'0101010102010103070110	V	D	I	R	F	V	L	I	T	W	R	-909.9
'0101040105020204010101	V	D	V	R	V	I	A	L	V	W	H	-909.899
'0101010102010203050105	V	D	I	R	F	V	A	I	M	W	K	-909.899
'0101030102010104020112	V	D	M	R	F	V	L	L	A	W	V	-909.897
'0101040205010103070101	V	D	V	H	V	V	L	I	T	W	H	-909.89
'0101010402010104070112	V	D	I	Q	F	V	L	L	T	W	V	-909.889
'0101040203040101010105	V	D	V	H	I	M	L	V	V	W	K	-909.884
'0101040303010103040101	V	D	V	K	I	V	L	I	L	W	H	-909.884
'0201010103030301010112	I	D	I	R	I	L	V	V	V	W	V	-909.884
'0101030105010101020102	V	D	M	R	V	V	L	V	A	W	E	-909.884

**'0101010102030401020108**	V	D	I	R	F	L	W	V	A	W	N	-909.882
**'0101030205040101020101**	V	D	M	H	V	M	L	V	A	W	H	-909.881
**'0101030302010101020105**	V	D	M	K	F	V	L	V	A	W	K	-909.878
**'0101040105020101050109**	V	D	V	R	V	I	L	V	M	W	Q	-909.876
**'0101010202010104060106**	V	D	I	H	F	V	L	L	S	W	L	-909.869
**'0101010205010403010105**	V	D	I	H	V	V	W	I	V	W	K	-909.867
**'0101020202010101070109**	V	D	L	H	F	V	L	V	T	W	Q	-909.866
**'0101040102020101040103**	V	D	V	R	F	I	L	V	L	W	F	-909.866
**'0101040105010101060108**	V	D	V	R	V	V	L	V	S	W	N	-909.854
**'0101020102030103010106**	V	D	L	R	F	L	L	I	V	W	L	-909.852
**'0101040103030101070101**	V	D	V	R	I	L	L	V	T	W	H	-909.851
**'0201010102010103030108**	I	D	I	R	F	V	L	I	I	W	N	-909.85
**'0101010202040301010105**	V	D	I	H	F	M	V	V	V	W	K	-909.85
**'0101040102030401010109**	V	D	V	R	F	L	W	V	V	W	Q	-909.845
**'0101010205020301010104**	V	D	I	H	V	I	V	V	V	W	I	-909.844
**'0101010102010104030109**	V	D	I	R	F	V	L	L	I	W	Q	-909.844
**'0101030103020101050101**	V	D	M	R	I	I	L	V	M	W	H	-909.842
**'0101020202010101030105**	V	D	L	H	F	V	L	V	I	W	K	-909.834
**'0101010205040201070101**	V	D	I	H	V	M	A	V	T	W	H	-909.832
**'0101010102040201040108**	V	D	I	R	F	M	A	V	L	W	N	-909.832
**'0201030102010103040101**	I	D	M	R	F	V	L	I	L	W	H	-909.831
**'0101040101030103040112**	V	D	V	R	L	L	L	I	L	W	V	-909.831
**'0301030105010101040104**	L	D	M	R	V	V	L	V	L	W	I	-909.83
**'0201010102030303010101**	I	D	I	R	F	L	V	I	V	W	H	-909.828
**'0301010105040101040106**	L	D	I	R	V	M	L	V	L	W	L	-909.828
**'0201010102020201040101**	I	D	I	R	F	I	A	V	L	W	H	-909.827
**'0101030101020101010106**	V	D	M	R	L	I	L	V	V	W	L	-909.826
**'0101010205010203010106**	V	D	I	H	V	V	A	I	V	W	L	-909.822
**'0101010204030101020104**	V	D	I	H	M	L	L	V	A	W	I	-909.819
**'0101040102010104050112**	V	D	V	R	F	V	L	L	M	W	V	-909.818
**'0101010102040101030108**	V	D	I	R	F	M	L	V	I	W	N	-909.814
**'0101030102040101050108**	V	D	M	R	F	M	L	V	M	W	N	-909.812
**'0101010102040103070106**	V	D	I	R	F	M	L	I	T	W	L	-909.808
**'0201010305010103010112**	I	D	I	K	V	V	L	I	V	W	V	-909.806
**'0101030305030101040101**	V	D	M	K	V	L	L	V	L	W	H	-909.805
**'0101010402030101030103**	V	D	I	Q	F	L	L	V	I	W	F	-909.803
**'0101010105020401020101**	V	D	I	R	V	I	W	V	A	W	H	-909.8

**'0201010105020103010101**	I	D	I	R	V	I	L	I	V	W	H	-909.8
**'0101020103030101020112**	V	D	L	R	I	L	L	V	A	W	V	-909.796
**'0201030104030103010101**	I	D	M	R	M	L	L	I	V	W	H	-909.787
**'0201030102010101010112**	I	D	M	R	F	V	L	V	V	W	V	-909.786
**'0101040203010101030106**	V	D	V	H	I	V	L	V	I	W	L	-909.785
**'0101030202010101020104**	V	D	M	H	F	V	L	V	A	W	I	-909.784
**'0201010105020101030104**	I	D	I	R	V	I	L	V	I	W	I	-909.776
**'0101030102020102040101**	V	D	M	R	F	I	L	A	L	W	H	-909.775
**'0201010103030101010108**	I	D	I	R	I	L	L	V	V	W	N	-909.773
**'0101020102010101070111**	V	D	L	R	F	V	L	V	T	W	T	-909.771
**'0101030202010201020101**	V	D	M	H	F	V	A	V	A	W	H	-909.77
**'0101040105010104030106**	V	D	V	R	V	V	L	L	I	W	L	-909.769
**'0101010402010101070108**	V	D	I	Q	F	V	L	V	T	W	N	-909.768
**'0101030405010104010104**	V	D	M	Q	V	V	L	L	V	W	I	-909.767
**'0101040103010101020101**	V	D	V	R	I	V	L	V	A	W	H	-909.766
**'0101030104010103040101**	V	D	M	R	M	V	L	I	L	W	H	-909.765
**'0101030201040101040105**	V	D	M	H	L	M	L	V	L	W	K	-909.764
**'0101030303010103010105**	V	D	M	K	I	V	L	I	V	W	K	-909.762
**'0101040103010201040101**	V	D	V	R	I	V	A	V	L	W	H	-909.758
**'0101040105010105040105**	V	D	V	R	V	V	L	M	L	W	K	-909.758
**'0101040101010103040106**	V	D	V	R	L	V	L	I	L	W	L	-909.757
**'0101010105020204010110**	V	D	I	R	V	I	A	L	V	W	R	-909.756
**'0101040101030201040107**	V	D	V	R	L	L	A	V	L	W	M	-909.755
**'0101010403020101020105**	V	D	I	Q	I	I	L	V	A	W	K	-909.75
**'0101010302030101030112**	V	D	I	K	F	L	L	V	I	W	V	-909.744
**'0101040103010103010102**	V	D	V	R	I	V	L	I	V	W	E	-909.741
**'0101010102020201060105**	V	D	I	R	F	I	A	V	S	W	K	-909.74
**'0201010204040101010109**	I	D	I	H	M	M	L	V	V	W	Q	-909.736
**'0101030103010101010108**	V	D	M	R	I	V	L	V	V	W	N	-909.735
**'0101040201030201040101**	V	D	V	H	L	L	A	V	L	W	H	-909.729
**'0101010103010103040102**	V	D	I	R	I	V	L	I	L	W	E	-909.727
**'0101010203020403010101**	V	D	I	H	I	I	W	I	V	W	H	-909.724
**'0101010402020201010104**	V	D	I	Q	F	I	A	V	V	W	I	-909.719
**'0101010102030203060101**	V	D	I	R	F	L	A	I	S	W	H	-909.716
**'0201030405020101010101**	I	D	M	Q	V	I	L	V	V	W	H	-909.715
**'0201040103020101050101**	I	D	V	R	I	I	L	V	M	W	H	-909.71
**'0101040205010104040101**	V	D	V	H	V	V	L	L	L	W	H	-909.707

**'0101040102010105040108**	V	D	V	R	F	V	L	M	L	W	N	-909.707
**'0101020103020101020109**	V	D	L	R	I	I	L	V	A	W	Q	-909.707
**'0101030102010103050103**	V	D	M	R	F	V	L	I	M	W	F	-909.706
**'0101030305010101040104**	V	D	M	K	V	V	L	V	L	W	I	-909.704
**'0101020303020101010101**	V	D	L	K	I	I	L	V	V	W	H	-909.7
**'0101010105020102030105**	V	D	I	R	V	I	L	A	I	W	K	-909.699
**'0201020102030101070101**	I	D	L	R	F	L	L	V	T	W	H	-909.698
**'0201010102010102040106**	I	D	I	R	F	V	L	A	L	W	L	-909.696
**'0101030201030201010106**	V	D	M	H	L	L	A	V	V	W	L	-909.695
**'0201010105010101010111**	I	D	I	R	V	V	L	V	V	W	T	-909.694
**'0101010305020102010104**	V	D	I	K	V	I	L	A	V	W	I	-909.692
**'0101030203010103020101**	V	D	M	H	I	V	L	I	A	W	H	-909.69
**'0101040103020104010112**	V	D	V	R	I	I	L	L	V	W	V	-909.69
**'0101010102020403020101**	V	D	I	R	F	I	W	I	A	W	H	-909.687
**'0101010105030201040108**	V	D	I	R	V	L	A	V	L	W	N	-909.685
**'0101030102010201070104**	V	D	M	R	F	V	A	V	T	W	I	-909.685
**'0101040405020101070101**	V	D	V	Q	V	I	L	V	T	W	H	-909.677
**'0101010102040301010101**	V	D	I	R	F	M	V	V	V	W	H	-909.675
**'0301010105040101020104**	L	D	I	R	V	M	L	V	A	W	I	-909.671
**'0101030301020101010104**	V	D	M	K	L	I	L	V	V	W	I	-909.67
**'0101040102030102040101**	V	D	V	R	F	L	L	A	L	W	H	-909.67
**'0301010105020101010108**	L	D	I	R	V	I	L	V	V	W	N	-909.666
**'0101020302040101010104**	V	D	L	K	F	M	L	V	V	W	I	-909.662
**'0101040103010103070101**	V	D	V	R	I	V	L	I	T	W	H	-909.662
**'0201020102010103010106**	I	D	L	R	F	V	L	I	V	W	L	-909.662
**'0101010205010104020105**	V	D	I	H	V	V	L	L	A	W	K	-909.66
**'0101030102010204010105**	V	D	M	R	F	V	A	L	V	W	K	-909.659
**'0101040104020104070101**	V	D	V	R	M	I	L	L	T	W	H	-909.659
**'0101030201010103040106**	V	D	M	H	L	V	L	I	L	W	L	-909.651
**'0101030101030101040109**	V	D	M	R	L	L	L	V	L	W	Q	-909.646
**'0301010105010104070104**	L	D	I	R	V	V	L	L	T	W	I	-909.644
**'0101010105020201070102**	V	D	I	R	V	I	A	V	T	W	E	-909.642
**'0201010104030103010106**	I	D	I	R	M	L	L	I	V	W	L	-909.641
**'0201030101030101040104**	I	D	M	R	L	L	L	V	L	W	I	-909.64
**'0101010104020101050104**	V	D	I	R	M	I	L	V	M	W	I	-909.638
**'0101030102010201040106**	V	D	M	R	F	V	A	V	L	W	L	-909.638
**'0101010104010103010105**	V	D	I	R	M	V	L	I	V	W	K	-909.637

**'0101040105010103050103**	V	D	V	R	V	V	L	I	M	W	F	-909.634
**'0101010402020101060106**	V	D	I	Q	F	I	L	V	S	W	L	-909.628
**'0101010202020201010109**	V	D	I	H	F	I	A	V	V	W	Q	-909.628
**'0101010202010102010112**	V	D	I	H	F	V	L	A	V	W	V	-909.626
**'0201040102010101070109**	I	D	V	R	F	V	L	V	T	W	Q	-909.626
**'0101010402010104020109**	V	D	I	Q	F	V	L	L	A	W	Q	-909.622
**'0101030103030101060107**	V	D	M	R	I	L	L	V	S	W	M	-909.622
**'0201010105020301010106**	I	D	I	R	V	I	V	V	V	W	L	-909.622
**'0101010304010105010104**	V	D	I	K	M	V	L	M	V	W	I	-909.621
**'0101030103020101060111**	V	D	M	R	I	I	L	V	S	W	T	-909.621
**'0101010103020104010106**	V	D	I	R	I	I	L	L	V	W	L	-909.621
**'0101040104030101040111**	V	D	V	R	M	L	L	V	L	W	T	-909.62
**'0201030104030101010104**	I	D	M	R	M	L	L	V	V	W	I	-909.616
**'0101030105010104070102**	V	D	M	R	V	V	L	L	T	W	E	-909.609
**'0101040104020103010108**	V	D	V	R	M	I	L	I	V	W	N	-909.594
**'0101040105030104070101**	V	D	V	R	V	L	L	L	T	W	H	-909.575
**'0101040105010101050102**	V	D	V	R	V	V	L	V	M	W	E	-909.574
**'0101040103010104070110**	V	D	V	R	I	V	L	L	T	W	R	-909.574
**'0101020102010104010105**	V	D	L	R	F	V	L	L	V	W	K	-909.573
**'0101010202020201070101**	V	D	I	H	F	I	A	V	T	W	H	-909.573
**'0101040305010101050105**	V	D	V	K	V	V	L	V	M	W	K	-909.571
**'0201010303030101010110**	I	D	I	K	I	L	L	V	V	W	R	-909.569
**'0201040102010101040108**	I	D	V	R	F	V	L	V	L	W	N	-909.567
**'0101010103010103050103**	V	D	I	R	I	V	L	I	M	W	F	-909.566
**'0201040102010102040101**	I	D	V	R	F	V	L	A	L	W	H	-909.564
**'0101010304010103060104**	V	D	I	K	M	V	L	I	S	W	I	-909.56
**'0101030103010101030106**	V	D	M	R	I	V	L	V	I	W	L	-909.56
**'0101020104010103010110**	V	D	L	R	M	V	L	I	V	W	R	-909.559
**'0301010205020101040101**	L	D	I	H	V	I	L	V	L	W	H	-909.559
**'0101010105020401010108**	V	D	I	R	V	I	W	V	V	W	N	-909.558
**'0101040102020102020101**	V	D	V	R	F	I	L	A	A	W	H	-909.558
**'0101010304040101070101**	V	D	I	K	M	M	L	V	T	W	H	-909.556
**'0101010201010104010104**	V	D	I	H	L	V	L	L	V	W	I	-909.551
**'0101010203020104070101**	V	D	I	H	I	I	L	L	T	W	H	-909.546
**'0101010203020101050101**	V	D	I	H	I	I	L	V	M	W	H	-909.541
**'0101010102010105040107**	V	D	I	R	F	V	L	M	L	W	M	-909.538
**'0101010105020101010104**	V	D	I	R	V	I	L	V	V	W	I	-909.536

'0101010305010103030103	V	D	I	K	V	V	L	I	I	W	F	-909.534
'0101040105010104050101	V	D	V	R	V	V	L	L	M	W	H	-909.53
'0101030102010105010104	V	D	M	R	F	V	L	M	V	W	I	-909.53
'0101010302010101040102	V	D	I	K	F	V	L	V	L	W	E	-909.528
'0101010104040201040106	V	D	I	R	M	M	A	V	L	W	L	-909.527
'0101040103030401010108	V	D	V	R	I	L	W	V	V	W	N	-909.525
'0101040103010201030105	V	D	V	R	I	V	A	V	I	W	K	-909.521
'0101030203010101030104	V	D	M	H	I	V	L	V	I	W	I	-909.521
'0101010105040201040101	V	D	I	R	V	M	A	V	L	W	H	-909.515
'0101010405010101040110	V	D	I	Q	V	V	L	V	L	W	R	-909.513
'0101010102010403010110	V	D	I	R	F	V	W	I	V	W	R	-909.512
'0101010402010104010110	V	D	I	Q	F	V	L	L	V	W	R	-909.512
'0101030405010101070110	V	D	M	Q	V	V	L	V	T	W	R	-909.504
'0101010302010104040104	V	D	I	K	F	V	L	L	L	W	I	-909.503
'0101010305030104010104	V	D	I	K	V	L	L	L	V	W	I	-909.5
'0301010305020101010106	L	D	I	K	V	I	L	V	V	W	L	-909.499
'0101010302030401010104	V	D	I	K	F	L	W	V	V	W	I	-909.499
'0201010302010101040105	I	D	I	K	F	V	L	V	L	W	K	-909.499
'0101010103020102050101	V	D	I	R	I	I	L	A	M	W	H	-909.499
'0101010203030104010108	V	D	I	H	I	L	L	L	V	W	N	-909.499
'0101010105010201030110	V	D	I	R	V	V	A	V	I	W	R	-909.496
'0201010105010201020104	I	D	I	R	V	V	A	V	A	W	I	-909.489
'0101010103040101070106	V	D	I	R	I	M	L	V	T	W	L	-909.489
'0201040105030301010101	I	D	V	R	V	L	V	V	V	W	H	-909.486
'0101040101030101040105	V	D	V	R	L	L	L	V	L	W	K	-909.486
'0101020103030101040110	V	D	L	R	I	L	L	V	L	W	R	-909.485
'0201010105010101070104	I	D	I	R	V	V	L	V	T	W	I	-909.485
'0101020105010101070110	V	D	L	R	V	V	L	V	T	W	R	-909.484
'0301030103010101040110	L	D	M	R	I	V	L	V	L	W	R	-909.481
'0101010402030101070109	V	D	I	Q	F	L	L	V	T	W	Q	-909.476
'0101010104020103040108	V	D	I	R	M	I	L	I	L	W	N	-909.475
'0201010102030103020101	I	D	I	R	F	L	L	I	A	W	H	-909.472
'0301010103020101050101	L	D	I	R	I	I	L	V	M	W	H	-909.471
'0101030201030201070101	V	D	M	H	L	L	A	V	T	W	H	-909.468
'0101010302010103040110	V	D	I	K	F	V	L	I	L	W	R	-909.466
'0101010102030401070104	V	D	I	R	F	L	W	V	T	W	I	-909.464
'0101030401030101040105	V	D	M	Q	L	L	L	V	L	W	K	-909.462

'0101010202010104030104	V	D	I	H	F	V	L	L	I	W	I	-909.46
'0101010304010104010111	V	D	I	K	M	V	L	L	V	W	T	-909.458
'0101040402020101010105	V	D	V	Q	F	I	L	V	V	W	K	-909.458
'0101010102030301060111	V	D	I	R	F	L	V	V	S	W	T	-909.452
'0101040302010104010101	V	D	V	K	F	V	L	L	V	W	H	-909.451
'0201010102030101020112	I	D	I	R	F	L	L	V	A	W	V	-909.445
'0101010403010101040101	V	D	I	Q	I	V	L	V	L	W	H	-909.445
'0301010102010102040104	L	D	I	R	F	V	L	A	L	W	I	-909.445
'0101020102010203010106	V	D	L	R	F	V	A	I	V	W	L	-909.444
'0101030105010104030108	V	D	M	R	V	V	L	L	I	W	N	-909.441
'0101040204010103010110	V	D	V	H	M	V	L	I	V	W	R	-909.441
'0101010205010105010112	V	D	I	H	V	V	L	M	V	W	V	-909.441
'0101010102010403020105	V	D	I	R	F	V	W	I	A	W	K	-909.441
'0101030104020101030105	V	D	M	R	M	I	L	V	I	W	K	-909.44
'0101040102010105070110	V	D	V	R	F	V	L	M	T	W	R	-909.439
'0101040103010404010104	V	D	V	R	I	V	W	L	V	W	I	-909.438
'0201010102010103070105	I	D	I	R	F	V	L	I	T	W	K	-909.43
'0101010103020103020105	V	D	I	R	I	I	L	I	A	W	K	-909.428
'0201030103040101010104	I	D	M	R	I	M	L	V	V	W	I	-909.427
'0101030403010101040112	V	D	M	Q	I	V	L	V	L	W	V	-909.423
'0101030103010104020101	V	D	M	R	I	V	L	L	A	W	H	-909.422
'0101010102020301070111	V	D	I	R	F	I	V	V	T	W	T	-909.421
'0101020105020101020112	V	D	L	R	V	I	L	V	A	W	V	-909.421
'0101040201010104040110	V	D	V	H	L	V	L	L	L	W	R	-909.421
'0101010303030101040107	V	D	I	K	I	L	L	V	L	W	M	-909.419
'0101010402010403010106	V	D	I	Q	F	V	W	I	V	W	L	-909.417
'0101010202010203050101	V	D	I	H	F	V	A	I	M	W	H	-909.412
'0101010105030104060104	V	D	I	R	V	L	L	L	S	W	I	-909.411
'0101010302030101040108	V	D	I	K	F	L	L	V	L	W	N	-909.41
'0201010102010105040110	I	D	I	R	F	V	L	M	L	W	R	-909.41
'0101020104010103040104	V	D	L	R	M	V	L	I	L	W	I	-909.407
'0101020203030101010101	V	D	L	H	I	L	L	V	V	W	H	-909.4
'0101010105010103010110	V	D	I	R	V	V	L	I	V	W	R	-909.399
'0101020103020201010105	V	D	L	R	I	I	A	V	V	W	K	-909.398
'0101010402010103020105	V	D	I	Q	F	V	L	I	A	W	K	-909.395
'0201030302010103010101	I	D	M	K	F	V	L	I	V	W	H	-909.395
'0101010105020301040106	V	D	I	R	V	I	V	V	L	W	L	-909.395

'0101040303020101010102	V	D	V	K	I	I	L	V	V	W	E	-909.395
'0301040105010104010104	L	D	V	R	V	V	L	L	V	W	I	-909.392
'0301010102010104020106	L	D	I	R	F	V	L	L	A	W	L	-909.388
'0101040402010103010109	V	D	V	Q	F	V	L	I	V	W	Q	-909.388
'0101030101020101030112	V	D	M	R	L	I	L	V	I	W	V	-909.387
'0101020205010101070112	V	D	L	H	V	V	L	V	T	W	V	-909.387
'0301010102030102010106	L	D	I	R	F	L	L	A	V	W	L	-909.385
'0101030302010104010106	V	D	M	K	F	V	L	L	V	W	L	-909.381
'0101010301020101070111	V	D	I	K	L	I	L	V	T	W	T	-909.379
'0301040102030102010101	L	D	V	R	F	L	L	A	V	W	H	-909.372
'0101040103010105070102	V	D	V	R	I	V	L	M	T	W	E	-909.371
'0101040105040101070106	V	D	V	R	V	M	L	V	T	W	L	-909.366
'0101030201020201040101	V	D	M	H	L	I	A	V	L	W	H	-909.365
'0201010102010103050110	I	D	I	R	F	V	L	I	M	W	R	-909.362
'0301020105020101010110	L	D	L	R	V	I	L	V	V	W	R	-909.359
'0201010302020101040101	I	D	I	K	F	I	L	V	L	W	H	-909.354
'0101030105020104010110	V	D	M	R	V	I	L	L	V	W	R	-909.353
'0101010103020401020108	V	D	I	R	I	I	W	V	A	W	N	-909.352
'0101010203010104070108	V	D	I	H	I	V	L	L	T	W	N	-909.349
'0101020302010101070106	V	D	L	K	F	V	L	V	T	W	L	-909.348
'0101010105010403010106	V	D	I	R	V	V	W	I	V	W	L	-909.348
'0101040204020101040101	V	D	V	H	M	I	L	V	L	W	H	-909.344
'0201010103010103070101	I	D	I	R	I	V	L	I	T	W	H	-909.343
'0101040104020101030102	V	D	V	R	M	I	L	V	I	W	E	-909.342
'0201010103030103010101	I	D	I	R	I	L	L	I	V	W	H	-909.341
'0101010102020102010102	V	D	I	R	F	I	L	A	V	W	E	-909.34
'0101010402020301010101	V	D	I	Q	F	I	V	V	V	W	H	-909.337
'0101010102020301040104	V	D	I	R	F	I	V	V	L	W	I	-909.336
'0101020204030101010106	V	D	L	H	M	L	L	V	V	W	L	-909.335
'0101010104010203040104	V	D	I	R	M	V	A	I	L	W	I	-909.332
'0301030105020101010109	L	D	M	R	V	I	L	V	V	W	Q	-909.331
'0101010103010403010104	V	D	I	R	I	V	W	I	V	W	I	-909.327
'0101010202030101060109	V	D	I	H	F	L	L	V	S	W	Q	-909.327
'0101030203020102010101	V	D	M	H	I	I	L	A	V	W	H	-909.323
'0301010205010104010108	L	D	I	H	V	V	L	L	V	W	N	-909.318
'0101020205020101050101	V	D	L	H	V	I	L	V	M	W	H	-909.314
'0201010305020101010112	I	D	I	K	V	I	L	V	V	W	V	-909.312

'0101040102030102010107	V	D	V	R	F	L	L	A	V	W	M	-909.308
'0101040202010103020101	V	D	V	H	F	V	L	I	A	W	H	-909.308
'0101010302010103050106	V	D	I	K	F	V	L	I	M	W	L	-909.307
'0101010104010104010106	V	D	I	R	M	V	L	L	V	W	L	-909.303
'0201040102010102010102	I	D	V	R	F	V	L	A	V	W	E	-909.3
'0101010104010103070101	V	D	I	R	M	V	L	I	T	W	H	-909.299
'0101040201030101030104	V	D	V	H	L	L	L	V	I	W	I	-909.298
'0101010303040101050110	V	D	I	K	I	M	L	V	M	W	R	-909.294
'0101010103030401040106	V	D	I	R	I	L	W	V	L	W	L	-909.294
'0201010103030102050101	I	D	I	R	I	L	L	A	M	W	H	-909.289
'0101030205010101020106	V	D	M	H	V	V	L	V	A	W	L	-909.289
'0101010304010103010106	V	D	I	K	M	V	L	I	V	W	L	-909.287
'0301030102010104010112	L	D	M	R	F	V	L	L	V	W	V	-909.286
'0201010103010101040110	I	D	I	R	I	V	L	V	L	W	R	-909.285
'0101010403040101050106	V	D	I	Q	I	M	L	V	M	W	L	-909.282
'0201040103010101010106	I	D	V	R	I	V	L	V	V	W	L	-909.278
'0101030401020103010105	V	D	M	Q	L	I	L	I	V	W	K	-909.277
'0201010105010103010108	I	D	I	R	V	V	L	I	V	W	N	-909.277
'0101010304010104040105	V	D	I	K	M	V	L	L	L	W	K	-909.271
'0101030102030104010104	V	D	M	R	F	L	L	L	V	W	I	-909.262
'0101010102020104030112	V	D	I	R	F	I	L	L	I	W	V	-909.261
'0101020302030101010102	V	D	L	K	F	L	L	V	V	W	E	-909.261
'0301010105020101030106	L	D	I	R	V	I	L	V	I	W	L	-909.26
'0201010102040201010106	I	D	I	R	F	M	A	V	V	W	L	-909.258
'0101020102030101060104	V	D	L	R	F	L	L	V	S	W	I	-909.254
'0101020201030103010106	V	D	L	H	L	L	L	I	V	W	L	-909.254
'0101010102030201050104	V	D	I	R	F	L	A	V	M	W	I	-909.253
'0101030103020401070101	V	D	M	R	I	I	W	V	T	W	H	-909.252
'0101010103010303010101	V	D	I	R	I	V	V	I	V	W	H	-909.243
'0101030302010101010102	V	D	M	K	F	V	L	V	V	W	E	-909.24
'0101010402030201040101	V	D	I	Q	F	L	A	V	L	W	H	-909.235
'0101040202010101060101	V	D	V	H	F	V	L	V	S	W	H	-909.234
'0101030403010103010104	V	D	M	Q	I	V	L	I	V	W	I	-909.232
'0201040102010103050101	I	D	V	R	F	V	L	I	M	W	H	-909.232
'0201010105030201010112	I	D	I	R	V	L	A	V	V	W	V	-909.232
'0101040201020101030112	V	D	V	H	L	I	L	V	I	W	V	-909.227
'0101030104040101010108	V	D	M	R	M	M	L	V	V	W	N	-909.221

'0301010103030102010104	L	D	I	R	I	L	L	A	V	W	I	-909.218
'0101040201020103010106	V	D	V	H	L	I	L	I	V	W	L	-909.218
'0101010204010105040104	V	D	I	H	M	V	L	M	L	W	I	-909.217
'0101010202040102010104	V	D	I	H	F	M	L	A	V	W	I	-909.212
'0101040303010101030110	V	D	V	K	I	V	L	V	I	W	R	-909.212
'0201010202020101010103	I	D	I	H	F	I	L	V	V	W	F	-909.209
'0101010104030104040104	V	D	I	R	M	L	L	L	L	W	I	-909.208
'0201010103020101030103	I	D	I	R	I	I	L	V	I	W	F	-909.206
'0101010102020401070102	V	D	I	R	F	I	W	V	T	W	E	-909.204
'0101030202010101060107	V	D	M	H	F	V	L	V	S	W	M	-909.197
'0101020402010101010104	V	D	L	Q	F	V	L	V	V	W	I	-909.195
'0101030101020103030108	V	D	M	R	L	I	L	I	I	W	N	-909.19
'0101010202040201040101	V	D	I	H	F	M	A	V	L	W	H	-909.189
'0101010105030103010105	V	D	I	R	V	L	L	I	V	W	K	-909.188
'0301010103010102040106	L	D	I	R	I	V	L	A	L	W	L	-909.187
'0101010302020201020101	V	D	I	K	F	I	A	V	A	W	H	-909.182
'0101030201020101050109	V	D	M	H	L	I	L	V	M	W	Q	-909.178
'0201010105010105070110	I	D	I	R	V	V	L	M	T	W	R	-909.171
'0101030304010101040102	V	D	M	K	M	V	L	V	L	W	E	-909.169
'0101010105030104040105	V	D	I	R	V	L	L	L	L	W	K	-909.166
'0101020105030101040109	V	D	L	R	V	L	L	V	L	W	Q	-909.165
'0201040105010101030112	I	D	V	R	V	V	L	V	I	W	V	-909.164
'0101010202040101030111	V	D	I	H	F	M	L	V	I	W	T	-909.163
'0101030103010201050110	V	D	M	R	I	V	A	V	M	W	R	-909.161
'0101030105010103010107	V	D	M	R	V	V	L	I	V	W	M	-909.16
'0201010405010103070101	I	D	I	Q	V	V	L	I	T	W	H	-909.158
'0101010105010104050110	V	D	I	R	V	V	L	L	M	W	R	-909.158
'0301010103010101070105	L	D	I	R	I	V	L	V	T	W	K	-909.155
'0101020101020101010110	V	D	L	R	L	I	L	V	V	W	R	-909.149
'0101040105010303010108	V	D	V	R	V	V	V	I	V	W	N	-909.149
'0301010203010101010104	L	D	I	H	I	V	L	V	V	W	I	-909.145
'0201010102010105070106	I	D	I	R	F	V	L	M	T	W	L	-909.14
'0101010102010102020101	V	D	I	R	F	V	L	A	A	W	H	-909.139
'0201010105030102040101	I	D	I	R	V	L	L	A	L	W	H	-909.135
'0201010102010303010106	I	D	I	R	F	V	V	I	V	W	L	-909.135
'0101030102030401010105	V	D	M	R	F	L	W	V	V	W	K	-909.134
'0201010205020101070101	I	D	I	H	V	I	L	V	T	W	H	-909.134

**'010102010201010307010 7**	V	D	L	R	F	V	L	I	T	W	M	-909.134
**'0101020102010103050102**	V	D	L	R	F	V	L	I	M	W	E	-909.134
**'0101010205010101030109**	V	D	I	H	V	V	L	V	I	W	Q	-909.132
**'0101010201030101040108**	V	D	I	H	L	L	L	V	L	W	N	-909.13
**'0101020203010101010110**	V	D	L	H	I	V	L	V	V	W	R	-909.129
**'0101010105020301030105**	V	D	I	R	V	I	V	V	I	W	K	-909.128
**'0101010103010104010105**	V	D	I	R	I	V	L	L	V	W	K	-909.126
**'0101040103030101010105**	V	D	V	R	I	L	L	V	V	W	K	-909.125
**'0101040101020103040102**	V	D	V	R	L	I	L	I	L	W	E	-909.123
**'0101020102010104070112**	V	D	L	R	F	V	L	L	T	W	V	-909.122
**'0201010203020201010101**	I	D	I	H	I	I	A	V	V	W	H	-909.12
**'0101040202010401010105**	V	D	V	H	F	V	W	V	V	W	K	-909.114
**'0101010102010303040110**	V	D	I	R	F	V	V	I	L	W	R	-909.111
**'0101020205020101010107**	V	D	L	H	V	I	L	V	V	W	M	-909.11
**'0101010402010103040111**	V	D	I	Q	F	V	L	I	L	W	T	-909.109
**'0101010402040101050110**	V	D	I	Q	F	M	L	V	M	W	R	-909.107
**'0201040102030101050101**	I	D	V	R	F	L	L	V	M	W	H	-909.103
**'0101020202020101030101**	V	D	L	H	F	I	L	V	I	W	H	-909.103
**'0301010102010101040107**	L	D	I	R	F	V	L	V	L	W	M	-909.102
**'0101010305010101040101**	V	D	I	K	V	V	L	V	L	W	H	-909.098
**'0101040404020101010101**	V	D	V	Q	M	I	L	V	V	W	H	-909.097
**'0201040402010103010101**	I	D	V	Q	F	V	L	I	V	W	H	-909.096
**'0101010204030101070101**	V	D	I	H	M	L	L	V	T	W	H	-909.094
**'0301010105010104020112**	L	D	I	R	V	V	L	L	A	W	V	-909.094
**'0301010405010104010104**	L	D	I	Q	V	V	L	L	V	W	I	-909.093
**'0101010105020301060108**	V	D	I	R	V	I	V	V	S	W	N	-909.091
**'0301040105040101010104**	L	D	V	R	V	M	L	V	V	W	I	-909.089
**'0101010102030301030112**	V	D	I	R	F	L	V	V	I	W	V	-909.087
**'0101010205030201010109**	V	D	I	H	V	L	A	V	V	W	Q	-909.086
**'0101030102040101020109**	V	D	M	R	F	M	L	V	A	W	Q	-909.085
**'0201030103010103010105**	I	D	M	R	I	V	L	I	V	W	K	-909.084
**'0101010103020403010102**	V	D	I	R	I	I	W	I	V	W	E	-909.083
**'0101010403010103040110**	V	D	I	Q	I	V	L	I	L	W	R	-909.082
**'0101040202010301010106**	V	D	V	H	F	V	V	V	V	W	L	-909.082
**'0101040402010101070110**	V	D	V	Q	F	V	L	V	T	W	R	-909.08
**'0301010103020104010104**	L	D	I	R	I	I	L	L	V	W	I	-909.072
**'0101010103020103070110**	V	D	I	R	I	I	L	I	T	W	R	-909.072

**'0101010105040301070101**	V	D	I	R	V	M	V	V	T	W	H	-909.069
**'0101030202010101030112**	V	D	M	H	F	V	L	V	I	W	V	-909.068
**'0101010403030103010106**	V	D	I	Q	I	L	L	I	V	W	L	-909.066
**'0101040104030101030105**	V	D	V	R	M	L	L	V	I	W	K	-909.064
**'0101010102040102040104**	V	D	I	R	F	M	L	A	L	W	I	-909.062
**'0101010202010303010110**	V	D	I	H	F	V	V	I	V	W	R	-909.06
**'0201040102010101020111**	I	D	V	R	F	V	L	V	A	W	T	-909.057
**'0101010102020303010107**	V	D	I	R	F	I	V	I	V	W	M	-909.057
**'0201010403030101010106**	I	D	I	Q	I	L	L	V	V	W	L	-909.052
**'0101020104030103010104**	V	D	L	R	M	L	L	I	V	W	I	-909.051
**'0101040401020103010108**	V	D	V	Q	L	I	L	I	V	W	N	-909.05
**'0101010105020201050104**	V	D	I	R	V	I	A	V	M	W	I	-909.046
**'0101030403030101060101**	V	D	M	Q	I	L	L	V	S	W	H	-909.046
**'0101030203030101040101**	V	D	M	H	I	L	L	V	L	W	H	-909.043
**'0101010205010201040111**	V	D	I	H	V	V	A	V	L	W	T	-909.042
**'0201010204030101010104**	I	D	I	H	M	L	L	V	V	W	I	-909.041
**'0101020103020101060112**	V	D	L	R	I	I	L	V	S	W	V	-909.041
**'0301040102010102010106**	L	D	V	R	F	V	L	A	V	W	L	-909.039
**'0101020103020103070101**	V	D	L	R	I	I	L	I	T	W	H	-909.038
**'0201010102010102020104**	I	D	I	R	F	V	L	A	A	W	I	-909.036
**'0301010205020104010101**	L	D	I	H	V	I	L	L	V	W	H	-909.036
**'0101010305020104020101**	V	D	I	K	V	I	L	L	A	W	H	-909.031
**'0101040302010101030109**	V	D	V	K	F	V	L	V	I	W	Q	-909.026
**'0201010402020101010109**	I	D	I	Q	F	I	L	V	V	W	Q	-909.023
**'0101010102030102050104**	V	D	I	R	F	L	L	A	M	W	I	-909.023
**'0301010102020101070104**	L	D	I	R	F	I	L	V	T	W	I	-909.018
**'0101040403030103010101**	V	D	V	Q	I	L	L	I	V	W	H	-909.017
**'0201040105010104010105**	I	D	V	R	V	V	L	L	V	W	K	-909.017
**'0101020202010104010104**	V	D	L	H	F	V	L	L	V	W	I	-909.014
**'0101010404010103030104**	V	D	I	Q	M	V	L	I	I	W	I	-909.014
**'0201010105010104020106**	I	D	I	R	V	V	L	L	A	W	L	-909.014
**'0101010203020301010109**	V	D	I	H	I	I	V	V	V	W	Q	-909.011
**'0101040404010103050101**	V	D	V	Q	M	V	L	I	M	W	H	-909.008
**'0101030105020101060112**	V	D	M	R	V	I	L	V	S	W	V	-909.007
**'0101010402030101020104**	V	D	I	Q	F	L	L	V	A	W	I	-909.007
**'0201010402020101070101**	I	D	I	Q	F	I	L	V	T	W	H	-909.006
**'0101040103010301040106**	V	D	V	R	I	V	V	V	L	W	L	-909.005

'0101040102040101020106	V	D	V	R	F	M	L	V	A	W	L	-909.004
'0101030403030101010111	V	D	M	Q	I	L	L	V	V	W	T	-909.003
'0101010202020102020101	V	D	I	H	F	I	L	A	A	W	H	-909.0
'0101030105010304010106	V	D	M	R	V	V	V	L	V	W	L	-908.996
'0101040405010104010111	V	D	V	Q	V	V	L	L	V	W	T	-908.994
'0101010103020101060106	V	D	I	R	I	I	L	V	S	W	L	-908.991
'0201010102030101060106	I	D	I	R	F	L	L	V	S	W	L	-908.987
'0101030105030104010112	V	D	M	R	V	L	L	L	V	W	V	-908.986
'0101010202010103070105	V	D	I	H	F	V	L	I	T	W	K	-908.985
'0201010103020101020109	I	D	I	R	I	I	L	V	A	W	Q	-908.984
'0101030104040101070106	V	D	M	R	M	M	L	V	T	W	L	-908.984
'0101030101030101030105	V	D	M	R	L	L	L	V	I	W	K	-908.983
'0101040101020103050104	V	D	V	R	L	I	L	I	M	W	I	-908.981
'0101010102010403040108	V	D	I	R	F	V	W	I	L	W	N	-908.98
'0101040103040101010110	V	D	V	R	I	M	L	V	V	W	R	-908.98
'0101030203040101060101	V	D	M	H	I	M	L	V	S	W	H	-908.979
'0201010102040101060101	I	D	I	R	F	M	L	V	S	W	H	-908.976
'0101010205030104010112	V	D	I	H	V	L	L	L	V	W	V	-908.976
'0101010103010201040109	V	D	I	R	I	V	A	V	L	W	Q	-908.968
'0101030101030101020112	V	D	M	R	L	L	L	V	A	W	V	-908.968
'0201010205040101020101	I	D	I	H	V	M	L	V	A	W	H	-908.967
'0301030403020101010101	L	D	M	Q	I	I	L	V	V	W	H	-908.965
'0101040103010105020105	V	D	V	R	I	V	L	M	A	W	K	-908.963
'0101040105010101030105	V	D	V	R	V	V	L	V	I	W	K	-908.959
'0101020104020101040106	V	D	L	R	M	I	L	V	L	W	L	-908.958
'0101010205030101030103	V	D	I	H	V	L	L	V	I	W	F	-908.958
'0101010403020101010109	V	D	I	Q	I	I	L	V	V	W	Q	-908.956
'0301010105010104010101	L	D	I	R	V	V	L	L	V	W	H	-908.953
'0101010102020403010111	V	D	I	R	F	I	W	I	V	W	T	-908.953
'0101010102020304010110	V	D	I	R	F	I	V	L	V	W	R	-908.95
'0201020102020101020101	I	D	L	R	F	I	L	V	A	W	H	-908.946
'0101010204010103040112	V	D	I	H	M	V	L	I	L	W	V	-908.945
'0101040104010103010103	V	D	V	R	M	V	L	I	V	W	F	-908.944
'0101040402010103070101	V	D	V	Q	F	V	L	I	T	W	H	-908.942
'0301010104020101020104	L	D	I	R	M	I	L	V	A	W	I	-908.94
'0101010305020101050107	V	D	I	K	V	I	L	V	M	W	M	-908.939
'0101010305030101030109	V	D	I	K	V	L	L	V	I	W	Q	-908.937

**'01010201050101010401 05**	V	D	L	R	V	V	L	V	L	W	K	-908.935
**'010104010502040101010 4**	V	D	V	R	V	I	W	V	V	W	I	-908.933
**'010103010402010106010 8**	V	D	M	R	M	I	L	V	S	W	N	-908.933
**'010104010302010502010 1**	V	D	V	R	I	I	L	M	A	W	H	-908.932
**'030104030501010101010 6**	L	D	V	K	V	V	L	V	V	W	L	-908.931
**'020101010303030107010 1**	I	D	I	R	I	L	V	V	T	W	H	-908.93
**'010104010201010204010 9**	V	D	V	R	F	V	L	A	L	W	Q	-908.928
**'010104040501010103010 4**	V	D	V	Q	V	V	L	V	I	W	I	-908.923
**'020101020204010104010 1**	I	D	I	H	F	M	L	V	L	W	H	-908.922
**'010101030502040101010 5**	V	D	I	K	V	I	W	V	V	W	K	-908.92
**'020102010302010101011 2**	I	D	L	R	I	I	L	V	V	W	V	-908.919
**'010101020302010104010 7**	V	D	I	H	I	I	L	V	L	W	M	-908.914
**'010101030202010105011 0**	V	D	I	K	F	I	L	V	M	W	R	-908.913
**'010104010302020101010 7**	V	D	V	R	I	I	A	V	V	W	M	-908.904
**'010101010302030106010 2**	V	D	I	R	I	I	V	V	S	W	E	-908.904
**'010102020401010301010 2**	V	D	L	H	M	V	L	I	V	W	E	-908.904
**'010101030201040301010 4**	V	D	I	K	F	V	W	I	V	W	I	-908.897
**'020101030201010307010 1**	I	D	I	K	F	V	L	I	T	W	H	-908.897
**'010101030201010504010 6**	V	D	I	K	F	V	L	M	L	W	L	-908.896
**'020104010303020101010 1**	I	D	V	R	I	L	A	V	V	W	H	-908.894
**'010101010503010107010 2**	V	D	I	R	V	L	L	V	T	W	E	-908.893
**'010102020501010102010 9**	V	D	L	H	V	V	L	V	A	W	Q	-908.891
**'010103030501010107010 8**	V	D	M	K	V	V	L	V	T	W	N	-908.889
**'020101020501010501010 8**	I	D	I	H	V	V	L	M	V	W	N	-908.888
**'010104040201010105010 9**	V	D	V	Q	F	V	L	V	M	W	Q	-908.887
**'020101010203020101010 1**	I	D	I	R	F	L	A	V	V	W	H	-908.886
**'010101020204010107010 2**	V	D	I	H	F	M	L	V	T	W	E	-908.885
**'010101010203030304010 1**	V	D	I	R	F	L	V	I	L	W	H	-908.884
**'020101020301010201010 3**	I	D	I	H	I	V	L	A	V	W	F	-908.884
**'010103040304010101010 6**	V	D	M	Q	I	M	L	V	V	W	L	-908.884
**'020101010201010107010 1**	I	D	I	R	F	V	L	V	T	W	H	-908.883
**'010101010304010101010 3**	V	D	I	R	I	M	L	V	V	W	F	-908.883
**'010101020101020304010 4**	V	D	I	H	L	V	A	I	L	W	I	-908.881
**'010102010303010301011 2**	V	D	L	R	I	L	L	I	V	W	V	-908.881
**'010101020301010402010 4**	V	D	I	H	I	V	L	L	A	W	I	-908.878
**'010103010402010102011 0**	V	D	M	R	M	I	L	V	A	W	R	-908.871
**'010101010204020102011 0**	V	D	I	R	F	M	A	V	A	W	R	-908.871

**'0201010103010104010104**	I	D	I	R	I	V	L	L	V	W	I	-908.87
**'0201010102010403010105**	I	D	I	R	F	V	W	I	V	W	K	-908.87
**'0101040203020201010101**	V	D	V	H	I	I	A	V	V	W	H	-908.867
**'0301040103010102010104**	L	D	V	R	I	V	L	A	V	W	I	-908.867
**'0101010402020101010108**	V	D	I	Q	F	I	L	V	V	W	N	-908.865
**'0101040205040101010109**	V	D	V	H	V	M	L	V	V	W	Q	-908.864
**'0301010102020104010108**	L	D	I	R	F	I	L	L	V	W	N	-908.86
**'0101010204010104040106**	V	D	I	H	M	V	L	L	L	W	L	-908.859
**'0101010102010101060108**	V	D	I	R	F	V	L	V	S	W	N	-908.859
**'0101040102040103010106**	V	D	V	R	F	M	L	I	V	W	L	-908.857
**'0101040103030301010107**	V	D	V	R	I	L	V	V	V	W	M	-908.857
**'0101010102010301040108**	V	D	I	R	F	V	V	V	L	W	N	-908.855
**'0101010204040101010103**	V	D	I	H	M	M	L	V	V	W	F	-908.852
**'0201030102040101010106**	I	D	M	R	F	M	L	V	V	W	L	-908.841
**'0101030203010102040101**	V	D	M	H	I	V	L	A	L	W	H	-908.84
**'0101010403020401010104**	V	D	I	Q	I	I	W	V	V	W	I	-908.84
**'0101010204010103050104**	V	D	I	H	M	V	L	I	M	W	I	-908.835
**'0101040105020105050101**	V	D	V	R	V	I	L	M	M	W	H	-908.834
**'0101040102040101010111**	V	D	V	R	F	M	L	V	V	W	T	-908.833
**'0101040102010303010101**	V	D	V	R	F	V	V	I	V	W	H	-908.832
**'0201010104030101010101**	I	D	I	R	M	L	L	V	V	W	H	-908.831
**'0201010304040101010101**	I	D	I	K	M	M	L	V	V	W	H	-908.829
**'0101040105020201010108**	V	D	V	R	V	I	A	V	V	W	N	-908.823
**'0201010402030101040101**	I	D	I	Q	F	L	L	V	L	W	H	-908.82
**'0101040103020102010105**	V	D	V	R	I	I	L	A	V	W	K	-908.819
**'0101030303010101050105**	V	D	M	K	I	V	L	V	M	W	K	-908.817
**'0201040305010103010101**	I	D	V	K	V	V	L	I	V	W	H	-908.817
**'0101010103010103030104**	V	D	I	R	I	V	L	I	I	W	I	-908.817
**'0101010203030103070101**	V	D	I	H	I	L	L	I	T	W	H	-908.817
**'0201040102010101050105**	I	D	V	R	F	V	L	V	M	W	K	-908.816
**'0101010203040101020101**	V	D	I	H	I	M	L	V	A	W	H	-908.813
**'0101030103040101070109**	V	D	M	R	I	M	L	V	T	W	Q	-908.812
**'0101010204040101050101**	V	D	I	H	M	M	L	V	M	W	H	-908.812
**'0101010102020401040112**	V	D	I	R	F	I	W	V	L	W	V	-908.81
**'0101010103020301050112**	V	D	I	R	I	I	V	V	M	W	V	-908.809
**'0101030305010101050110**	V	D	M	K	V	V	L	V	M	W	R	-908.807
**'0201030203010101010109**	I	D	M	H	I	V	L	V	V	W	Q	-908.807

'0101010102020103060109	V	D	I	R	F	I	L	I	S	W	Q	-908.807
'0101010203010103050111	V	D	I	H	I	V	L	I	M	W	T	-908.806
'0101030102040301070101	V	D	M	R	F	M	V	V	T	W	H	-908.806
'0101030105010201010110	V	D	M	R	V	V	A	V	V	W	R	-908.804
'0101040102020301010109	V	D	V	R	F	I	V	V	V	W	Q	-908.803
'0101010402030104010106	V	D	I	Q	F	L	L	L	V	W	L	-908.802
'0101040402030104010101	V	D	V	Q	F	L	L	L	V	W	H	-908.802
'0101010202010401010110	V	D	I	H	F	V	W	V	V	W	R	-908.801
'0201040205010101010109	I	D	V	H	V	V	L	V	V	W	Q	-908.8
'0101010105030303010101	V	D	I	R	V	L	V	I	V	W	H	-908.8
'0101010101020101040105	V	D	I	R	L	I	L	V	L	W	K	-908.799
'0101010205010104050104	V	D	I	H	V	V	L	L	M	W	I	-908.799
'0101040301020101010106	V	D	V	K	L	I	L	V	V	W	L	-908.798
'0301010402010101040106	L	D	I	Q	F	V	L	V	L	W	L	-908.794
'0101040102010301020101	V	D	V	R	F	V	V	V	A	W	H	-908.793
'0101030103030103020101	V	D	M	R	I	L	L	I	A	W	H	-908.792
'0201010102040101040106	I	D	I	R	F	M	L	V	L	W	L	-908.791
'0101040105010103010112	V	D	V	R	V	V	L	I	V	W	V	-908.79
'0101010302010203010106	V	D	I	K	F	V	A	I	V	W	L	-908.79
'0101030401020101030106	V	D	M	Q	L	I	L	V	I	W	L	-908.788
'0101020202010103030101	V	D	L	H	F	V	L	I	I	W	H	-908.783
'0101020101020101040104	V	D	L	R	L	I	L	V	L	W	I	-908.781
'0101010205040201010105	V	D	I	H	V	M	A	V	V	W	K	-908.779
'0201030202010101050101	I	D	M	H	F	V	L	V	M	W	H	-908.776
'0201020105020201010101	I	D	L	R	V	I	A	V	V	W	H	-908.774
'0101010303040101030112	V	D	I	K	I	M	L	V	I	W	V	-908.773
'0301010203010102010101	L	D	I	H	I	V	L	A	V	W	H	-908.77
'0101010201040201040106	V	D	I	H	L	M	A	V	L	W	L	-908.769
'0101040103010403010101	V	D	V	R	I	V	W	I	V	W	H	-908.764
'0101010103030101030106	V	D	I	R	I	L	L	V	I	W	L	-908.763
'0101040105030103010111	V	D	V	R	V	L	L	I	V	W	T	-908.757
'0101010105030301020109	V	D	I	R	V	L	V	V	A	W	Q	-908.756
'0101040103010103020106	V	D	V	R	I	V	L	I	A	W	L	-908.752
'0101010102010204010101	V	D	I	R	F	V	A	L	V	W	H	-908.751
'0301020102020101010104	L	D	L	R	F	I	L	V	V	W	I	-908.748
'0101040102040101050101	V	D	V	R	F	M	L	V	M	W	H	-908.747
'0101020102010103060109	V	D	L	R	F	V	L	I	S	W	Q	-908.743

**'0101010205030102040101**	V	D	I	H	V	L	L	A	L	W	H	-908.743
**'0101010105010102040112**	V	D	I	R	V	V	L	A	L	W	V	-908.743
**'0201010405010101040106**	I	D	I	Q	V	V	L	V	L	W	L	-908.74
**'0101030103010103020104**	V	D	M	R	I	V	L	I	A	W	I	-908.739
**'0201030205010101010104**	I	D	M	H	V	V	L	V	V	W	I	-908.736
**'0101010304040101040106**	V	D	I	K	M	M	L	V	L	W	L	-908.735
**'0301010103010102010105**	L	D	I	R	I	V	L	A	V	W	K	-908.729
**'0201020103020101070101**	I	D	L	R	I	I	L	V	T	W	H	-908.728
**'0101010104040101040105**	V	D	I	R	M	M	L	V	L	W	K	-908.725
**'0101020102020104010108**	V	D	L	R	F	I	L	L	V	W	N	-908.725
**'0101040201010104070106**	V	D	V	H	L	V	L	L	T	W	L	-908.722
**'0101040201020104010104**	V	D	V	H	L	I	L	L	V	W	I	-908.716
**'0101020201020101070101**	V	D	L	H	L	I	L	V	T	W	H	-908.714
**'0101010105010303010105**	V	D	I	R	V	V	V	I	V	W	K	-908.713
**'0301040105010102010101**	L	D	V	R	V	V	L	A	V	W	H	-908.71
**'0101040101020101040107**	V	D	V	R	L	I	L	V	L	W	M	-908.71
**'0101040305030101070101**	V	D	V	K	V	L	L	V	T	W	H	-908.708
**'0101010104010101040104**	V	D	I	R	M	V	L	V	L	W	I	-908.707
**'0101030205010105010106**	V	D	M	H	V	V	L	M	V	W	L	-908.704
**'0101010202010104050108**	V	D	I	H	F	V	L	L	M	W	N	-908.704
**'0101040103040101020111**	V	D	V	R	I	M	L	V	A	W	T	-908.702
**'0101030201030101050107**	V	D	M	H	L	L	L	V	M	W	M	-908.697
**'0101010202030104040101**	V	D	I	H	F	L	L	L	L	W	H	-908.695
**'0101030101030101010104**	V	D	M	R	L	L	L	V	V	W	I	-908.694
**'0101040105010301010112**	V	D	V	R	V	V	V	V	V	W	V	-908.691
**'0301010105010101070108**	L	D	I	R	V	V	L	V	T	W	N	-908.69
**'0101020104030101070106**	V	D	L	R	M	L	L	V	T	W	L	-908.688
**'0101040102010105050101**	V	D	V	R	F	V	L	M	M	W	H	-908.688
**'0101010402010105040104**	V	D	I	Q	F	V	L	M	L	W	I	-908.688
**'0101030201030101020110**	V	D	M	H	L	L	L	V	A	W	R	-908.685
**'0101030103010103030107**	V	D	M	R	I	V	L	I	I	W	M	-908.684
**'0201040104020101010109**	I	D	V	R	M	I	L	V	V	W	Q	-908.68
**'0301040105010101040108**	L	D	V	R	V	V	L	V	L	W	N	-908.678
**'0101030103030301050101**	V	D	M	R	I	L	V	V	M	W	H	-908.676
**'0201010104030101040105**	I	D	I	R	M	L	L	V	L	W	K	-908.675
**'0301010103010104010108**	L	D	I	R	I	V	L	L	V	W	N	-908.669
**'0301010205040101010105**	L	D	I	H	V	M	L	V	V	W	K	-908.666

'0101010202010304010102	V	D	I	H	F	V	V	L	V	W	E	-908.666
'0101030102010101060105	V	D	M	R	F	V	L	V	S	W	K	-908.666
'0101010202020103070101	V	D	I	H	F	I	L	I	T	W	H	-908.666
'0201010403030101060101	I	D	I	Q	I	L	L	V	S	W	H	-908.66
'0301010102010102070109	L	D	I	R	F	V	L	A	T	W	Q	-908.66
'0101040102020201010104	V	D	V	R	F	I	A	V	V	W	I	-908.659
'0101010105020401040109	V	D	I	R	V	I	W	V	L	W	Q	-908.659
'0101030105030101030109	V	D	M	R	V	L	L	V	I	W	Q	-908.657
'0101020402010101070101	V	D	L	Q	F	V	L	V	T	W	H	-908.654
'0101010105020401070107	V	D	I	R	V	I	W	V	T	W	M	-908.654
'0101010103020301020106	V	D	I	R	I	I	V	V	A	W	L	-908.653
'0101040302010105010106	V	D	V	K	F	V	L	M	V	W	L	-908.649
'0101030105010204010108	V	D	M	R	V	V	A	L	V	W	N	-908.648
'0101040202010104010110	V	D	V	H	F	V	L	L	V	W	R	-908.645
'0101040103010204010105	V	D	V	R	I	V	A	L	V	W	K	-908.643
'0201040104010103040101	I	D	V	R	M	V	L	I	L	W	H	-908.639
'0101030103020104010101	V	D	M	R	I	I	L	L	V	W	H	-908.639
'0101030201010103020105	V	D	M	H	L	V	L	I	A	W	K	-908.638
'0201010201030101020107	I	D	I	H	L	L	L	V	A	W	M	-908.635
'0101020105020101050103	V	D	L	R	V	I	L	V	M	W	F	-908.634
'0301040405010101010104	L	D	V	Q	V	V	L	V	V	W	I	-908.632
'0101040104030101010107	V	D	V	R	M	L	L	V	V	W	M	-908.63
'0101040105020301010101	V	D	V	R	V	I	V	V	V	W	H	-908.629
'0301010102040101010105	L	D	I	R	F	M	L	V	V	W	K	-908.623
'0101030102010105020101	V	D	M	R	F	V	L	M	A	W	H	-908.623
'0201010402010105010104	I	D	I	Q	F	V	L	M	V	W	I	-908.621
'0101040202010102040101	V	D	V	H	F	V	L	A	L	W	H	-908.621
'0101040403020105010101	V	D	V	Q	I	I	L	M	V	W	H	-908.619
'0101040302030101060101	V	D	V	K	F	L	L	V	S	W	H	-908.618
'0101030201020101060112	V	D	M	H	L	I	L	V	S	W	V	-908.618
'0101030105010104020103	V	D	M	R	V	V	L	L	A	W	F	-908.617
'0101040103010101070109	V	D	V	R	I	V	L	V	T	W	Q	-908.617
'0101030203040103010101	V	D	M	H	I	M	L	I	V	W	H	-908.616
'0301010402020101010105	L	D	I	Q	F	I	L	V	V	W	K	-908.615
'0101010102030401040105	V	D	I	R	F	L	W	V	L	W	K	-908.613
'0101010404010101040109	V	D	I	Q	M	V	L	V	L	W	Q	-908.608
'0101040105010105050104	V	D	V	R	V	V	L	M	M	W	I	-908.606

'0101030405030101020101	V	D	M	Q	V	L	L	V	A	W	H	-908.603
'0301010202010105010109	L	D	I	H	F	V	L	M	V	W	Q	-908.602
'0201030205040101010101	I	D	M	H	V	M	L	V	V	W	H	-908.6
'0101010103040101060101	V	D	I	R	I	M	L	V	S	W	H	-908.598
'0101010105020103070105	V	D	I	R	V	I	L	I	T	W	K	-908.597
'0101010102030301070106	V	D	I	R	F	L	V	V	T	W	L	-908.594
'0201010104010105010101	I	D	I	R	M	V	L	M	V	W	H	-908.592
'0101010301010103030106	V	D	I	K	L	V	L	I	I	W	L	-908.586
'0101010404030101010106	V	D	I	Q	M	L	L	V	V	W	L	-908.586
'0301040302010101010105	L	D	V	K	F	V	L	V	V	W	K	-908.584
'0101030105010201050101	V	D	M	R	V	V	A	V	M	W	H	-908.582
'0201030103010101020105	I	D	M	R	I	V	L	V	A	W	K	-908.581
'0201010102030301010102	I	D	I	R	F	L	V	V	V	W	E	-908.575
'0101010402040101010104	V	D	I	Q	F	M	L	V	V	W	I	-908.571
'0101010205030301010105	V	D	I	H	V	L	V	V	V	W	K	-908.571
'0101030102010104060108	V	D	M	R	F	V	L	L	S	W	N	-908.57
'0301010205010101010106	L	D	I	H	V	V	L	V	V	W	L	-908.569
'0101040102030304010101	V	D	V	R	F	L	V	L	V	W	H	-908.569
'0101030102040201010109	V	D	M	R	F	M	A	V	V	W	Q	-908.568
'0101010204020104010110	V	D	I	H	M	I	L	L	V	W	R	-908.567
'0101010204020103030101	V	D	I	H	M	I	L	I	I	W	H	-908.566
'0101010105030201020105	V	D	I	R	V	L	A	V	A	W	K	-908.563
'0201020105010101020106	I	D	L	R	V	V	L	V	A	W	L	-908.562
'0101040103010301070110	V	D	V	R	I	V	V	V	T	W	R	-908.562
'0101010205010104030112	V	D	I	H	V	V	L	L	I	W	V	-908.561
'0101010205010102040109	V	D	I	H	V	V	L	A	L	W	Q	-908.56
'0101030102030102020101	V	D	M	R	F	L	L	A	A	W	H	-908.56
'0101040405010101050108	V	D	V	Q	V	V	L	V	M	W	N	-908.558
'0101030104010101040107	V	D	M	R	M	V	L	V	L	W	M	-908.557
'0101010102040101060106	V	D	I	R	F	M	L	V	S	W	L	-908.556
'0101040404010104040101	V	D	V	Q	M	V	L	L	L	W	H	-908.556
'0201010102030402010101	I	D	I	R	F	L	W	A	V	W	H	-908.554
'0101030101010101040108	V	D	M	R	L	V	L	V	L	W	N	-908.553
'0101010105010301070106	V	D	I	R	V	V	V	V	T	W	L	-908.551
'0101010203020104010107	V	D	I	H	I	I	L	L	V	W	M	-908.551
'0201010103020401010105	I	D	I	R	I	I	W	V	V	W	K	-908.548
'0101020404010103010101	V	D	L	Q	M	V	L	I	V	W	H	-908.548

'0101040405010105010106	V	D	V	Q	V	V	L	M	V	W	L	-908.547
'0101030202010201010104	V	D	M	H	F	V	A	V	V	W	I	-908.546
'0101010205020401010110	V	D	I	H	V	I	W	V	V	W	R	-908.545
'0101010102020104020108	V	D	I	R	F	I	L	L	A	W	N	-908.544
'0101020205010101010103	V	D	L	H	V	V	L	V	V	W	F	-908.535
'0101010102010101030105	V	D	I	R	F	V	L	V	I	W	K	-908.534
'0101040301030101020106	V	D	V	K	L	L	L	V	A	W	L	-908.532
'0101040204020104010101	V	D	V	H	M	I	L	L	V	W	H	-908.532
'0301040102010101070110	L	D	V	R	F	V	L	V	T	W	R	-908.53
'0101040302010101070103	V	D	V	K	F	V	L	V	T	W	F	-908.529
'0201010103040101010101	I	D	I	R	I	M	L	V	V	W	H	-908.527
'0101010102030201010112	V	D	I	R	F	L	A	V	V	W	V	-908.527
'0101040405030101060101	V	D	V	Q	V	L	L	V	S	W	H	-908.525
'0201010402040101020101	I	D	I	Q	F	M	L	V	A	W	H	-908.525
'0101010304040101010104	V	D	I	K	M	M	L	V	V	W	I	-908.517
'0101040405040103010101	V	D	V	Q	V	M	L	I	V	W	H	-908.516
'0301010102040101070106	L	D	I	R	F	M	L	V	T	W	L	-908.509
'0101010203020401070101	V	D	I	H	I	I	W	V	T	W	H	-908.508
'0101020304010101040104	V	D	L	K	M	V	L	V	L	W	I	-908.507
'0101010403040101040112	V	D	I	Q	I	M	L	V	L	W	V	-908.507
'0101020102040101020111	V	D	L	R	F	M	L	V	A	W	T	-908.506
'0101030104040103010106	V	D	M	R	M	M	L	I	V	W	L	-908.506
'0101010402010104060104	V	D	I	Q	F	V	L	L	S	W	I	-908.504
'0101040102010401040110	V	D	V	R	F	V	W	V	L	W	R	-908.5
'0201040302010101010112	I	D	V	K	F	V	L	V	V	W	V	-908.5
'0201010202020301010101	I	D	I	H	F	I	V	V	V	W	H	-908.499
'0301010102020103010105	L	D	I	R	F	I	L	I	V	W	K	-908.497
'0101030103010104040102	V	D	M	R	I	V	L	L	L	W	E	-908.495
'0101040105040103040101	V	D	V	R	V	M	L	I	L	W	H	-908.493
'0101010405040101060104	V	D	I	Q	V	M	L	V	S	W	I	-908.493
'0201010103010103010110	I	D	I	R	I	V	L	I	V	W	R	-908.49
'0101040101020103020106	V	D	V	R	L	I	L	I	A	W	L	-908.49
'0201040202020101010101	I	D	V	H	F	I	L	V	V	W	H	-908.489
'0201010102010105030112	I	D	I	R	F	V	L	M	I	W	V	-908.488
'0101030402010101020102	V	D	M	Q	F	V	L	V	A	W	E	-908.488
'0101040301030101040102	V	D	V	K	L	L	L	V	L	W	E	-908.487
'0201030102010105010101	I	D	M	R	F	V	L	M	V	W	H	-908.486

'0101020202040101070101	V	D	L	H	F	M	L	V	T	W	H	-908.486
'0101010402040101070103	V	D	I	Q	F	M	L	V	T	W	F	-908.485
'0101020403010103010110	V	D	L	Q	I	V	L	I	V	W	R	-908.48
'0101040103010203010106	V	D	V	R	I	V	A	I	V	W	L	-908.48
'0101020105030101050101	V	D	L	R	V	L	L	V	M	W	H	-908.479
'0101030304010101030106	V	D	M	K	M	V	L	V	I	W	L	-908.479
'0101040103020303010101	V	D	V	R	I	I	V	I	V	W	H	-908.478
'0101030402010104010108	V	D	M	Q	F	V	L	L	V	W	N	-908.476
'0101010103020201060108	V	D	I	R	I	I	A	V	S	W	N	-908.475
'0101010102010301020105	V	D	I	R	F	V	V	V	A	W	K	-908.474
'0101030201030103040101	V	D	M	H	L	L	L	I	L	W	H	-908.472
'0201010102020401010101	I	D	I	R	F	I	W	V	V	W	H	-908.471
'0101040402010104010105	V	D	V	Q	F	V	L	L	V	W	K	-908.469
'0101040105010105020112	V	D	V	R	V	V	L	M	A	W	V	-908.466
'0101010105020101040108	V	D	I	R	V	I	L	V	L	W	N	-908.466
'0101030101020101020109	V	D	M	R	L	I	L	V	A	W	Q	-908.46
'0101030102030104070101	V	D	M	R	F	L	L	L	T	W	H	-908.459
'0101010205010101050103	V	D	I	H	V	V	L	V	M	W	F	-908.458
'0101010201030103040112	V	D	I	H	L	L	L	I	L	W	V	-908.458
'0101010103020102040110	V	D	I	R	I	I	L	A	L	W	R	-908.452
'0301040103030105010101	L	D	V	R	I	L	L	M	V	W	H	-908.452
'0101030102010103020111	V	D	M	R	F	V	L	I	A	W	T	-908.451
'0101010105030401010112	V	D	I	R	V	L	W	V	V	W	V	-908.447
'0101030101010103040102	V	D	M	R	L	V	L	I	L	W	E	-908.442
'0101010302010103060108	V	D	I	K	F	V	L	I	S	W	N	-908.441
'0101040203030105010101	V	D	V	H	I	L	L	M	V	W	H	-908.441
'0201020302030101010101	I	D	L	K	F	L	L	V	V	W	H	-908.44
'0101010104030101070109	V	D	I	R	M	L	L	V	T	W	Q	-908.439
'0101010201030201060112	V	D	I	H	L	L	A	V	S	W	V	-908.437
'0101030102010304010110	V	D	M	R	F	V	V	L	V	W	R	-908.437
'0101040104010104020109	V	D	V	R	M	V	L	L	A	W	Q	-908.436
'0101040204010105010105	V	D	V	H	M	V	L	M	V	W	K	-908.429
'0101030103010103070105	V	D	M	R	I	V	L	I	T	W	K	-908.428
'0201030102010103010108	I	D	M	R	F	V	L	I	V	W	N	-908.427
'0101040102010201020112	V	D	V	R	F	V	A	V	A	W	V	-908.426
'0101040102010204010111	V	D	V	R	F	V	A	L	V	W	T	-908.426
'0101010105030101040112	V	D	I	R	V	L	L	V	L	W	V	-908.426

**'0101010104020101070101**	V	D	I	R	M	I	L	V	T	W	H	-908.417
**'0101010303030101010105**	V	D	I	K	I	L	L	V	V	W	K	-908.414
**'0101010103030104040110**	V	D	I	R	I	L	L	L	L	W	R	-908.413
**'0101040102010201070108**	V	D	V	R	F	V	A	V	T	W	N	-908.41
**'0201010105010104070105**	I	D	I	R	V	V	L	L	T	W	K	-908.409
**'0101020201020101010109**	V	D	L	H	L	I	L	V	V	W	Q	-908.407
**'0201040101030103010106**	I	D	V	R	L	L	L	I	V	W	L	-908.405
**'0201040102020101010110**	I	D	V	R	F	I	L	V	V	W	R	-908.404
**'0101040102010104060109**	V	D	V	R	F	V	L	L	S	W	Q	-908.404
**'0101010404010103070105**	V	D	I	Q	M	V	L	I	T	W	K	-908.404
**'0101040104010103070108**	V	D	V	R	M	V	L	I	T	W	N	-908.403
**'0101020102030101050112**	V	D	L	R	F	L	L	V	M	W	V	-908.4
**'0101010101010104040106**	V	D	I	R	L	V	L	L	L	W	L	-908.396
**'0201010105010201010106**	I	D	I	R	V	V	A	V	V	W	L	-908.394
**'0301010403020101070101**	L	D	I	Q	I	I	L	V	T	W	H	-908.393
**'0101020102010103030111**	V	D	L	R	F	V	L	I	I	W	T	-908.392
**'0101010203010403010109**	V	D	I	H	I	V	W	I	V	W	Q	-908.391
**'0101030105010403010112**	V	D	M	R	V	V	W	I	V	W	V	-908.391
**'0201010105010104010109**	I	D	I	R	V	V	L	L	V	W	Q	-908.387
**'0101010105020104070106**	V	D	I	R	V	I	L	L	T	W	L	-908.386
**'0101040201020203010101**	V	D	V	H	L	I	A	I	V	W	H	-908.386
**'0101010102020203030101**	V	D	I	R	F	I	A	I	I	W	H	-908.386
**'0101020204010101070104**	V	D	L	H	M	V	L	V	T	W	I	-908.383
**'0101010102020105040104**	V	D	I	R	F	I	L	M	L	W	I	-908.383
**'0101010102010105030110**	V	D	I	R	F	V	L	M	I	W	R	-908.381
**'0101040102010401070109**	V	D	V	R	F	V	W	V	T	W	Q	-908.374
**'0101010402020401070101**	V	D	I	Q	F	I	W	V	T	W	H	-908.373
**'0101010105040101010109**	V	D	I	R	V	M	L	V	V	W	Q	-908.369
**'0201030105010101060104**	I	D	M	R	V	V	L	V	S	W	I	-908.369
**'0101040105020102040101**	V	D	V	R	V	I	L	A	L	W	H	-908.363
**'0101030202030102010101**	V	D	M	H	F	L	L	A	V	W	H	-908.361
**'0101010101010103040105**	V	D	I	R	L	V	L	I	L	W	K	-908.356
**'0301010102020102010104**	L	D	I	R	F	I	L	A	V	W	I	-908.355
**'0101040202020401010101**	V	D	V	H	F	I	W	V	V	W	H	-908.354
**'0101040402010105010112**	V	D	V	Q	F	V	L	M	V	W	V	-908.353
**'0101040102010201030110**	V	D	V	R	F	V	A	V	I	W	R	-908.351
**'0201010105040101030102**	I	D	I	R	V	M	L	V	I	W	E	-908.346

**'0101010104010103040109**	V	D	I	R	M	V	L	I	L	W	Q	-908.344
**'0201010105010105020112**	I	D	I	R	V	V	L	M	A	W	V	-908.343
**'0101030102010105040102**	V	D	M	R	F	V	L	M	L	W	E	-908.342
**'0101010102040101020102**	V	D	I	R	F	M	L	V	A	W	E	-908.337
**'0101010402020101050103**	V	D	I	Q	F	I	L	V	M	W	F	-908.336
**'0101010102020102050110**	V	D	I	R	F	I	L	A	M	W	R	-908.336
**'0201030205010103010101**	I	D	M	H	V	V	L	I	V	W	H	-908.334
**'0101040105010201030104**	V	D	V	R	V	V	A	V	I	W	I	-908.333
**'0101040102010102020104**	V	D	V	R	F	V	L	A	A	W	I	-908.33
**'0201010102010101040109**	I	D	I	R	F	V	L	V	L	W	Q	-908.328
**'0301010201020101040105**	L	D	I	H	L	I	L	V	L	W	K	-908.327
**'0201040105020101010108**	I	D	V	R	V	I	L	V	V	W	N	-908.327
**'0101010201020101020102**	V	D	I	H	L	I	L	V	A	W	E	-908.326
**'0101010303020101020112**	V	D	I	K	I	I	L	V	A	W	V	-908.321
**'0101030101020101050103**	V	D	M	R	L	I	L	V	M	W	F	-908.319
**'0201010202010102040101**	I	D	I	H	F	V	L	A	L	W	H	-908.317
**'0101040201030101070105**	V	D	V	H	L	L	L	V	T	W	K	-908.315
**'0101010105040103040108**	V	D	I	R	V	M	L	I	L	W	N	-908.315
**'0201020102010102010101**	I	D	L	R	F	V	L	A	V	W	H	-908.315
**'0101030203010101050107**	V	D	M	H	I	V	L	V	M	W	M	-908.314
**'0101010402010204010108**	V	D	I	Q	F	V	A	L	V	W	N	-908.313
**'0101030105030101050108**	V	D	M	R	V	L	L	V	M	W	N	-908.311
**'0101010103020103030102**	V	D	I	R	I	I	L	I	I	W	E	-908.304
**'0101010302010103070109**	V	D	I	K	F	V	L	I	T	W	Q	-908.303
**'0101020403030101070101**	V	D	L	Q	I	L	L	V	T	W	H	-908.301
**'0201010205010101030104**	I	D	I	H	V	V	L	V	I	W	I	-908.301
**'0101030105010105070105**	V	D	M	R	V	V	L	M	T	W	K	-908.3
**'0101010103020401050105**	V	D	I	R	I	I	W	V	M	W	K	-908.299
**'0101010303010101040105**	V	D	I	K	I	V	L	V	L	W	K	-908.292
**'0101040201020101010108**	V	D	V	H	L	I	L	V	V	W	N	-908.292
**'0101020101020103030104**	V	D	L	R	L	I	L	I	I	W	I	-908.281
**'0101010204010103030111**	V	D	I	H	M	V	L	I	I	W	T	-908.281
**'0101010103040101030102**	V	D	I	R	I	M	L	V	I	W	E	-908.279
**'0101040102010102010103**	V	D	V	R	F	V	L	A	V	W	F	-908.279
**'0101010203010104040102**	V	D	I	H	I	V	L	L	L	W	E	-908.278
**'0201010105030101050110**	I	D	I	R	V	L	L	V	M	W	R	-908.271
**'0301040205010103010101**	L	D	V	H	V	V	L	I	V	W	H	-908.27

'01010201020101010201 04	V	D	L	R	F	V	L	V	A	W	I	-908.265
'010103010101020304 0104	V	D	M	R	L	V	A	I	L	W	I	-908.263
'0101010202030301010102	V	D	I	H	F	L	V	V	V	W	E	-908.262
'0201010302040101010106	I	D	I	K	F	M	L	V	V	W	L	-908.261
'0101010201020103040111	V	D	I	H	L	I	L	I	L	W	T	-908.261
'0101010303030101070101	V	D	I	K	I	L	L	V	T	W	H	-908.259
'0101030104040101050104	V	D	M	R	M	M	L	V	M	W	I	-908.253
'0101010202010204010109	V	D	I	H	F	V	A	L	V	W	Q	-908.253
'0101010102030201030109	V	D	I	R	F	L	A	V	I	W	Q	-908.252
'0101040405040101010105	V	D	V	Q	V	M	L	V	V	W	K	-908.251
'0101040205010101020108	V	D	V	H	V	V	L	V	A	W	N	-908.25
'0101030205010101070104	V	D	M	H	V	V	L	V	T	W	I	-908.249
'0101040104020101070105	V	D	V	R	M	I	L	V	T	W	K	-908.248
'0201010102020101060111	I	D	I	R	F	I	L	V	S	W	T	-908.248
'0101010101030201040105	V	D	I	R	L	L	A	V	L	W	K	-908.247
'0101010102030104060111	V	D	I	R	F	L	L	L	S	W	T	-908.247
'0101010401010101040104	V	D	I	Q	L	V	L	V	L	W	I	-908.245
'0301010302010102010112	L	D	I	K	F	V	L	A	V	W	V	-908.244
'0101010102030103050107	V	D	I	R	F	L	L	I	M	W	M	-908.243
'0101010403030101020101	V	D	I	Q	I	L	L	V	A	W	H	-908.24
'0201010202010103050101	I	D	I	H	F	V	L	I	M	W	H	-908.238
'0101010102020201050107	V	D	I	R	F	I	A	V	M	W	M	-908.234
'0101040103020101050102	V	D	V	R	I	I	L	V	M	W	E	-908.233
'0101010201040101040112	V	D	I	H	L	M	L	V	L	W	V	-908.233
'0101030103040201010105	V	D	M	R	I	M	A	V	V	W	K	-908.232
'0101040103010104050109	V	D	V	R	I	V	L	L	M	W	Q	-908.231
'0101010103020301040111	V	D	I	R	I	I	V	V	L	W	T	-908.231
'0101010204020101010104	V	D	I	H	M	I	L	V	V	W	I	-908.23
'0301010203010104010112	L	D	I	H	I	V	L	L	V	W	V	-908.228
'0301010102010103010109	L	D	I	R	F	V	L	I	V	W	Q	-908.223
'0201030105030101060101	I	D	M	R	V	L	L	V	S	W	H	-908.219
'0201030403010101010105	I	D	M	Q	I	V	L	V	V	W	K	-908.218
'0301010202040101010102	L	D	I	H	F	M	L	V	V	W	E	-908.217
'0301010103020103010110	L	D	I	R	I	I	L	I	V	W	R	-908.216
'0101040102020101050107	V	D	V	R	F	I	L	V	M	W	M	-908.216
'0201010104030201010104	I	D	I	R	M	L	A	V	V	W	I	-908.215
'0101010105020104030111	V	D	I	R	V	I	L	L	I	W	T	-908.214

**'0301010102010104040112**	L	D	I	R	F	V	L	L	L	W	V	-908.211
**'0101030102030301010110**	V	D	M	R	F	L	V	V	V	W	R	-908.211
**'0101010305020101010101**	V	D	I	K	V	I	L	V	V	W	H	-908.209
**'0101010203020201010107**	V	D	I	H	I	I	A	V	V	W	M	-908.208
**'0101030403030201010101**	V	D	M	Q	I	L	A	V	V	W	H	-908.205
**'0101040402010101040111**	V	D	V	Q	F	V	L	V	L	W	T	-908.205
**'0101010102020301050110**	V	D	I	R	F	I	V	V	M	W	R	-908.204
**'0101010302010101020110**	V	D	I	K	F	V	L	V	A	W	R	-908.204
**'0101040304010101030104**	V	D	V	K	M	V	L	V	I	W	I	-908.203
**'0101040101020103070112**	V	D	V	R	L	I	L	I	T	W	V	-908.202
**'0101010102030303010106**	V	D	I	R	F	L	V	I	V	W	L	-908.201
**'0101020201020101040110**	V	D	L	H	L	I	L	V	L	W	R	-908.199
**'0201010102040102010103**	I	D	I	R	F	M	L	A	V	W	F	-908.199
**'0101010104040101060108**	V	D	I	R	M	M	L	V	S	W	N	-908.196
**'0101040301020103010110**	V	D	V	K	L	I	L	I	V	W	R	-908.191
**'0101040201030101050103**	V	D	V	H	L	L	L	V	M	W	F	-908.189
**'0101020201030101070112**	V	D	L	H	L	L	L	V	T	W	V	-908.189
**'0201010405040101010112**	I	D	I	Q	V	M	L	V	V	W	V	-908.188
**'0101030205010105070101**	V	D	M	H	V	V	L	M	T	W	H	-908.188
**'0201040101030101030104**	I	D	V	R	L	L	L	V	I	W	I	-908.187
**'0201010305030101010107**	I	D	I	K	V	L	L	V	V	W	M	-908.186
**'0101040103010401010106**	V	D	V	R	I	V	W	V	V	W	L	-908.186
**'0301010205010103010112**	L	D	I	H	V	V	L	I	V	W	V	-908.185
**'0101010103010203040106**	V	D	I	R	I	V	A	I	L	W	L	-908.184
**'0101030104030101030104**	V	D	M	R	M	L	L	V	I	W	I	-908.183
**'0101010205030403010101**	V	D	I	H	V	L	W	I	V	W	H	-908.183
**'0101010103020102030112**	V	D	I	R	I	I	L	A	I	W	V	-908.177
**'0101020103010103030110**	V	D	L	R	I	V	L	I	I	W	R	-908.177
**'0201010105010101040112**	I	D	I	R	V	V	L	V	L	W	V	-908.174
**'0101010202030201010103**	V	D	I	H	F	L	A	V	V	W	F	-908.172
**'0101010305040101030101**	V	D	I	K	V	M	L	V	I	W	H	-908.17
**'0201010102030401010104**	I	D	I	R	F	L	W	V	V	W	I	-908.168
**'0101010301020101040104**	V	D	I	K	L	I	L	V	L	W	I	-908.165
**'0101040402010105040101**	V	D	V	Q	F	V	L	M	L	W	H	-908.165
**'0101010105010203040105**	V	D	I	R	V	V	A	I	L	W	K	-908.165
**'0101020105010103030105**	V	D	L	R	V	V	L	I	I	W	K	-908.165
**'0101010203010101010107**	V	D	I	H	I	V	L	V	V	W	M	-908.165

**'0101010105030104030106**	V	D	I	R	V	L	L	L	I	W	L	-908.164
**'0101010105010204030104**	V	D	I	R	V	V	A	L	I	W	I	-908.163
**'0101030105020201010107**	V	D	M	R	V	I	A	V	V	W	M	-908.162
**'0301010205010105010105**	L	D	I	H	V	V	L	M	V	W	K	-908.161
**'0301010102020101050106**	L	D	I	R	F	I	L	V	M	W	L	-908.16
**'0101030101020103040106**	V	D	M	R	L	I	L	I	L	W	L	-908.16
**'0101010203010104030101**	V	D	I	H	I	V	L	L	I	W	H	-908.159
**'0101040402010101030105**	V	D	V	Q	F	V	L	V	I	W	K	-908.159
**'0101030103010403010108**	V	D	M	R	I	V	W	I	V	W	N	-908.159
**'0101020302010101040108**	V	D	L	K	F	V	L	V	L	W	N	-908.157
**'0101030102030301040101**	V	D	M	R	F	L	V	V	L	W	H	-908.154
**'0201010101030103070112**	I	D	I	R	L	L	L	I	T	W	V	-908.152
**'0101030102010403010105**	V	D	M	R	F	V	W	I	V	W	K	-908.151
**'0101010403030101010102**	V	D	I	Q	I	L	L	V	V	W	E	-908.151
**'0101020105010104040112**	V	D	L	R	V	V	L	L	L	W	V	-908.149
**'0201010105020104010105**	I	D	I	R	V	I	L	L	V	W	K	-908.149
**'0101010402040101040108**	V	D	I	Q	F	M	L	V	L	W	N	-908.147
**'0301030205010101010110**	L	D	M	H	V	V	L	V	V	W	R	-908.144
**'0101030104030101040105**	V	D	M	R	M	L	L	V	L	W	K	-908.144
**'0101040105010403070101**	V	D	V	R	V	V	W	I	T	W	H	-908.143
**'0301040105010103010102**	L	D	V	R	V	V	L	I	V	W	E	-908.141
**'0201010401030101010101**	I	D	I	Q	L	L	L	V	V	W	H	-908.14
**'0101040102010104070103**	V	D	V	R	F	V	L	L	T	W	F	-908.14
**'0201040104040101010107**	I	D	V	R	M	M	L	V	V	W	M	-908.138
**'0101040201020103050101**	V	D	V	H	L	I	L	I	M	W	H	-908.137
**'0101010102010105070111**	V	D	I	R	F	V	L	M	T	W	T	-908.135
**'0101010103010104030110**	V	D	I	R	I	V	L	L	I	W	R	-908.134
**'0101010102010304040112**	V	D	I	R	F	V	V	L	L	W	V	-908.13
**'0101010402030101060108**	V	D	I	Q	F	L	L	V	S	W	N	-908.127
**'0101040102010203010104**	V	D	V	R	F	V	A	I	V	W	I	-908.127
**'0101030105010105010108**	V	D	M	R	V	V	L	M	V	W	N	-908.126
**'0101010203040103040101**	V	D	I	H	I	M	L	I	L	W	H	-908.124
**'0101010405030101030101**	V	D	I	Q	V	L	L	V	I	W	H	-908.123
**'0101030101020101070101**	V	D	M	R	L	I	L	V	T	W	H	-908.123
**'0301020102010104010106**	L	D	L	R	F	V	L	L	V	W	L	-908.12
**'0201040305010101070101**	I	D	V	K	V	V	L	V	T	W	H	-908.12
**'0101010403030401010112**	V	D	I	Q	I	L	W	V	V	W	V	-908.12

**'0201040103010105010107**	I	D	V	R	I	V	L	M	V	W	M	-908.119
**'0201040101030101010105**	I	D	V	R	L	L	L	V	V	W	K	-908.118
**'0101010305020101040102**	V	D	I	K	V	I	L	V	L	W	E	-908.118
**'0101020104010103020108**	V	D	L	R	M	V	L	I	A	W	N	-908.113
**'0101010304010103030105**	V	D	I	K	M	V	L	I	I	W	K	-908.112
**'0101040104010104040108**	V	D	V	R	M	V	L	L	L	W	N	-908.11
**'0101020201010103040104**	V	D	L	H	L	V	L	I	L	W	I	-908.11
**'0301010102010102010108**	L	D	I	R	F	V	L	A	V	W	N	-908.109
**'0101010203010103010108**	V	D	I	H	I	V	L	I	V	W	N	-908.107
**'0101010102010205040106**	V	D	I	R	F	V	A	M	L	W	L	-908.106
**'0201040101040101070106**	I	D	V	R	L	M	L	V	T	W	L	-908.106
**'0301010105020202010101**	L	D	I	R	V	I	A	A	V	W	H	-908.105
**'0101020105010103020111**	V	D	L	R	V	V	L	I	A	W	T	-908.102
**'0101010202020301010107**	V	D	I	H	F	I	V	V	V	W	M	-908.101
**'0101040402010101010103**	V	D	V	Q	F	V	L	V	V	W	F	-908.1
**'0201030105010201010104**	I	D	M	R	V	V	A	V	V	W	I	-908.1
**'0101010104020101010108**	V	D	I	R	M	I	L	V	V	W	N	-908.093
**'0101040103040102070101**	V	D	V	R	I	M	L	A	T	W	H	-908.089
**'0101030402040101020101**	V	D	M	Q	F	M	L	V	A	W	H	-908.088
**'0201040102020201010101**	I	D	V	R	F	I	A	V	V	W	H	-908.086
**'0101020104020103010103**	V	D	L	R	M	I	L	I	V	W	F	-908.085
**'0101010205010101040108**	V	D	I	H	V	V	L	V	L	W	N	-908.084
**'0101010405040101030112**	V	D	I	Q	V	M	L	V	I	W	V	-908.082
**'0101010105010103050106**	V	D	I	R	V	V	L	I	M	W	L	-908.08
**'0101030105030201040101**	V	D	M	R	V	L	A	V	L	W	H	-908.079
**'0101040101020104010108**	V	D	V	R	L	I	L	L	V	W	N	-908.079
**'0101040303010101040102**	V	D	V	K	I	V	L	V	L	W	E	-908.079
**'0301010405010101010101**	L	D	I	Q	V	V	L	V	V	W	H	-908.074
**'0101010102010404070106**	V	D	I	R	F	V	W	L	T	W	L	-908.072
**'0201010103010105040112**	I	D	I	R	I	V	L	M	L	W	V	-908.071
**'0101040102010105020107**	V	D	V	R	F	V	L	M	A	W	M	-908.069
**'0201010102040101050107**	I	D	I	R	F	M	L	V	M	W	M	-908.068
**'0101040101030101010103**	V	D	V	R	L	L	L	V	V	W	F	-908.068
**'0101010205040101020112**	V	D	I	H	V	M	L	V	A	W	V	-908.063
**'0101010203020102040101**	V	D	I	H	I	I	L	A	L	W	H	-908.063
**'0201030102030101030101**	I	D	M	R	F	L	L	V	I	W	H	-908.061
**'0201010203010103010105**	I	D	I	H	I	V	L	I	V	W	K	-908.059

'0101030105010104060105	V	D	M	R	V	V	L	L	S	W	K	-908.058
'0101010102010104020111	V	D	I	R	F	V	L	L	A	W	T	-908.058
'0101020103030101050105	V	D	L	R	I	L	L	V	M	W	K	-908.055
'0301030102010101010105	L	D	M	R	F	V	L	V	V	W	K	-908.054
'0101030403010101050110	V	D	M	Q	I	V	L	V	M	W	R	-908.052
'0301040105010101070107	L	D	V	R	V	V	L	V	T	W	M	-908.052
'0101020202010101010111	V	D	L	H	F	V	L	V	V	W	T	-908.047
'0101040203010101040112	V	D	V	H	I	V	L	V	L	W	V	-908.046
'0101030101020201040112	V	D	M	R	L	I	A	V	L	W	V	-908.046
'0101020102020301040101	V	D	L	R	F	I	V	V	L	W	H	-908.046
'0101010103010105040110	V	D	I	R	I	V	L	M	L	W	R	-908.043
'0101030105010102050106	V	D	M	R	V	V	L	A	M	W	L	-908.041
'0301010102010104070111	L	D	I	R	F	V	L	L	T	W	T	-908.039
'0101010202020401020101	V	D	I	H	F	I	W	V	A	W	H	-908.038
'0101010302010203060101	V	D	I	K	F	V	A	I	S	W	H	-908.035
'0301030201020101010106	L	D	M	H	L	I	L	V	V	W	L	-908.034
'0101030401020101040112	V	D	M	Q	L	I	L	V	L	W	V	-908.029
'0301010202020101070101	L	D	I	H	F	I	L	V	T	W	H	-908.028
'0101010104030103040105	V	D	I	R	M	L	L	I	L	W	K	-908.026
'0301010102030105010105	L	D	I	R	F	L	L	M	V	W	K	-908.023
'0101040205010301010104	V	D	V	H	V	V	V	V	V	W	I	-908.023
'0101010102030102030112	V	D	I	R	F	L	L	A	I	W	V	-908.023
'0101040402010201040101	V	D	V	Q	F	V	A	V	L	W	H	-908.019
'0201030103010103020101	I	D	M	R	I	V	L	I	A	W	H	-908.016
'0101040103040102010112	V	D	V	R	I	M	L	A	V	W	V	-908.014
'0101030105030102070101	V	D	M	R	V	L	L	A	T	W	H	-908.014
'0101010103040101020108	V	D	I	R	I	M	L	V	A	W	N	-908.014
'0301040105010101060105	L	D	V	R	V	V	L	V	S	W	K	-908.013
'0301010102010101050110	L	D	I	R	F	V	L	V	M	W	R	-908.013
'0101010402030301070101	V	D	I	Q	F	L	V	V	T	W	H	-908.013
'0201030103030101070101	I	D	M	R	I	L	L	V	T	W	H	-908.012
'0201010205010104010112	I	D	I	H	V	V	L	L	V	W	V	-908.011
'0201030202010101010110	I	D	M	H	F	V	L	V	V	W	R	-908.011
'0201010102010301040110	I	D	I	R	F	V	V	V	L	W	R	-908.011
'0101030205010104060101	V	D	M	H	V	V	L	L	S	W	H	-908.01
'0101010404030101040111	V	D	I	Q	M	L	L	V	L	W	T	-908.009
'0101010402030301010106	V	D	I	Q	F	L	V	V	V	W	L	-908.007

**'0201010305030101050101**	I	D	I	K	V	L	L	V	M	W	H	-908.003
**'0101040401020103070101**	V	D	V	Q	L	I	L	I	T	W	H	-907.999
**'0101010103040103040112**	V	D	I	R	I	M	L	I	L	W	V	-907.997
**'0101040104010304010101**	V	D	V	R	M	V	V	L	V	W	H	-907.997
**'0201010203030101070101**	I	D	I	H	I	L	L	V	T	W	H	-907.996
**'0301030103010101020104**	L	D	M	R	I	V	L	V	A	W	I	-907.995
**'0101030104040101040102**	V	D	M	R	M	M	L	V	L	W	E	-907.994
**'0101010405010101030106**	V	D	I	Q	V	V	L	V	I	W	L	-907.992
**'0301030105010104010110**	L	D	M	R	V	V	L	L	V	W	R	-907.991
**'0101010202030201030101**	V	D	I	H	F	L	A	V	I	W	H	-907.99
**'0101030102010201020102**	V	D	M	R	F	V	A	V	A	W	E	-907.99
**'0101040104010104010110**	V	D	V	R	M	V	L	L	V	W	R	-907.99
**'0101010405010103040109**	V	D	I	Q	V	V	L	I	L	W	Q	-907.986
**'0101030105010103070112**	V	D	M	R	V	V	L	I	T	W	V	-907.982
**'0101010105010104060112**	V	D	I	R	V	V	L	L	S	W	V	-907.982
**'0101030103020301020101**	V	D	M	R	I	I	V	V	A	W	H	-907.981
**'0101020201010103070101**	V	D	L	H	L	V	L	I	T	W	H	-907.981
**'0101040105010101010109**	V	D	V	R	V	V	L	V	V	W	Q	-907.98
**'0101030202010105010108**	V	D	M	H	F	V	L	M	V	W	N	-907.979
**'0101010201010103030109**	V	D	I	H	L	V	L	I	I	W	Q	-907.975
**'0201040203010101060101**	I	D	V	H	I	V	L	V	S	W	H	-907.974
**'0101010105020301010109**	V	D	I	R	V	I	V	V	V	W	Q	-907.968
**'0301010205010101040111**	L	D	I	H	V	V	L	V	L	W	T	-907.964
**'0301010105020401010105**	L	D	I	R	V	I	W	V	V	W	K	-907.961
**'0101010405010104030104**	V	D	I	Q	V	V	L	L	I	W	I	-907.961
**'0101010405020104010107**	V	D	I	Q	V	I	L	L	V	W	M	-907.958
**'0201030104010101040110**	I	D	M	R	M	V	L	V	L	W	R	-907.958
**'0201010104020101040104**	I	D	I	R	M	I	L	V	L	W	I	-907.958
**'0101010102020105070105**	V	D	I	R	F	I	L	M	T	W	K	-907.957
**'0101030203010403010101**	V	D	M	H	I	V	W	I	V	W	H	-907.955
**'0301010303020101010104**	L	D	I	K	I	I	L	V	V	W	I	-907.954
**'0101010304020101050105**	V	D	I	K	M	I	L	V	M	W	K	-907.952
**'0201010104010105040105**	I	D	I	R	M	V	L	M	L	W	K	-907.95
**'0101010202030101020102**	V	D	I	H	F	L	L	V	A	W	E	-907.949
**'0101010203010101070112**	V	D	I	H	I	V	L	V	T	W	V	-907.948
**'0101030302010105010101**	V	D	M	K	F	V	L	M	V	W	H	-907.947
**'0101010102030202010106**	V	D	I	R	F	L	A	A	V	W	L	-907.942

**'01010104030201010701 12**	V	D	I	Q	I	I	L	V	T	W	V	-907.941
**'0101010103020201010104**	V	D	I	R	I	I	A	V	V	W	I	-907.939
**'0101010104030101010105**	V	D	I	R	M	L	L	V	V	W	K	-907.939
**'0101030303010101070102**	V	D	M	K	I	V	L	V	T	W	E	-907.937
**'0201030105040101010112**	I	D	M	R	V	M	L	V	V	W	V	-907.935
**'0101030103010102040110**	V	D	M	R	I	V	L	A	L	W	R	-907.935
**'0101040105030105010104**	V	D	V	R	V	L	L	M	V	W	I	-907.931
**'0101030402010101030106**	V	D	M	Q	F	V	L	V	I	W	L	-907.929
**'0101020102020103030101**	V	D	L	R	F	I	L	I	I	W	H	-907.928
**'0101010201040103040106**	V	D	I	H	L	M	L	I	L	W	L	-907.928
**'0101040303030104010101**	V	D	V	K	I	L	L	L	V	W	H	-907.928
**'0201030201020101010103**	I	D	M	H	L	I	L	V	V	W	F	-907.927
**'0101030204030101010103**	V	D	M	H	M	L	L	V	V	W	F	-907.926
**'0101010302030301010108**	V	D	I	K	F	L	V	V	V	W	N	-907.925
**'0101040103010105030108**	V	D	V	R	I	V	L	M	I	W	N	-907.924
**'0101030103030102010108**	V	D	M	R	I	L	L	A	V	W	N	-907.922
**'0201010102020101050102**	I	D	I	R	F	I	L	V	M	W	E	-907.922
**'0301010102010102030110**	L	D	I	R	F	V	L	A	I	W	R	-907.921
**'0201010105010203010102**	I	D	I	R	V	V	A	I	V	W	E	-907.92
**'0101040404030101010108**	V	D	V	Q	M	L	L	V	V	W	N	-907.919
**'0101010103020201070101**	V	D	I	R	I	I	A	V	T	W	H	-907.918
**'0301030102040101010112**	L	D	M	R	F	M	L	V	V	W	V	-907.917
**'0101010302010104070101**	V	D	I	K	F	V	L	L	T	W	H	-907.916
**'0201010103010303010105**	I	D	I	R	I	V	V	I	V	W	K	-907.914
**'0101020101030101040108**	V	D	L	R	L	L	L	V	L	W	N	-907.914
**'0101030105010303040101**	V	D	M	R	V	V	V	I	L	W	H	-907.914
**'0101010202010303070101**	V	D	I	H	F	V	V	I	T	W	H	-907.913
**'0301010202010102010111**	L	D	I	H	F	V	L	A	V	W	T	-907.912
**'0101010104030104020111**	V	D	I	R	M	L	L	L	A	W	T	-907.912
**'0101040201010105040106**	V	D	V	H	L	V	L	M	L	W	L	-907.91
**'0201010101030102040112**	I	D	I	R	L	L	L	A	L	W	V	-907.909
**'0101010102010404010112**	V	D	I	R	F	V	W	L	V	W	V	-907.908
**'0101010203020101060105**	V	D	I	H	I	I	L	V	S	W	K	-907.906
**'0101030403010201010104**	V	D	M	Q	I	V	A	V	V	W	I	-907.904
**'0101010105030201030101**	V	D	I	R	V	L	A	V	I	W	H	-907.9
**'0301030205010104010101**	L	D	M	H	V	V	L	L	V	W	H	-907.9
**'0101040405040101040101**	V	D	V	Q	V	M	L	V	L	W	H	-907.899

**'0101010102020103050108**	V	D	I	R	F	I	L	I	M	W	N	-907.898
**'0101040204010101050105**	V	D	V	H	M	V	L	V	M	W	K	-907.895
**'0101030104020101010104**	V	D	M	R	M	I	L	V	V	W	I	-907.893
**'0301040105010101030104**	L	D	V	R	V	V	L	V	I	W	I	-907.889
**'0201010202010101030105**	I	D	I	H	F	V	L	V	I	W	K	-907.887
**'0201040103010102010110**	I	D	V	R	I	V	L	A	V	W	R	-907.886
**'0101010105010201050109**	V	D	I	R	V	V	A	V	M	W	Q	-907.883
**'0201030105030101010107**	I	D	M	R	V	L	L	V	V	W	M	-907.88
**'0201010103020102010105**	I	D	I	R	I	I	L	A	V	W	K	-907.877
**'0101030201010103030112**	V	D	M	H	L	V	L	I	I	W	V	-907.875
**'0201010103010101010112**	I	D	I	R	I	V	L	V	V	W	V	-907.874
**'0201020105020101010103**	I	D	L	R	V	I	L	V	V	W	F	-907.873
**'0101040102040101040109**	V	D	V	R	F	M	L	V	L	W	Q	-907.873
**'0101020102020101060107**	V	D	L	R	F	I	L	V	S	W	M	-907.873
**'0201030201020101040101**	I	D	M	H	L	I	L	V	L	W	H	-907.871
**'0101010102010102070105**	V	D	I	R	F	V	L	A	T	W	K	-907.869
**'0201030105010104010112**	I	D	M	R	V	V	L	L	V	W	V	-907.869
**'0101010105010201070101**	V	D	I	R	V	V	A	V	T	W	H	-907.869
**'0301010202010103010105**	L	D	I	H	F	V	L	I	V	W	K	-907.869
**'0101010105020101060103**	V	D	I	R	V	I	L	V	S	W	F	-907.868
**'0101010104020101040110**	V	D	I	R	M	I	L	V	L	W	R	-907.868
**'0101010102020402010110**	V	D	I	R	F	I	W	A	V	W	R	-907.868
**'0101010404020101030104**	V	D	I	Q	M	I	L	V	I	W	I	-907.868
**'0101010203030101040103**	V	D	I	H	I	L	L	V	L	W	F	-907.867
**'0101010103030301030108**	V	D	I	R	I	L	V	V	I	W	N	-907.866
**'0101040301030101050105**	V	D	V	K	L	L	L	V	M	W	K	-907.862
**'0201010102020202010101**	I	D	I	R	F	I	A	A	V	W	H	-907.862
**'0101010104040103040106**	V	D	I	R	M	M	L	I	L	W	L	-907.859
**'0101030404040101010110**	V	D	M	Q	M	M	L	V	V	W	R	-907.859
**'0201030405010101010112**	I	D	M	Q	V	V	L	V	V	W	V	-907.859
**'0201010202040103010101**	I	D	I	H	F	M	L	I	V	W	H	-907.855
**'0201040101020103010108**	I	D	V	R	L	I	L	I	V	W	N	-907.854
**'0101010103010304040105**	V	D	I	R	I	V	V	L	L	W	K	-907.854
**'0101010103020103050106**	V	D	I	R	I	I	L	I	M	W	L	-907.851
**'0101010103020203010105**	V	D	I	R	I	I	A	I	V	W	K	-907.851
**'0101010102030403010112**	V	D	I	R	F	L	W	I	V	W	V	-907.849
**'0101010203010102040108**	V	D	I	H	I	V	L	A	L	W	N	-907.848

'0101010103010203010110	V	D	I	R	I	V	A	I	V	W	R	-907.848
'0101010302040102020101	V	D	I	K	F	M	L	A	A	W	H	-907.847
'0101030302010101070111	V	D	M	K	F	V	L	V	T	W	T	-907.847
'0101020102020103010111	V	D	L	R	F	I	L	I	V	W	T	-907.845
'0101020105020201010112	V	D	L	R	V	I	A	V	V	W	V	-907.841
'0101040102010101060104	V	D	V	R	F	V	L	V	S	W	I	-907.84
'0301010103020101070109	L	D	I	R	I	I	L	V	T	W	Q	-907.838
'0101030102010105070108	V	D	M	R	F	V	L	M	T	W	N	-907.838
'0301010105020103030101	L	D	I	R	V	I	L	I	I	W	H	-907.835
'0201010103010105070108	I	D	I	R	I	V	L	M	T	W	N	-907.833
'0301010103020104020101	L	D	I	R	I	I	L	L	A	W	H	-907.833
'0101030102010101010110	V	D	M	R	F	V	L	V	V	W	R	-907.829
'0101020103020101070102	V	D	L	R	I	I	L	V	T	W	E	-907.829
'0201010105010102030105	I	D	I	R	V	V	L	A	I	W	K	-907.828
'0101010103020203060101	V	D	I	R	I	I	A	I	S	W	H	-907.825
'0301040103010101050101	L	D	V	R	I	V	L	V	M	W	H	-907.824
'0101040104030103010110	V	D	V	R	M	L	L	I	V	W	R	-907.824
'0101010102030104020105	V	D	I	R	F	L	L	L	A	W	K	-907.82
'0301010105010103040104	L	D	I	R	V	V	L	I	L	W	I	-907.819
'0201010102040101020108	I	D	I	R	F	M	L	V	A	W	N	-907.819
'0101040102010204050101	V	D	V	R	F	V	A	L	M	W	H	-907.818
'0101030302030401010101	V	D	M	K	F	L	W	V	V	W	H	-907.815
'0101040201020101020111	V	D	V	H	L	I	L	V	A	W	T	-907.814
'0301010105030102010101	L	D	I	R	V	L	L	A	V	W	H	-907.814
'0101030304040101040101	V	D	M	K	M	M	L	V	L	W	H	-907.813
'0101040204010101020107	V	D	V	H	M	V	L	V	A	W	M	-907.809
'0101010102030104070107	V	D	I	R	F	L	L	L	T	W	M	-907.809
'0101010403010101030104	V	D	I	Q	I	V	L	V	I	W	I	-907.806
'0101010102020301030106	V	D	I	R	F	I	V	V	I	W	L	-907.806
'0101010203030401010102	V	D	I	H	I	L	W	V	V	W	E	-907.806
'0101010203010104060110	V	D	I	H	I	V	L	L	S	W	R	-907.805
'0101010204010403010104	V	D	I	H	M	V	W	I	V	W	I	-907.801
'0101040203010401010104	V	D	V	H	I	V	W	V	V	W	I	-907.8
'0101040101020104020105	V	D	V	R	L	I	L	L	A	W	K	-907.799
'0301040201020101010111	L	D	V	H	L	I	L	V	V	W	T	-907.799
'0101030102010201010108	V	D	M	R	F	V	A	V	V	W	N	-907.797
'0101010401020101040109	V	D	I	Q	L	I	L	V	L	W	Q	-907.797

'0101010102040201030112	V	D	I	R	F	M	A	V	I	W	V	-907.796
'0101010403010103010103	V	D	I	Q	I	V	L	I	V	W	F	-907.796
'0101010403020101050108	V	D	I	Q	I	I	L	V	M	W	N	-907.795
'0101010105040102020101	V	D	I	R	V	M	L	A	A	W	H	-907.794
'0101030202010301070101	V	D	M	H	F	V	V	V	T	W	H	-907.793
'0301040105030101010106	L	D	V	R	V	L	L	V	V	W	L	-907.792
'0101010203010204010105	V	D	I	H	I	V	A	L	V	W	K	-907.792
'0301040104010101040110	L	D	V	R	M	V	L	V	L	W	R	-907.788
'0301040103040101010101	L	D	V	R	I	M	L	V	V	W	H	-907.788
'0301010302010104010106	L	D	I	K	F	V	L	L	V	W	L	-907.787
'0101030402010101070109	V	D	M	Q	F	V	L	V	T	W	Q	-907.787
'0101030102010103060110	V	D	M	R	F	V	L	I	S	W	R	-907.786
'0301040102010105010112	L	D	V	R	F	V	L	M	V	W	V	-907.786
'0201040105010104020101	I	D	V	R	V	V	L	L	A	W	H	-907.786
'0101020101030101010111	V	D	L	R	L	L	L	V	V	W	T	-907.784
'0101010105030304010104	V	D	I	R	V	L	V	L	V	W	I	-907.783
'0101040104020105010102	V	D	V	R	M	I	L	M	V	W	E	-907.783
'0101030103010303010104	V	D	M	R	I	V	V	I	V	W	I	-907.783
'0201010103020101060105	I	D	I	R	I	I	L	V	S	W	K	-907.782
'0101040204040101010112	V	D	V	H	M	M	L	V	V	W	V	-907.782
'0101010103040301040101	V	D	I	R	I	M	V	V	L	W	H	-907.781
'0101020102030103070101	V	D	L	R	F	L	L	I	T	W	H	-907.779
'0201010103020101040108	I	D	I	R	I	I	L	V	L	W	N	-907.778
'0101010103010104050108	V	D	I	R	I	V	L	L	M	W	N	-907.776
'0101040302010102010110	V	D	V	K	F	V	L	A	V	W	R	-907.774
'0101010103040201040105	V	D	I	R	I	M	A	V	L	W	K	-907.773
'0101010204010101010105	V	D	I	H	M	V	L	V	V	W	K	-907.773
'0301040403010101010105	L	D	V	Q	I	V	L	V	V	W	K	-907.77
'0101010204010103020105	V	D	I	H	M	V	L	I	A	W	K	-907.769
'0101010204020103010105	V	D	I	H	M	I	L	I	V	W	K	-907.768
'0301040103010101070112	L	D	V	R	I	V	L	V	T	W	V	-907.767
'0101010103030101020110	V	D	I	R	I	L	L	V	A	W	R	-907.765
'0101030302010101030110	V	D	M	K	F	V	L	V	I	W	R	-907.764
'0101010302020105010109	V	D	I	K	F	I	L	M	V	W	Q	-907.763
'0101010103010403040105	V	D	I	R	I	V	W	I	L	W	K	-907.763
'0101010202010203010105	V	D	I	H	F	V	A	I	V	W	K	-907.762
'0301020105010101020104	L	D	L	R	V	V	L	V	A	W	I	-907.761

'0201010101030301040105	I	D	I	R	L	L	V	V	L	W	K	-907.761
'0101040204020101010106	V	D	V	H	M	I	L	V	V	W	L	-907.76
'0101040403020101060101	V	D	V	Q	I	I	L	V	S	W	H	-907.76
'0101030102010104030102	V	D	M	R	F	V	L	L	I	W	E	-907.759
'0101010102030301050109	V	D	I	R	F	L	V	V	M	W	Q	-907.758
'0101040404010104010103	V	D	V	Q	M	V	L	L	V	W	F	-907.757
'0101020105030101020108	V	D	L	R	V	L	L	V	A	W	N	-907.757
'0101030103040103010109	V	D	M	R	I	M	L	I	V	W	Q	-907.756
'0301010102010101020104	L	D	I	R	F	V	L	V	A	W	I	-907.755
'0101030103030102040101	V	D	M	R	I	L	L	A	L	W	H	-907.755
'0201010304010105010106	I	D	I	K	M	V	L	M	V	W	L	-907.754
'0101040104010104060101	V	D	V	R	M	V	L	L	S	W	H	-907.753
'0101040103010101010112	V	D	V	R	I	V	L	V	V	W	V	-907.747
'0101010203030304010101	V	D	I	H	I	L	V	L	V	W	H	-907.747
'0101040105010401010108	V	D	V	R	V	V	W	V	V	W	N	-907.746
'0101010104010104040110	V	D	I	R	M	V	L	L	L	W	R	-907.744
'0101040101010104010109	V	D	V	R	L	V	L	L	V	W	Q	-907.742
'0201010105040101050108	I	D	I	R	V	M	L	V	M	W	N	-907.739
'0201030102040101040101	I	D	M	R	F	M	L	V	L	W	H	-907.739
'0301010402010104010112	L	D	I	Q	F	V	L	L	V	W	V	-907.739
'0101010102030104050108	V	D	I	R	F	L	L	L	M	W	N	-907.738
'0101030105010104050112	V	D	M	R	V	V	L	L	M	W	V	-907.733
'0101010103020403040101	V	D	I	R	I	I	W	I	L	W	H	-907.731
'0101010102010403030106	V	D	I	R	F	V	W	I	I	W	L	-907.729
'0101040103040101070105	V	D	V	R	I	M	L	V	T	W	K	-907.729
'0101020101020103040108	V	D	L	R	L	I	L	I	L	W	N	-907.728
'0101010105010104010103	V	D	I	R	V	V	L	L	V	W	F	-907.727
'0201040103030102010101	I	D	V	R	I	L	L	A	V	W	H	-907.727
'0101030103040101010102	V	D	M	R	I	M	L	V	V	W	E	-907.725
'0101020102030303010101	V	D	L	R	F	L	V	I	V	W	H	-907.725
'0301030102020102010101	L	D	M	R	F	I	L	A	V	W	H	-907.722
'0201030102010303010101	I	D	M	R	F	V	V	I	V	W	H	-907.719
'0101040302010203010101	V	D	V	K	F	V	A	I	V	W	H	-907.719
'0101040101010101030106	V	D	V	R	L	V	L	V	I	W	L	-907.715
'0101030402010204010101	V	D	M	Q	F	V	A	L	V	W	H	-907.714
'0101010302020203010101	V	D	I	K	F	I	A	I	V	W	H	-907.712
'0101020304010103010111	V	D	L	K	M	V	L	I	V	W	T	-907.709

**'0201020105010105010110**	I	D	L	R	V	V	L	M	V	W	R	-907.708
**'0101020201020101030105**	V	D	L	H	L	I	L	V	I	W	K	-907.704
**'0201030101020101040110**	I	D	M	R	L	I	L	V	L	W	R	-907.703
**'0301010102040101020101**	L	D	I	R	F	M	L	V	A	W	H	-907.701
**'0101040102030401050101**	V	D	V	R	F	L	W	V	M	W	H	-907.698
**'0301010203010103010109**	L	D	I	H	I	V	L	I	V	W	Q	-907.698
**'0101020102020401010105**	V	D	L	R	F	I	W	V	V	W	K	-907.696
**'0301010102010202010112**	L	D	I	R	F	V	A	A	V	W	V	-907.696
**'0301010204040101010104**	L	D	I	H	M	M	L	V	V	W	I	-907.692
**'0201020102030101010104**	I	D	L	R	F	L	L	V	V	W	I	-907.69
**'0301010201020101020108**	L	D	I	H	L	I	L	V	A	W	N	-907.689
**'0201010105010101030110**	I	D	I	R	V	V	L	V	I	W	R	-907.688
**'0101010103030103020111**	V	D	I	R	I	L	L	I	A	W	T	-907.688
**'0201010203010101060110**	I	D	I	H	I	V	L	V	S	W	R	-907.687
**'0101010105030103070108**	V	D	I	R	V	L	L	I	T	W	N	-907.687
**'0101030103010304010109**	V	D	M	R	I	V	V	L	V	W	Q	-907.686
**'0201010103010402010101**	I	D	I	R	I	V	W	A	V	W	H	-907.685
**'0101010105020105010101**	V	D	I	R	V	I	L	M	V	W	H	-907.685
**'0101040302010105020101**	V	D	V	K	F	V	L	M	A	W	H	-907.684
**'0101020102030104010110**	V	D	L	R	F	L	L	L	V	W	R	-907.683
**'0101010102040203010106**	V	D	I	R	F	M	A	I	V	W	L	-907.682
**'0101010102020401020111**	V	D	I	R	F	I	W	V	A	W	T	-907.682
**'0101030305030104010101**	V	D	M	K	V	L	L	L	V	W	H	-907.68
**'0301040105010105010108**	L	D	V	R	V	V	L	M	V	W	N	-907.679
**'0301030103010104010104**	L	D	M	R	I	V	L	L	V	W	I	-907.679
**'0101010202010401020105**	V	D	I	H	F	V	W	V	A	W	K	-907.676
**'0101010102010303070106**	V	D	I	R	F	V	V	I	T	W	L	-907.675
**'0101040103010205010112**	V	D	V	R	I	V	A	M	V	W	V	-907.675
**'0101040105010404010105**	V	D	V	R	V	V	W	L	V	W	K	-907.673
**'0101040103030102010103**	V	D	V	R	I	L	L	A	V	W	F	-907.672
**'0201010205030101030101**	I	D	I	H	V	L	L	V	I	W	H	-907.671
**'0101010402040103010111**	V	D	I	Q	F	M	L	I	V	W	T	-907.671
**'0101030103040103070101**	V	D	M	R	I	M	L	I	T	W	H	-907.668
**'0101040105040101030109**	V	D	V	R	V	M	L	V	I	W	Q	-907.667
**'0301010305010101010112**	L	D	I	K	V	V	L	V	V	W	V	-907.666
**'0101010305030101050103**	V	D	I	K	V	L	L	V	M	W	F	-907.666
**'0101030302010101050104**	V	D	M	K	F	V	L	V	M	W	I	-907.665

**'010101020203010501 0103**	V	D	I	H	F	L	L	M	V	W	F	-907.663
**'010103010204010106 0101**	V	D	M	R	F	M	L	V	S	W	H	-907.658
**'010101040202010102 0109**	V	D	I	Q	F	I	L	V	A	W	Q	-907.654
**'030101010304010107 0110**	L	D	I	R	I	M	L	V	T	W	R	-907.653
**'010101010304010303 0106**	V	D	I	R	I	M	L	I	I	W	L	-907.65
**'010101010201020107 0105**	V	D	I	R	F	V	A	V	T	W	K	-907.649
**'030101010401010104 0106**	L	D	I	R	M	V	L	V	L	W	L	-907.648
**'010101010301010307 0112**	V	D	I	R	I	V	L	I	T	W	V	-907.648
**'010102010201020104 0110**	V	D	L	R	F	V	A	V	L	W	R	-907.648
**'010104010303010501 0112**	V	D	V	R	I	L	L	M	V	W	V	-907.647
**'010104010302010403 0101**	V	D	V	R	I	I	L	L	I	W	H	-907.645
**'010101030404010102 0109**	V	D	I	K	M	M	L	V	A	W	Q	-907.644
**'010101020201010306 0111**	V	D	I	H	F	V	L	I	S	W	T	-907.644
**'010104010201010206 0110**	V	D	V	R	F	V	L	A	S	W	R	-907.644
**'020101010303040101 0106**	I	D	I	R	I	L	W	V	V	W	L	-907.643
**'030104010501010502 0101**	L	D	V	R	V	V	L	M	A	W	H	-907.642
**'010104010504020101 0106**	V	D	V	R	V	M	A	V	V	W	L	-907.642
**'020102010502010105 0101**	I	D	L	R	V	I	L	V	M	W	H	-907.639
**'010101030401010104 0108**	V	D	I	K	M	V	L	V	L	W	N	-907.636
**'030101030401010401 0104**	L	D	I	K	M	V	L	L	V	W	I	-907.636
**'030103010201010201 0110**	L	D	M	R	F	V	L	A	V	W	R	-907.635
**'010104010101010407 0101**	V	D	V	R	L	V	L	L	T	W	H	-907.635
**'010101010103010301 0106**	V	D	I	R	L	L	L	I	V	W	L	-907.634
**'010101010502010107 0112**	V	D	I	R	V	I	L	V	T	W	V	-907.634
**'010102010503010301 0102**	V	D	L	R	V	L	L	I	V	W	E	-907.631
**'020101020102010104 0110**	I	D	I	H	L	I	L	V	L	W	R	-907.629
**'010101020303010301 0105**	V	D	I	H	I	L	L	I	V	W	K	-907.627
**'010101020201010503 0112**	V	D	I	H	F	V	L	M	I	W	V	-907.625
**'010104040103010101 0101**	V	D	V	Q	L	L	L	V	V	W	H	-907.625
**'010101030501010302 0107**	V	D	I	K	V	V	L	I	A	W	M	-907.624
**'010101010303020102 0112**	V	D	I	R	I	L	A	V	A	W	V	-907.623
**'010103010501010502 0104**	V	D	M	R	V	V	L	M	A	W	I	-907.62
**'010101040202040101 0102**	V	D	I	Q	F	I	W	V	V	W	E	-907.618
**'010101030302010307 0101**	V	D	I	K	I	I	L	I	T	W	H	-907.616
**'010101010404010102 0101**	V	D	I	R	M	M	L	V	A	W	H	-907.616
**'030101010301010504 0104**	L	D	I	R	I	V	L	M	L	W	I	-907.613
**'030104010204010101 0106**	L	D	V	R	F	M	L	V	V	W	L	-907.613

'0201010102010101060105	I	D	I	R	F	V	L	V	S	W	K	-907.611
'0101020102030201010102	V	D	L	R	F	L	A	V	V	W	E	-907.61
'0101010102020401050101	V	D	I	R	F	I	W	V	M	W	H	-907.609
'0101030102040101030111	V	D	M	R	F	M	L	V	I	W	T	-907.608
'0201010103010301040104	I	D	I	R	I	V	V	V	L	W	I	-907.608
'0201010404010103010101	I	D	I	Q	M	V	L	I	V	W	H	-907.608
'0301040102010103010105	L	D	V	R	F	V	L	I	V	W	K	-907.607
'0301010304010102010106	L	D	I	K	M	V	L	A	V	W	L	-907.605
'0101010303010103010101	V	D	I	K	I	V	L	I	V	W	H	-907.604
'0201040402010101010105	I	D	V	Q	F	V	L	V	V	W	K	-907.604
'0101040105040101020110	V	D	V	R	V	M	L	V	A	W	R	-907.603
'0101040102030104050101	V	D	V	R	F	L	L	L	M	W	H	-907.601
'0201010102010101010106	I	D	I	R	F	V	L	V	V	W	L	-907.599
'0201010103010103040109	I	D	I	R	I	V	L	I	L	W	Q	-907.599
'0101010204040301010101	V	D	I	H	M	M	V	V	V	W	H	-907.597
'0101020103020101050111	V	D	L	R	I	I	L	V	M	W	T	-907.596
'0101040404010101030110	V	D	V	Q	M	V	L	V	I	W	R	-907.596
'0101010103020401070106	V	D	I	R	I	I	W	V	T	W	L	-907.595
'0101010103040201010101	V	D	I	R	I	M	A	V	V	W	H	-907.594
'0101010202010301020106	V	D	I	H	F	V	V	V	A	W	L	-907.593
'0101020102020102010110	V	D	L	R	F	I	L	A	V	W	R	-907.59
'0101040103010304070101	V	D	V	R	I	V	V	L	T	W	H	-907.587
'0101010205020301070101	V	D	I	H	V	I	V	V	T	W	H	-907.586
'0301040201020101040101	L	D	V	H	L	I	L	V	L	W	H	-907.584
'0101010204040101020102	V	D	I	H	M	M	L	V	A	W	E	-907.579
'0101010104030103010101	V	D	I	R	M	L	L	I	V	W	H	-907.578
'0101010103030104030105	V	D	I	R	I	L	L	L	I	W	K	-907.578
'0101020102030102010108	V	D	L	R	F	L	L	A	V	W	N	-907.577
'0101020302010101010110	V	D	L	K	F	V	L	V	V	W	R	-907.577
'0101010102040103050101	V	D	I	R	F	M	L	I	M	W	H	-907.574
'0101040101010103030109	V	D	V	R	L	V	L	I	I	W	Q	-907.573
'0101030105010301070108	V	D	M	R	V	V	V	V	T	W	N	-907.573
'0101010405030103010101	V	D	I	Q	V	L	L	I	V	W	H	-907.572
'0101010203010304010106	V	D	I	H	I	V	V	L	V	W	L	-907.571
'0101010305010104070112	V	D	I	K	V	V	L	L	T	W	V	-907.57
'0101010205010201030106	V	D	I	H	V	V	A	V	I	W	L	-907.57
'0201010105010105030102	I	D	I	R	V	V	L	M	I	W	E	-907.568

**'0101040103010203050101**	V	D	V	R	I	V	A	I	M	W	H	-907.566
**'0101040103040103010101**	V	D	V	R	I	M	L	I	V	W	H	-907.566
**'0301010102010105070104**	L	D	I	R	F	V	L	M	T	W	I	-907.561
**'0101040105010102040110**	V	D	V	R	V	V	L	A	L	W	R	-907.56
**'0201010203010104010106**	I	D	I	H	I	V	L	L	V	W	L	-907.559
**'0201030103010101010110**	I	D	M	R	I	V	L	V	V	W	R	-907.558
**'0101010404010104040112**	V	D	I	Q	M	V	L	L	L	W	V	-907.557
**'0201040203010104010101**	I	D	V	H	I	V	L	L	V	W	H	-907.556
**'0101020102010101030106**	V	D	L	R	F	V	L	V	I	W	L	-907.551
**'0301040105010201010101**	L	D	V	R	V	V	A	V	V	W	H	-907.55
**'0301010102020401010104**	L	D	I	R	F	I	W	V	V	W	I	-907.549
**'0101040105010401040105**	V	D	V	R	V	V	W	V	L	W	K	-907.546
**'0101020102040101040107**	V	D	L	R	F	M	L	V	L	W	M	-907.546
**'0301010102010104050101**	L	D	I	R	F	V	L	L	M	W	H	-907.543
**'0201010205010101070109**	I	D	I	H	V	V	L	V	T	W	Q	-907.54
**'0101030404010101040110**	V	D	M	Q	M	V	L	V	L	W	R	-907.539
**'0101030201010103050101**	V	D	M	H	L	V	L	I	M	W	H	-907.539
**'0101010402010105010101**	V	D	I	Q	F	V	L	M	V	W	H	-907.534
**'0101030105020401010112**	V	D	M	R	V	I	W	V	V	W	V	-907.534
**'0301040102010201010104**	L	D	V	R	F	V	A	V	V	W	I	-907.531
**'0101040103010105010106**	V	D	V	R	I	V	L	M	V	W	L	-907.531
**'0201010105040101070111**	I	D	I	R	V	M	L	V	T	W	T	-907.53
**'0301010101020101010105**	L	D	I	R	L	I	L	V	V	W	K	-907.529
**'0101010101010403040106**	V	D	I	R	L	V	W	I	L	W	L	-907.529
**'0101030101030301070104**	V	D	M	R	L	L	V	V	T	W	I	-907.528
**'0101030404030101040101**	V	D	M	Q	M	L	L	V	L	W	H	-907.527
**'0101010102010404040105**	V	D	I	R	F	V	W	L	L	W	K	-907.527
**'0301010205010101030112**	L	D	I	H	V	V	L	V	I	W	V	-907.521
**'0201020103010101040106**	I	D	L	R	I	V	L	V	L	W	L	-907.519
**'0101010405010104040105**	V	D	I	Q	V	V	L	L	L	W	K	-907.518
**'0201040101020101040106**	I	D	V	R	L	I	L	V	L	W	L	-907.513
**'0301010103010104040109**	L	D	I	R	I	V	L	L	L	W	Q	-907.513
**'0101010104020401010104**	V	D	I	R	M	I	W	V	V	W	I	-907.513
**'0101010205010301040101**	V	D	I	H	V	V	V	V	L	W	H	-907.513
**'0101010302020102070101**	V	D	I	K	F	I	L	A	T	W	H	-907.512
**'0301030103010104070101**	L	D	M	R	I	V	L	L	T	W	H	-907.512
**'0201010103030201010110**	I	D	I	R	I	L	A	V	V	W	R	-907.511

**'0201010104010101040108**	I	D	I	R	M	V	L	V	L	W	N	-907.51
**'0101030102010203030101**	V	D	M	R	F	V	A	I	I	W	H	-907.509
**'0201030105010101020112**	I	D	M	R	V	V	L	V	A	W	V	-907.509
**'0101010201030401010105**	V	D	I	H	L	L	W	V	V	W	K	-907.507
**'0101030104010105040104**	V	D	M	R	M	V	L	M	L	W	I	-907.506
**'0101010303020101070102**	V	D	I	K	I	I	L	V	T	W	E	-907.505
**'0201010204020101010108**	I	D	I	H	M	I	L	V	V	W	N	-907.504
**'0101010104030103030107**	V	D	I	R	M	L	L	I	I	W	M	-907.504
**'0101010403010105040106**	V	D	I	Q	I	V	L	M	L	W	L	-907.503
**'0101040201010103040112**	V	D	V	H	L	V	L	I	L	W	V	-907.502
**'0101030102010102050110**	V	D	M	R	F	V	L	A	M	W	R	-907.497
**'0101030203040201010101**	V	D	M	H	I	M	A	V	V	W	H	-907.496
**'0301010201010104020105**	L	D	I	H	L	V	L	L	A	W	K	-907.495
**'0101020102010304010101**	V	D	L	R	F	V	V	L	V	W	H	-907.494
**'0201010104020101030105**	I	D	I	R	M	I	L	V	I	W	K	-907.49
**'0301010102010101030109**	L	D	I	R	F	V	L	V	I	W	Q	-907.487
**'0101030105010301010101**	V	D	M	R	V	V	V	V	V	W	H	-907.486
**'0101030102010403070101**	V	D	M	R	F	V	W	I	T	W	H	-907.482
**'0101020102010303010109**	V	D	L	R	F	V	V	I	V	W	Q	-907.481
**'0101010105010105030104**	V	D	I	R	V	V	L	M	I	W	I	-907.481
**'0101010405030301010104**	V	D	I	Q	V	L	V	V	V	W	I	-907.477
**'0101030201020104010110**	V	D	M	H	L	I	L	L	V	W	R	-907.477
**'0101040105010101020106**	V	D	V	R	V	V	L	V	A	W	L	-907.476
**'0201010201040101070106**	I	D	I	H	L	M	L	V	T	W	L	-907.475
**'0301010103010104020105**	L	D	I	R	I	V	L	L	A	W	K	-907.474
**'0101040101030201050101**	V	D	V	R	L	L	A	V	M	W	H	-907.473
**'0301040205010101020101**	L	D	V	H	V	V	L	V	A	W	H	-907.471
**'0301030105010104040101**	L	D	M	R	V	V	L	L	L	W	H	-907.467
**'0101010105030301050102**	V	D	I	R	V	L	V	V	M	W	E	-907.465
**'0101030402010403010101**	V	D	M	Q	F	V	W	I	V	W	H	-907.462
**'0101010405010105040108**	V	D	I	Q	V	V	L	M	L	W	N	-907.462
**'0101040105040101050111**	V	D	V	R	V	M	L	V	M	W	T	-907.458
**'0101040103020404010101**	V	D	V	R	I	I	W	L	V	W	H	-907.458
**'0101010402010103050103**	V	D	I	Q	F	V	L	I	M	W	F	-907.456
**'0101040104040101040108**	V	D	V	R	M	M	L	V	L	W	N	-907.456
**'0101020103010103050108**	V	D	L	R	I	V	L	I	M	W	N	-907.456
**'0101030102040301010111**	V	D	M	R	F	M	V	V	V	W	T	-907.454

**'010101010501030402010 4**	V	D	I	R	V	V	V	L	A	W	I	-907.454
**'0201030105010105020101**	I	D	M	R	V	V	L	M	A	W	H	-907.454
**'0101040105010105070108**	V	D	V	R	V	V	L	M	T	W	N	-907.453
**'0101020102010104050110**	V	D	L	R	F	V	L	L	M	W	R	-907.448
**'0101010302020104010105**	V	D	I	K	F	I	L	L	V	W	K	-907.448
**'0201010203030301010101**	I	D	I	H	I	L	V	V	V	W	H	-907.447
**'0201010402010105070101**	I	D	I	Q	F	V	L	M	T	W	H	-907.447
**'0201030102010101030109**	I	D	M	R	F	V	L	V	I	W	Q	-907.446
**'0101010105010301020108**	V	D	I	R	V	V	V	V	A	W	N	-907.445
**'0201010102010402010106**	I	D	I	R	F	V	W	A	V	W	L	-907.445
**'0101010103020303070101**	V	D	I	R	I	I	V	I	T	W	H	-907.445
**'0201010405010105010112**	I	D	I	Q	V	V	L	M	V	W	V	-907.442
**'0301010105020101050105**	L	D	I	R	V	I	L	V	M	W	K	-907.441
**'0101030303030101010103**	V	D	M	K	I	L	L	V	V	W	F	-907.439
**'0301010202010102070101**	L	D	I	H	F	V	L	A	T	W	H	-907.437
**'0301010103010102030108**	L	D	I	R	I	V	L	A	I	W	N	-907.437
**'0101010204030101010108**	V	D	I	H	M	L	L	V	V	W	N	-907.432
**'0101040104020201010101**	V	D	V	R	M	I	A	V	V	W	H	-907.431
**'0301010203010104070101**	L	D	I	H	I	V	L	L	T	W	H	-907.43
**'0101020102040101050110**	V	D	L	R	F	M	L	V	M	W	R	-907.427
**'0101020402010104010101**	V	D	L	Q	F	V	L	L	V	W	H	-907.425
**'0101010405020103010106**	V	D	I	Q	V	I	L	I	V	W	L	-907.423
**'0101030404020101010105**	V	D	M	Q	M	I	L	V	V	W	K	-907.42
**'0301040102010102060101**	L	D	V	R	F	V	L	A	S	W	H	-907.42
**'0101010204030301020101**	V	D	I	H	M	L	V	V	A	W	H	-907.419
**'0101010303020103010109**	V	D	I	K	I	I	L	I	V	W	Q	-907.418
**'0201030103010105040101**	I	D	M	R	I	V	L	M	L	W	H	-907.418
**'0101010102020401030108**	V	D	I	R	F	I	W	V	I	W	N	-907.417
**'0101010402010103060112**	V	D	I	Q	F	V	L	I	S	W	V	-907.416
**'0101010102030301020101**	V	D	I	R	F	L	V	V	A	W	H	-907.416
**'0101010205040101060108**	V	D	I	H	V	M	L	V	S	W	N	-907.415
**'0301040101020101020105**	L	D	V	R	L	I	L	V	A	W	K	-907.414
**'0201040103010105050101**	I	D	V	R	I	V	L	M	M	W	H	-907.414
**'0101030105020301050101**	V	D	M	R	V	I	V	V	M	W	H	-907.413
**'0201020201020101010101**	I	D	L	H	L	I	L	V	V	W	H	-907.413
**'0101010204030401010112**	V	D	I	H	M	L	W	V	V	W	V	-907.412
**'0101010305030103010112**	V	D	I	K	V	L	L	I	V	W	V	-907.412

'010101010301030307010 5	V	D	I	R	I	V	V	I	T	W	K	-907.409
'01010101050104010401 04	V	D	I	R	V	V	W	V	L	W	I	-907.408
'02010302010301010101 10	I	D	M	H	L	L	L	V	V	W	R	-907.407
'01010401040101050201 11	V	D	V	R	M	V	L	M	A	W	T	-907.405
'02010103020201020101 01	I	D	I	K	F	I	L	A	V	W	H	-907.404
'01010102040101040201 02	V	D	I	H	M	V	L	L	A	W	E	-907.402
'01010103040103010701 04	V	D	I	K	M	V	V	V	T	W	I	-907.402
'01010101020403010701 07	V	D	I	R	F	M	V	V	T	W	M	-907.399
'02010301030101010501 01	I	D	M	R	I	V	L	V	M	W	H	-907.399
'01010104050201010101 10	V	D	I	Q	V	I	L	V	V	W	R	-907.399
'01010101020103010701 01	V	D	I	R	F	V	V	V	T	W	H	-907.398
'01010102050101010201 04	V	D	I	H	V	V	L	V	A	W	I	-907.398
'01010102050101030201 02	V	D	I	H	V	V	L	I	A	W	E	-907.397
'01010303050401010101 11	V	D	M	K	V	M	L	V	V	W	T	-907.397
'03010101030101040701 10	L	D	I	R	I	V	L	L	T	W	R	-907.395
'02010101010204020101 05	I	D	I	R	L	I	W	A	V	W	K	-907.395
'01010401020103010701 07	V	D	V	R	F	V	V	V	T	W	M	-907.393
'01010102030302010101 08	V	D	I	H	I	L	A	V	V	W	N	-907.393
'01010401050102030101 03	V	D	V	R	V	V	A	I	V	W	F	-907.392
'03010302010201020101 01	L	D	M	H	L	I	L	A	V	W	H	-907.389
'03010101020401020101 09	L	D	I	R	F	M	L	A	V	W	Q	-907.387
'01010101020401030301 09	V	D	I	R	F	M	L	I	I	W	Q	-907.386
'01010401010201050401 06	V	D	V	R	L	I	L	M	L	W	L	-907.385
'01010101010201030101 08	V	D	I	R	L	I	L	I	V	W	N	-907.384
'03010101050203010101 01	L	D	I	R	V	I	V	V	V	W	H	-907.384
'01010101020103040701 05	V	D	I	R	F	V	V	L	T	W	K	-907.378
'01010401020104010101 04	V	D	V	R	F	V	W	V	V	W	I	-907.378
'03010102030301040101 01	L	D	I	H	I	L	L	L	V	W	H	-907.378
'01010201010101040101 06	V	D	L	R	L	V	L	L	V	W	L	-907.376
'01010102030101050401 01	V	D	I	H	I	V	L	M	L	W	H	-907.376
'01010301020101050501 06	V	D	M	R	F	V	L	M	M	W	L	-907.375
'01010401030101050401 07	V	D	V	R	I	V	L	M	L	W	M	-907.375
'02010102030101010401 04	I	D	I	H	I	V	L	V	L	W	I	-907.373
'01010301010401010501 06	V	D	M	R	L	M	L	V	M	W	L	-907.37
'03010101050401010101 10	L	D	I	R	V	M	L	V	V	W	R	-907.367
'01010101050201030201 10	V	D	I	R	V	I	L	I	A	W	R	-907.367
'01010101040301010201 12	V	D	I	R	M	L	L	V	A	W	V	-907.367

'0101010103020102070106	V	D	I	R	I	I	L	A	T	W	L	-907.367
'0201040102010101010103	I	D	V	R	F	V	L	V	V	W	F	-907.366
'0201030102010101060110	I	D	M	R	F	V	L	V	S	W	R	-907.364
'0101010103020104030104	V	D	I	R	I	I	L	L	I	W	I	-907.363
'0101020102010105040104	V	D	L	R	F	V	L	M	L	W	I	-907.362
'0301010102020201010111	L	D	I	R	F	I	A	V	V	W	T	-907.362
'0301010103010204010104	L	D	I	R	I	V	A	L	V	W	I	-907.362
'0101040302010101060105	V	D	V	K	F	V	L	V	S	W	K	-907.362
'0301010102020101060101	L	D	I	R	F	I	L	V	S	W	H	-907.36
'0101010102030102040110	V	D	I	R	F	L	L	A	L	W	R	-907.359
'0101020102010101050103	V	D	L	R	F	V	L	V	M	W	F	-907.359
'0201010302030101010102	I	D	I	K	F	L	L	V	V	W	E	-907.357
'0101040101030401010105	V	D	V	R	L	L	W	V	V	W	K	-907.357
'0101020302030201010101	V	D	L	K	F	L	A	V	V	W	H	-907.357
'0101030203010101020105	V	D	M	H	I	V	L	V	A	W	K	-907.357
'0101040201020105010105	V	D	V	H	L	I	L	M	V	W	K	-907.356
'0101020102020201070101	V	D	L	R	F	I	A	V	T	W	H	-907.353
'0101010203020103020101	V	D	I	H	I	I	L	I	A	W	H	-907.353
'0201010405010104010106	I	D	I	Q	V	V	L	L	V	W	L	-907.352
'0101010102010403070112	V	D	I	R	F	V	W	I	T	W	V	-907.352
'0101040104020103060101	V	D	V	R	M	I	L	I	S	W	H	-907.351
'0101010402010101010106	V	D	I	Q	F	V	L	V	V	W	L	-907.349
'0301040303020101010101	L	D	V	K	I	I	L	V	V	W	H	-907.347
'0301010103030101010105	L	D	I	R	I	L	L	V	V	W	K	-907.347
'0101010201010103010110	V	D	I	H	L	V	L	I	V	W	R	-907.345
'0101010302030301050101	V	D	I	K	F	L	V	V	M	W	H	-907.342
'0101040105010204070101	V	D	V	R	V	V	A	L	T	W	H	-907.342
'0101010201020301010105	V	D	I	H	L	I	V	V	V	W	K	-907.342
'0201010203010101020111	I	D	I	H	I	V	L	V	A	W	T	-907.341
'0101040102030105010110	V	D	V	R	F	L	L	M	V	W	R	-907.34
'0101030105010103060111	V	D	M	R	V	V	L	I	S	W	T	-907.339
'0301040103010101040109	L	D	V	R	I	V	L	V	L	W	Q	-907.338
'0201040105010201010110	I	D	V	R	V	V	A	V	V	W	R	-907.335
'0301030105010103010106	L	D	M	R	V	V	L	I	V	W	L	-907.334
'0101010305040301010112	V	D	I	K	V	M	V	V	V	W	V	-907.327
'0201040104010103010110	I	D	V	R	M	V	L	I	V	W	R	-907.326
'0101010403030101060106	V	D	I	Q	I	L	L	V	S	W	L	-907.323

**'0101040102040103030101**	V	D	V	R	F	M	L	I	I	W	H	-907.321
**'0101040401010103040108**	V	D	V	Q	L	V	L	I	L	W	N	-907.318
**'0101010304020105010101**	V	D	I	K	M	I	L	M	V	W	H	-907.315
**'0301030102010101040102**	L	D	M	R	F	V	L	V	L	W	E	-907.314
**'0101030203010401040101**	V	D	M	H	I	V	W	V	L	W	H	-907.313
**'0101020105010104030104**	V	D	L	R	V	V	L	L	I	W	I	-907.313
**'0101040103040101060104**	V	D	V	R	I	M	L	V	S	W	I	-907.313
**'0101040101030103070105**	V	D	V	R	L	L	L	I	T	W	K	-907.31
**'0101010404040101010109**	V	D	I	Q	M	M	L	V	V	W	Q	-907.307
**'0101010105010304010108**	V	D	I	R	V	V	V	L	V	W	N	-907.304
**'0201010302010105010102**	I	D	I	K	F	V	L	M	V	W	E	-907.302
**'0101030105040301020101**	V	D	M	R	V	M	V	V	A	W	H	-907.302
**'0101010305020105010105**	V	D	I	K	V	I	L	M	V	W	K	-907.301
**'0301040302010101070101**	L	D	V	K	F	V	L	V	T	W	H	-907.299
**'0101020302010104010102**	V	D	L	K	F	V	L	L	V	W	E	-907.299
**'0101020105020101060110**	V	D	L	R	V	I	L	V	S	W	R	-907.298
**'0101030301010103030105**	V	D	M	K	L	V	L	I	I	W	K	-907.295
**'0101020103010101050101**	V	D	L	R	I	V	L	V	M	W	H	-907.294
**'0101030105030301070101**	V	D	M	R	V	L	V	V	T	W	H	-907.293
**'0301020103020101010101**	L	D	L	R	I	I	L	V	V	W	H	-907.292
**'0201040401020103010101**	I	D	V	Q	L	I	L	I	V	W	H	-907.291
**'0101010403030301050101**	V	D	I	Q	I	L	V	V	M	W	H	-907.29
**'0101040105010101070111**	V	D	V	R	V	V	L	V	T	W	T	-907.289
**'0101030101030103050109**	V	D	M	R	L	L	L	I	M	W	Q	-907.289
**'0201010201030101040112**	I	D	I	H	L	L	L	V	L	W	V	-907.288
**'0101030104030103010102**	V	D	M	R	M	L	L	I	V	W	E	-907.288
**'0101040101020103030107**	V	D	V	R	L	I	L	I	I	W	M	-907.287
**'0301040202030101010101**	L	D	V	H	F	L	L	V	V	W	H	-907.282
**'0101040103040101050109**	V	D	V	R	I	M	L	V	M	W	Q	-907.282
**'0101010205020105010109**	V	D	I	H	V	I	L	M	V	W	Q	-907.282
**'0101010104010101020108**	V	D	I	R	M	V	L	V	A	W	N	-907.281
**'0101040102010404010102**	V	D	V	R	F	V	W	L	V	W	E	-907.279
**'0101040203010101060108**	V	D	V	H	I	V	L	V	S	W	N	-907.276
**'0101010102030401050106**	V	D	I	R	F	L	W	V	M	W	L	-907.275
**'0301040305010104010101**	L	D	V	K	V	V	L	L	V	W	H	-907.274
**'0101010305010304010104**	V	D	I	K	V	V	V	L	V	W	I	-907.274
**'0101010105020104050109**	V	D	I	R	V	I	L	L	M	W	Q	-907.273

**'0101010103020303010106**	V	D	I	R	I	I	V	I	V	W	L	-907.272
**'0101030103010203010107**	V	D	M	R	I	V	A	I	V	W	M	-907.272
**'0101010403010104070109**	V	D	I	Q	I	V	L	L	T	W	Q	-907.269
**'0201030403030101010101**	I	D	M	Q	I	L	L	V	V	W	H	-907.268
**'0101010402010203010101**	V	D	I	Q	F	V	A	I	V	W	H	-907.268
**'0101010205010105070104**	V	D	I	H	V	V	L	M	T	W	I	-907.264
**'0101040401010104010106**	V	D	V	Q	L	V	L	L	V	W	L	-907.263
**'0101040403040101010112**	V	D	V	Q	I	M	L	V	V	W	V	-907.26
**'0101030105030101060110**	V	D	M	R	V	L	L	V	S	W	R	-907.26
**'0101010305010105040110**	V	D	I	K	V	V	L	M	L	W	R	-907.258
**'0101010204010204010106**	V	D	I	H	M	V	A	L	V	W	L	-907.256
**'0201010202020104010101**	I	D	I	H	F	I	L	L	V	W	H	-907.253
**'0101020204020101010105**	V	D	L	H	M	I	L	V	V	W	K	-907.251
**'0101010205010105040102**	V	D	I	H	V	V	L	M	L	W	E	-907.248
**'0201010102030301040101**	I	D	I	R	F	L	V	V	L	W	H	-907.247
**'0101010402040101030102**	V	D	I	Q	F	M	L	V	I	W	E	-907.247
**'0101010302010105010107**	V	D	I	K	F	V	L	M	V	W	M	-907.245
**'0301010201020201010104**	L	D	I	H	L	I	A	V	V	W	I	-907.245
**'0101010402010404010105**	V	D	I	Q	F	V	W	L	V	W	K	-907.244
**'0301010104040101040104**	L	D	I	R	M	M	L	V	L	W	I	-907.243
**'0101040102040102010109**	V	D	V	R	F	M	L	A	V	W	Q	-907.242
**'0101010405010102040104**	V	D	I	Q	V	V	L	A	L	W	I	-907.241
**'0101010103030103030103**	V	D	I	R	I	L	L	I	I	W	F	-907.24
**'0101010302030403010101**	V	D	I	K	F	L	W	I	V	W	H	-907.237
**'0101010103030401020101**	V	D	I	R	I	L	W	V	A	W	H	-907.236
**'0101020104010103070111**	V	D	L	R	M	V	L	I	T	W	T	-907.236
**'0101010301040101030104**	V	D	I	K	L	M	L	V	I	W	I	-907.236
**'0301010405040101010106**	L	D	I	Q	V	M	L	V	V	W	L	-907.235
**'0201030101030101010108**	I	D	M	R	L	L	L	V	V	W	N	-907.234
**'0101040202010101050111**	V	D	V	H	F	V	L	V	M	W	T	-907.234
**'0101030204010101030110**	V	D	M	H	M	V	L	V	I	W	R	-907.233
**'0201010104040101020104**	I	D	I	R	M	M	L	V	A	W	I	-907.23
**'0101030103020301010111**	V	D	M	R	I	I	V	V	V	W	T	-907.229
**'0101010104020105010104**	V	D	I	R	M	I	L	M	V	W	I	-907.229
**'0101030405010101030105**	V	D	M	Q	V	V	L	V	I	W	K	-907.227
**'0301010105010102040108**	L	D	I	R	V	V	L	A	L	W	N	-907.227
**'0201010105010301040108**	I	D	I	R	V	V	V	V	L	W	N	-907.222

**'0101010101030304040104**	V	D	I	R	L	L	V	L	L	W	I	-907.222
**'0101020101030103020110**	V	D	L	R	L	L	L	I	A	W	R	-907.221
**'0101030105040103050101**	V	D	M	R	V	M	L	I	M	W	H	-907.22
**'0101030104010101070101**	V	D	M	R	M	V	L	V	T	W	H	-907.22
**'0101040101020101070106**	V	D	V	R	L	I	L	V	T	W	L	-907.219
**'0101030301020101030108**	V	D	M	K	L	I	L	V	I	W	N	-907.217
**'0201010205020105010101**	I	D	I	H	V	I	L	M	V	W	H	-907.217
**'0101030105010301020104**	V	D	M	R	V	V	V	V	A	W	I	-907.216
**'0201030103020102010101**	I	D	M	R	I	I	L	A	V	W	H	-907.215
**'0101010204030101030109**	V	D	I	H	M	L	L	V	I	W	Q	-907.215
**'0301010305010104010110**	L	D	I	K	V	V	L	L	V	W	R	-907.212
**'0101010104020103050110**	V	D	I	R	M	I	L	I	M	W	R	-907.211
**'0201040101030102040101**	I	D	V	R	L	L	L	A	L	W	H	-907.211
**'0101040105010203030101**	V	D	V	R	V	V	A	I	I	W	H	-907.206
**'0101010204010103070109**	V	D	I	H	M	V	L	I	T	W	Q	-907.206
**'0201020102010101040102**	I	D	L	R	F	V	L	V	L	W	E	-907.206
**'0101040305020101010108**	V	D	V	K	V	I	L	V	V	W	N	-907.205
**'0101030104020101070102**	V	D	M	R	M	I	L	V	T	W	E	-907.202
**'0201030105010102040101**	I	D	M	R	V	V	L	A	L	W	H	-907.202
**'0101040402030201010101**	V	D	V	Q	F	L	A	V	V	W	H	-907.201
**'0101030101030301040106**	V	D	M	R	L	L	V	V	L	W	L	-907.2
**'0101040104010103020105**	V	D	V	R	M	V	L	I	A	W	K	-907.2
**'0101040102010203020101**	V	D	V	R	F	V	A	I	A	W	H	-907.2
**'0201010103040101030108**	I	D	I	R	I	M	L	V	I	W	N	-907.2
**'0101010402010102040109**	V	D	I	Q	F	V	L	A	L	W	Q	-907.198
**'0101010103010101020104**	V	D	I	R	I	V	L	V	A	W	I	-907.197
**'0301030105010101070101**	L	D	M	R	V	V	L	V	T	W	H	-907.195
**'0101010101030103070101**	V	D	I	R	L	L	L	I	T	W	H	-907.194
**'0101040305010103010105**	V	D	V	K	V	V	L	I	V	W	K	-907.193
**'0101010205030101070112**	V	D	I	H	V	L	L	V	T	W	V	-907.193
**'0101010203030101060102**	V	D	I	H	I	L	L	V	S	W	E	-907.191
**'0101030102010404010106**	V	D	M	R	F	V	W	L	V	W	L	-907.189
**'0101030201040101050101**	V	D	M	H	L	M	L	V	M	W	H	-907.188
**'0301040102010104040101**	L	D	V	R	F	V	L	L	L	W	H	-907.187
**'0101010104030201070105**	V	D	I	R	M	L	A	V	T	W	K	-907.186
**'0201010103010105030105**	I	D	I	R	I	V	L	M	I	W	K	-907.184
**'0101010204010104070101**	V	D	I	H	M	V	L	L	T	W	H	-907.183

'020104040501010101010107	I	D	V	Q	V	V	L	V	V	W	M	-907.182
'0201010403020101020101	I	D	I	Q	I	I	L	V	A	W	H	-907.182
'0101010105020404010109	V	D	I	R	V	I	W	L	V	W	Q	-907.181
'0201010201010103030112	I	D	I	H	L	V	L	I	I	W	V	-907.181
'0101040403010101010101	V	D	V	Q	I	V	L	V	V	W	H	-907.179
'0101010401010104010105	V	D	I	Q	L	V	L	L	V	W	K	-907.179
'0101010103020105010105	V	D	I	R	I	I	L	M	V	W	K	-907.178
'0101010105040101070110	V	D	I	R	V	M	L	V	T	W	R	-907.177
'0101030103040101050103	V	D	M	R	I	M	L	V	M	W	F	-907.173
'0101040101030101070108	V	D	V	R	L	L	L	V	T	W	N	-907.173
'0101010305040103010108	V	D	I	K	V	M	L	I	V	W	N	-907.171
'0101020302010103020101	V	D	L	K	F	V	L	I	A	W	H	-907.169
'0101010103030401010109	V	D	I	R	I	L	W	V	V	W	Q	-907.166
'0101020405010104010108	V	D	L	Q	V	V	L	L	V	W	N	-907.166
'0101010104040101030112	V	D	I	R	M	M	L	V	I	W	V	-907.166
'0101030201030103010110	V	D	M	H	L	L	L	I	V	W	R	-907.165
'0101010102010105050108	V	D	I	R	F	V	L	M	M	W	N	-907.164
'0101030105040301010106	V	D	M	R	V	M	V	V	V	W	L	-907.163
'0101020201030101020108	V	D	L	H	L	L	L	V	A	W	N	-907.162
'0201010105010205010104	I	D	I	R	V	V	A	M	V	W	I	-907.162
'0101040102040101030105	V	D	V	R	F	M	L	V	I	W	K	-907.161
'0101040104020105040101	V	D	V	R	M	I	L	M	L	W	H	-907.161
'0201010203030101010105	I	D	I	H	I	L	L	V	V	W	K	-907.161
'0101030102020202010101	V	D	M	R	F	I	A	A	V	W	H	-907.161
'0101010402030401010108	V	D	I	Q	F	L	W	V	V	W	N	-907.159
'0101030305010104020101	V	D	M	K	V	V	L	L	A	W	H	-907.159
'0101010302020401010112	V	D	I	K	F	I	W	V	V	W	V	-907.159
'0101010205030103010107	V	D	I	H	V	L	L	I	V	W	M	-907.156
'0101030105010105030110	V	D	M	R	V	V	L	M	I	W	R	-907.154
'0101010403010403010110	V	D	I	Q	I	V	W	I	V	W	R	-907.152
'0101020105030101060106	V	D	L	R	V	L	L	V	S	W	L	-907.151
'0101010104020101020105	V	D	I	R	M	I	L	V	A	W	K	-907.151
'0101030202010104070101	V	D	M	H	F	V	L	L	T	W	H	-907.15
'0301030101020101040108	L	D	M	R	L	I	L	V	L	W	N	-907.15
'0201010102020103050101	I	D	I	R	F	I	L	I	M	W	H	-907.15
'0101030105040103010112	V	D	M	R	V	M	L	I	V	W	V	-907.149
'0101010102010303030108	V	D	I	R	F	V	V	I	I	W	N	-907.149

**'020102010201010101010109**	I	D	L	R	F	V	L	V	V	W	Q	-907.149
**'020104010401010401010106**	I	D	V	R	M	V	L	L	V	W	L	-907.147
**'020102010303010101010105**	I	D	L	R	I	L	L	V	V	W	K	-907.147
**'020104010301010404010101**	I	D	V	R	I	V	L	L	L	W	H	-907.145
**'010101010201020106010106**	V	D	I	R	F	V	A	V	S	W	L	-907.142
**'010101010204030106010112**	V	D	I	R	F	M	V	V	S	W	V	-907.141
**'020101020301010507010101**	I	D	I	H	I	V	L	M	T	W	H	-907.141
**'010101010303030106010110**	V	D	I	R	I	L	V	V	S	W	R	-907.138
**'010102040201010104010109**	V	D	L	Q	F	V	L	V	L	W	Q	-907.136
**'020101020104010104010105**	I	D	I	H	L	M	L	V	L	W	K	-907.136
**'030101020501010106010110**	L	D	I	H	V	V	L	V	S	W	R	-907.135
**'020104010501010105010111**	I	D	V	R	V	V	L	V	M	W	T	-907.134
**'010101010201030105010104**	V	D	I	R	F	V	V	V	M	W	I	-907.134
**'020101010501040301010104**	I	D	I	R	V	V	W	I	V	W	I	-907.134
**'010101010403040102010106**	V	D	I	R	M	L	W	V	A	W	L	-907.131
**'010104010401010105010101**	V	D	V	R	M	V	L	V	M	W	H	-907.128
**'010104010301010406010107**	V	D	V	R	I	V	L	L	S	W	M	-907.127
**'010101020404010106010112**	V	D	I	H	M	M	L	V	S	W	V	-907.125
**'030101010501010403010109**	L	D	I	R	V	V	L	L	I	W	Q	-907.125
**'010103020104010103010110**	V	D	M	H	L	M	L	V	I	W	R	-907.123
**'010102010201020101010101**	V	D	L	R	F	V	A	V	V	W	H	-907.122
**'010103020403010102010101**	V	D	M	H	M	L	L	V	A	W	H	-907.122
**'030101010504010103010112**	L	D	I	R	V	M	L	V	I	W	V	-907.12
**'010101020504010201010108**	V	D	I	H	V	M	L	A	V	W	N	-907.12
**'030104010401010101010106**	L	D	V	R	M	V	L	V	V	W	L	-907.119
**'010101020303030107010101**	V	D	I	H	I	L	V	V	T	W	H	-907.119
**'030101040201010407010101**	L	D	I	Q	F	V	L	L	T	W	H	-907.116
**'010101030403010404010101**	V	D	I	K	M	L	L	L	L	W	H	-907.115
**'010101010204010302010110**	V	D	I	R	F	M	L	I	A	W	R	-907.113
**'020101020501010504010101**	I	D	I	H	V	V	L	M	L	W	H	-907.113
**'010102020102020101010105**	V	D	L	H	L	I	A	V	V	W	K	-907.113
**'020101010504010201010112**	I	D	I	R	V	M	L	A	V	W	V	-907.112
**'010102020201010106010112**	V	D	L	H	F	V	L	V	S	W	V	-907.111
**'010102010302040101010106**	V	D	L	R	I	I	W	V	V	W	L	-907.11
**'030101010204010207010101**	L	D	I	R	F	M	L	A	T	W	H	-907.107
**'010103030501020101010112**	V	D	M	K	V	V	A	V	V	W	V	-907.107
**'010104030504010101010101**	V	D	V	K	V	M	L	V	V	W	H	-907.105

'0101010205010404010104	V	D	I	H	V	V	W	L	V	W	I	-907.103
'0101010405020104050101	V	D	I	Q	V	I	L	L	M	W	H	-907.101
'0101020102010105010106	V	D	L	R	F	V	L	M	V	W	L	-907.1
'0101030103040101020110	V	D	M	R	I	M	L	V	A	W	R	-907.1
'0101040103030401040101	V	D	V	R	I	L	W	V	L	W	H	-907.099
'0101020202010104050101	V	D	L	H	F	V	L	L	M	W	H	-907.097
'0101010105020105070104	V	D	I	R	V	I	L	M	T	W	I	-907.097
'0101040403030301010101	V	D	V	Q	I	L	V	V	V	W	H	-907.097
'0101030104030104010109	V	D	M	R	M	L	L	L	V	W	Q	-907.096
'0101010302010201070109	V	D	I	K	F	V	A	V	T	W	Q	-907.095
'0101030201010101050105	V	D	M	H	L	V	L	V	M	W	K	-907.094
'0101010105010304040101	V	D	I	R	V	V	V	L	L	W	H	-907.093
'0101010105010404020106	V	D	I	R	V	V	W	L	A	W	L	-907.093
'0101030105010301050110	V	D	M	R	V	V	V	V	M	W	R	-907.093
'0101010201030301050110	V	D	I	H	L	L	V	V	M	W	R	-907.091
'0101010202020101060102	V	D	I	H	F	I	L	V	S	W	E	-907.089
'0201010102020301060101	I	D	I	R	F	I	V	V	S	W	H	-907.087
'0301020102010102010105	L	D	L	R	F	V	L	A	V	W	K	-907.087
'0101030102010301070102	V	D	M	R	F	V	V	V	T	W	E	-907.085
'0201010104040103010112	I	D	I	R	M	M	L	I	V	W	V	-907.084
'0101030105030301010112	V	D	M	R	V	L	V	V	V	W	V	-907.084
'0201010102010203010104	I	D	I	R	F	V	A	I	V	W	I	-907.083
'0101030105020304010101	V	D	M	R	V	I	V	L	V	W	H	-907.082
'0101010303020301070101	V	D	I	K	I	I	V	V	T	W	H	-907.082
'0101010102030204010105	V	D	I	R	F	L	A	L	V	W	K	-907.082
'0201040104030101010112	I	D	V	R	M	L	L	V	V	W	V	-907.08
'0101020105040101050112	V	D	L	R	V	M	L	V	M	W	V	-907.08
'0101030201040101070102	V	D	M	H	L	M	L	V	T	W	E	-907.078
'0101010101030101040110	V	D	I	R	L	L	L	V	L	W	R	-907.077
'0201010105040201010108	I	D	I	R	V	M	A	V	V	W	N	-907.077
'0201010403010103010104	I	D	I	Q	I	V	L	I	V	W	I	-907.076
'0101040103030201010103	V	D	V	R	I	L	A	V	V	W	F	-907.076
'0101010302010404010101	V	D	I	K	F	V	W	L	V	W	H	-907.071
'0101040105030301010108	V	D	V	R	V	L	V	V	V	W	N	-907.07
'0101010405040101010101	V	D	I	Q	V	M	L	V	V	W	H	-907.07
'0101010101010103030112	V	D	I	R	L	V	L	I	I	W	V	-907.069
'0101010404010103010108	V	D	I	Q	M	V	L	I	V	W	N	-907.069

**'0101010102040203070101**	V	D	I	R	F	M	A	I	T	W	H	-907.069
**'0201030105010105010109**	I	D	M	R	V	V	L	M	V	W	Q	-907.066
**'0201010102020105010105**	I	D	I	R	F	I	L	M	V	W	K	-907.063
**'0101010204010404010101**	V	D	I	H	M	V	W	L	V	W	H	-907.06
**'0101010101020101030104**	V	D	I	R	L	I	L	V	I	W	I	-907.06
**'0101020201010101040105**	V	D	L	H	L	V	L	V	L	W	K	-907.059
**'0201030102030301010101**	I	D	M	R	F	L	V	V	V	W	H	-907.058
**'0201040102010405010101**	I	D	V	R	F	V	W	M	V	W	H	-907.055
**'0201010405010103010110**	I	D	I	Q	V	V	L	I	V	W	R	-907.054
**'0201010302030301010101**	I	D	I	K	F	L	V	V	V	W	H	-907.054
**'0201010104030103070101**	I	D	I	R	M	L	L	I	T	W	H	-907.054
**'0201010102010303070101**	I	D	I	R	F	V	V	I	T	W	H	-907.054
**'0101020102010102010104**	V	D	L	R	F	V	L	A	V	W	I	-907.053
**'0101040301020101040109**	V	D	V	K	L	I	L	V	L	W	Q	-907.051
**'0101010101030101020108**	V	D	I	R	L	L	L	V	A	W	N	-907.05
**'0101040405010401010104**	V	D	V	Q	V	V	W	V	V	W	I	-907.05
**'0301030105020101060101**	L	D	M	R	V	I	L	V	S	W	H	-907.049
**'0101040103040101030107**	V	D	V	R	I	M	L	V	I	W	M	-907.049
**'0101040205010101050101**	V	D	V	H	V	V	L	V	M	W	H	-907.049
**'0101010102010203070104**	V	D	I	R	F	V	A	I	T	W	I	-907.047
**'0301010102010104010105**	L	D	I	R	F	V	L	L	V	W	K	-907.047
**'0101040205010102010111**	V	D	V	H	V	V	L	A	V	W	T	-907.046
**'0301040103020401010101**	L	D	V	R	I	I	W	V	V	W	H	-907.044
**'0101010302040103010101**	V	D	I	K	F	M	L	I	V	W	H	-907.043
**'0101010105040103010101**	V	D	I	R	V	M	L	I	V	W	H	-907.039
**'0101010105030401070110**	V	D	I	R	V	L	W	V	T	W	R	-907.035
**'0101010102010204020105**	V	D	I	R	F	V	A	L	A	W	K	-907.034
**'0101010103030401070111**	V	D	I	R	I	L	W	V	T	W	T	-907.034
**'0101010104030201040112**	V	D	I	R	M	L	A	V	L	W	V	-907.033
**'0101020202010105010102**	V	D	L	H	F	V	L	M	V	W	E	-907.033
**'0101040105010301040102**	V	D	V	R	V	V	V	V	L	W	E	-907.031
**'0101010101040101030106**	V	D	I	R	L	M	L	V	I	W	L	-907.028
**'0101010205010401010106**	V	D	I	H	V	V	W	V	V	W	L	-907.027
**'0101020101010103010102**	V	D	L	R	L	V	L	I	V	W	E	-907.027
**'0101010203040101010102**	V	D	I	H	I	M	L	V	V	W	E	-907.026
**'0101030105010102040108**	V	D	M	R	V	V	L	A	L	W	N	-907.025
**'0101010103040301060106**	V	D	I	R	I	M	V	V	S	W	L	-907.023

'0101040103030101060106	V	D	V	R	I	L	L	V	S	W	L	-907.022
'0101040203010102010104	V	D	V	H	I	V	L	A	V	W	I	-907.021
'0101010203010403020101	V	D	I	H	I	V	W	I	A	W	H	-907.02
'0101020304010101070105	V	D	L	K	M	V	L	V	T	W	K	-907.02
'0101030304040101010106	V	D	M	K	M	M	L	V	V	W	L	-907.018
'0201010102040201020101	I	D	I	R	F	M	A	V	A	W	H	-907.015
'0101010203010102020101	V	D	I	H	I	V	L	A	A	W	H	-907.012
'0101020101020101030101	V	D	L	R	L	I	L	V	I	W	H	-907.01
'0101010102020105010112	V	D	I	R	F	I	L	M	V	W	V	-907.008
'0101030102010304020101	V	D	M	R	F	V	V	L	A	W	H	-907.007
'0101010103040201070108	V	D	I	R	I	M	A	V	T	W	N	-907.006
'0301010302020103010101	L	D	I	K	F	I	L	I	V	W	H	-907.005
'0101030302030101030101	V	D	M	K	F	L	L	V	I	W	H	-907.005
'0101040401020101020104	V	D	V	Q	L	I	L	V	A	W	I	-907.003
'0101010202010105050106	V	D	I	H	F	V	L	M	M	W	L	-907.002
'0201010105030104010108	I	D	I	R	V	L	L	L	V	W	N	-907.002
'0101010201040101030109	V	D	I	H	L	M	L	V	I	W	Q	-907.001
'0101010201010104070105	V	D	I	H	L	V	L	L	T	W	K	-907.001
'0101030203010401010106	V	D	M	H	I	V	W	V	V	W	L	-907.001
'0101030202010102010110	V	D	M	H	F	V	L	A	V	W	R	-907.0
'0101010105010105050112	V	D	I	R	V	V	L	M	M	W	V	-906.998
'0201010405010101020112	I	D	I	Q	V	V	L	V	A	W	V	-906.997
'0201010204010105010109	I	D	I	H	M	V	L	M	V	W	Q	-906.996
'0101010302020104040101	V	D	I	K	F	I	L	L	L	W	H	-906.995
'0101010105030102040104	V	D	I	R	V	L	L	A	L	W	I	-906.994
'0101010204030104010101	V	D	I	H	M	L	L	L	V	W	H	-906.994
'0101010402010104030108	V	D	I	Q	F	V	L	L	I	W	N	-906.994
'0201030103010101070107	I	D	M	R	I	V	L	V	T	W	M	-906.989
'0201030105010103060101	I	D	M	R	V	V	L	I	S	W	H	-906.988
'0201030101030103010110	I	D	M	R	L	L	L	I	V	W	R	-906.988
'0101010102040201010111	V	D	I	R	F	M	A	V	V	W	T	-906.987
'0201010105010102040103	I	D	I	R	V	V	L	A	L	W	F	-906.987
'0101010204030103010104	V	D	I	H	M	L	L	I	V	W	I	-906.987
'0301010202010101040108	L	D	I	H	F	V	L	V	L	W	N	-906.986
'0101040403010101030109	V	D	V	Q	I	V	L	V	I	W	Q	-906.986
'0201010105010105060105	I	D	I	R	V	V	L	M	S	W	K	-906.985
'0201030405010102010101	I	D	M	Q	V	V	L	A	V	W	H	-906.985

**'0101010103010104020109**	V	D	I	R	I	V	L	L	A	W	Q	-906.984
**'0201030104040101010105**	I	D	M	R	M	M	L	V	V	W	K	-906.981
**'0101030302010101060112**	V	D	M	K	F	V	L	V	S	W	V	-906.981
**'0301010105010101030103**	L	D	I	R	V	V	L	V	I	W	F	-906.981
**'0301040405010101070101**	L	D	V	Q	V	V	L	V	T	W	H	-906.979
**'0101010103010404010106**	V	D	I	R	I	V	W	L	V	W	L	-906.975
**'0101010105010301030104**	V	D	I	R	V	V	V	V	I	W	I	-906.975
**'0201010101030102030110**	I	D	I	R	L	L	L	A	I	W	R	-906.975
**'0101010302010104030110**	V	D	I	K	F	V	L	L	I	W	R	-906.974
**'0101040201030101010111**	V	D	V	H	L	L	L	V	V	W	T	-906.969
**'0201010104040101040110**	I	D	I	R	M	M	L	V	L	W	R	-906.968
**'0101010305010104030106**	V	D	I	K	V	V	L	L	I	W	L	-906.964
**'0101010401020103030104**	V	D	I	Q	L	I	L	I	I	W	I	-906.963
**'0201040105010301010106**	I	D	V	R	V	V	V	V	V	W	L	-906.963
**'0301010102010304010101**	L	D	I	R	F	V	V	L	V	W	H	-906.961
**'0201010104010103010104**	I	D	I	R	M	V	L	I	V	W	I	-906.961
**'0201010404040101010108**	I	D	I	Q	M	M	L	V	V	W	N	-906.954
**'0301010403010102010109**	L	D	I	Q	I	V	L	A	V	W	Q	-906.954
**'0301010105010103010105**	L	D	I	R	V	V	L	I	V	W	K	-906.95
**'0201040402010101040101**	I	D	V	Q	F	V	L	V	L	W	H	-906.95
**'0201010105020301050101**	I	D	I	R	V	I	V	V	M	W	H	-906.948
**'0101010101020401040106**	V	D	I	R	L	I	W	V	L	W	L	-906.947
**'0101020203040101030101**	V	D	L	H	I	M	L	V	I	W	H	-906.947
**'0301010201020101010103**	L	D	I	H	L	I	L	V	V	W	F	-906.945
**'0101010404010403010105**	V	D	I	Q	M	V	W	I	V	W	K	-906.944
**'0101010202040301050101**	V	D	I	H	F	M	V	V	M	W	H	-906.942
**'0201020105010102010112**	I	D	L	R	V	V	L	A	V	W	V	-906.942
**'0101020402030101050101**	V	D	L	Q	F	L	L	V	M	W	H	-906.94
**'0201010102010102010105**	I	D	I	R	F	V	L	A	V	W	K	-906.94
**'0101010304010105070101**	V	D	I	K	M	V	L	M	T	W	H	-906.938
**'0101040101020101020108**	V	D	V	R	L	I	L	V	A	W	N	-906.937
**'0101040104010101010105**	V	D	V	R	M	V	L	V	V	W	K	-906.935
**'0101010302030401070101**	V	D	I	K	F	L	W	V	T	W	H	-906.934
**'0101040101020104040112**	V	D	V	R	L	I	L	L	L	W	V	-906.934
**'0101010402010303010105**	V	D	I	Q	F	V	V	I	V	W	K	-906.934
**'0101010304010105040112**	V	D	I	K	M	V	L	M	L	W	V	-906.931
**'0201010102010205010101**	I	D	I	R	F	V	A	M	V	W	H	-906.931

'01010403040101030101 01	V	D	V	K	M	V	L	I	V	W	H	-906.93
'01010103040104040101 04	V	D	I	K	M	V	W	L	V	W	I	-906.928
'01010403030101040101 04	V	D	V	K	I	V	L	L	V	W	I	-906.926
'01010301030104030201 01	V	D	M	R	I	V	W	I	A	W	H	-906.925
'01010403020101010101 11	V	D	V	K	F	V	L	V	V	W	T	-906.923
'01010101020401030601 02	V	D	I	R	F	M	L	I	S	W	E	-906.923
'01010104050204030101 01	V	D	I	Q	V	I	W	I	V	W	H	-906.922
'01010101030302010701 06	V	D	I	R	I	L	A	V	T	W	L	-906.921
'01010101010304030101 04	V	D	I	R	L	L	W	I	V	W	I	-906.918
'01010401040201020101 04	V	D	V	R	M	I	L	A	V	W	I	-906.917
'01010101030304010501 12	V	D	I	R	I	L	W	V	M	W	V	-906.916
'02010101040102030101 05	I	D	I	R	M	V	A	I	V	W	K	-906.916
'03010401030301010101 12	L	D	V	R	I	L	L	V	V	W	V	-906.916
'01010103040301010101 10	V	D	I	K	M	L	L	V	V	W	R	-906.915
'01010102040103010401 04	V	D	I	H	M	V	V	V	L	W	I	-906.913
'01010401010202030101 06	V	D	V	R	L	I	A	I	V	W	L	-906.912
'01010103040101010701 06	V	D	I	K	M	V	L	V	T	W	L	-906.909
'02010303020401010101 01	I	D	M	K	F	M	L	V	V	W	H	-906.908
'01010301030302010701 01	V	D	M	R	I	L	A	V	T	W	H	-906.908
'01010104050101040701 06	V	D	I	Q	V	V	L	L	T	W	L	-906.907
'01010303020102010701 01	V	D	M	K	F	V	A	V	T	W	H	-906.907
'01010102020201030101 02	V	D	I	H	F	I	L	I	V	W	E	-906.907
'02010101030102030101 08	I	D	I	R	I	V	A	I	V	W	N	-906.906
'01010102030101020301 04	V	D	I	H	I	V	L	A	I	W	I	-906.906
'01010102020401010201 10	V	D	I	H	F	M	L	V	A	W	R	-906.904
'02010102030201010101 07	I	D	I	H	I	I	L	V	V	W	M	-906.902
'01010101020203040601 01	V	D	I	R	F	I	V	L	S	W	H	-906.902
'01010302040101040401 01	V	D	M	H	M	V	L	L	L	W	H	-906.902
'03010101050201020101 07	L	D	I	R	V	I	L	A	V	W	M	-906.9
'03010301050101050101 01	L	D	M	R	V	V	L	M	V	W	H	-906.9
'02010404050101010501 01	I	D	V	Q	V	V	L	V	M	W	H	-906.9
'03010101050101050401 01	L	D	I	R	V	V	L	M	L	W	H	-906.898
'01010101020104010201 02	V	D	I	R	F	V	W	V	A	W	E	-906.897
'01010203040101010201 08	V	D	L	K	M	V	L	V	A	W	N	-906.895
'02010101050104050101 05	I	D	I	R	V	V	W	M	V	W	K	-906.893
'01010101050103040501 06	V	D	I	R	V	V	V	L	M	W	L	-906.892
'01010301050401010301 08	V	D	M	R	V	M	L	V	I	W	N	-906.89

'0301040101020104010104	L	D	V	R	L	I	L	L	V	W	I	-906.888
'0201030302010101010108	I	D	M	K	F	V	L	V	V	W	N	-906.887
'0301040103020103010101	L	D	V	R	I	I	L	I	V	W	H	-906.886
'0101030103010101020112	V	D	M	R	I	V	L	V	A	W	V	-906.881
'0301010205010105020101	L	D	I	H	V	V	L	M	A	W	H	-906.881
'0101030203010301050101	V	D	M	H	I	V	V	V	M	W	H	-906.881
'0101040102010401020108	V	D	V	R	F	V	W	V	A	W	N	-906.88
'0101020304010104010101	V	D	L	K	M	V	L	L	V	W	H	-906.879
'0101040101010104040107	V	D	V	R	L	V	L	L	L	W	M	-906.879
'0101040105010201050105	V	D	V	R	V	V	A	V	M	W	K	-906.875
'0301010203040101010112	L	D	I	H	I	M	L	V	V	W	V	-906.875
'0301010102040101060108	L	D	I	R	F	M	L	V	S	W	N	-906.875
'0101010103030101050109	V	D	I	R	I	L	L	V	M	W	Q	-906.873
'0101010102020104050107	V	D	I	R	F	I	L	L	M	W	M	-906.869
'0101010104040201010103	V	D	I	R	M	M	A	V	V	W	F	-906.867
'0101040205010101060110	V	D	V	H	V	V	L	V	S	W	R	-906.867
'0201040201030103010101	I	D	V	H	L	L	L	I	V	W	H	-906.866
'0101030301020101050105	V	D	M	K	L	I	L	V	M	W	K	-906.866
'0101010203040101050107	V	D	I	H	I	M	L	V	M	W	M	-906.865
'0301010305020101050101	L	D	I	K	V	I	L	V	M	W	H	-906.865
'0101010203010301010105	V	D	I	H	I	V	V	V	V	W	K	-906.865
'0301030105020201010101	L	D	M	R	V	I	A	V	V	W	H	-906.864
'0101020202010201010112	V	D	L	H	F	V	A	V	V	W	V	-906.864
'0101040103010301050104	V	D	V	R	I	V	V	V	M	W	I	-906.863
'0301010105040101060102	L	D	I	R	V	M	L	V	S	W	E	-906.86
'0101010102030103060110	V	D	I	R	F	L	L	I	S	W	R	-906.858
'0101030101040101040107	V	D	M	R	L	M	L	V	L	W	M	-906.857
'0301040102010101030112	L	D	V	R	F	V	L	V	I	W	V	-906.857
'0101010105020401030104	V	D	I	R	V	I	W	V	I	W	I	-906.856
'0101030102010105030109	V	D	M	R	F	V	L	M	I	W	Q	-906.856
'0101010403040101070109	V	D	I	Q	I	M	L	V	T	W	Q	-906.855
'0201010103040102010108	I	D	I	R	I	M	L	A	V	W	N	-906.855
'0101010202010301010111	V	D	I	H	F	V	V	V	V	W	T	-906.854
'0101010103030203010112	V	D	I	R	I	L	A	I	V	W	V	-906.847
'0101030102010204040101	V	D	M	R	F	V	A	L	L	W	H	-906.846
'0101030401020101010109	V	D	M	Q	L	I	L	V	V	W	Q	-906.845
'0101010102040102010101	V	D	I	R	F	M	L	A	V	W	H	-906.845

**'0101010102010405010101**	V	D	I	R	F	V	W	M	V	W	H	-906.845
**'0101030105040201010103**	V	D	M	R	V	M	A	V	V	W	F	-906.844
**'0301010102010101070112**	L	D	I	R	F	V	L	V	T	W	V	-906.842
**'0301010105010105070106**	L	D	I	R	V	V	L	M	T	W	L	-906.842
**'0101030205030301010101**	V	D	M	H	V	L	V	V	V	W	H	-906.841
**'0101030103020102030101**	V	D	M	R	I	I	L	A	I	W	H	-906.84
**'0301010102020201070101**	L	D	I	R	F	I	A	V	T	W	H	-906.839
**'0101020105010201020106**	V	D	L	R	V	V	A	V	A	W	L	-906.838
**'0101040102010402010106**	V	D	V	R	F	V	W	A	V	W	L	-906.837
**'0101020102030301010106**	V	D	L	R	F	L	V	V	V	W	L	-906.837
**'0301010105010201020105**	L	D	I	R	V	V	A	V	A	W	K	-906.837
**'0101030403010101070105**	V	D	M	Q	I	V	L	V	T	W	K	-906.836
**'0101020102010102040105**	V	D	L	R	F	V	L	A	L	W	K	-906.836
**'0101010203010101060106**	V	D	I	H	I	V	L	V	S	W	L	-906.836
**'0101010201020403010105**	V	D	I	H	L	I	W	I	V	W	K	-906.835
**'0201040102010101030101**	I	D	V	R	F	V	L	V	I	W	H	-906.835
**'0201020102010103070101**	I	D	L	R	F	V	L	I	T	W	H	-906.833
**'0101010103020103060104**	V	D	I	R	I	I	L	I	S	W	I	-906.832
**'0101010302010204010105**	V	D	I	K	F	V	A	L	V	W	K	-906.831
**'0301030202010103010101**	L	D	M	H	F	V	L	I	V	W	H	-906.831
**'0101030402030103010101**	V	D	M	Q	F	L	L	I	V	W	H	-906.83
**'0301010204010101040104**	L	D	I	H	M	V	L	V	L	W	I	-906.83
**'0101010303020101060104**	V	D	I	K	I	I	L	V	S	W	I	-906.829
**'0101030403020101010110**	V	D	M	Q	I	I	L	V	V	W	R	-906.829
**'0101010103020304010101**	V	D	I	R	I	I	V	L	V	W	H	-906.828
**'0101040105010401020102**	V	D	V	R	V	V	W	V	A	W	E	-906.828
**'0101010405030401010110**	V	D	I	Q	V	L	W	V	V	W	R	-906.827
**'0101040104020101010112**	V	D	V	R	M	I	L	V	V	W	V	-906.827
**'0201030305010101010109**	I	D	M	K	V	V	L	V	V	W	Q	-906.826
**'0101010103030103040104**	V	D	I	R	I	L	L	I	L	W	I	-906.826
**'0101020404010104010104**	V	D	L	Q	M	V	L	L	V	W	I	-906.825
**'0301040105010101020110**	L	D	V	R	V	V	L	V	A	W	R	-906.825
**'0101010103010104070106**	V	D	I	R	I	V	L	L	T	W	L	-906.824
**'0101040101020104070109**	V	D	V	R	L	I	L	L	T	W	Q	-906.822
**'0101010302040201010108**	V	D	I	K	F	M	A	V	V	W	N	-906.821
**'0101020103010104020106**	V	D	L	R	I	V	L	L	A	W	L	-906.821
**'0101010103030402010112**	V	D	I	R	I	L	W	A	V	W	V	-906.819

'0101040102030104010111	V	D	V	R	F	L	L	L	V	W	T	-906.817
'0101010404040101040104	V	D	I	Q	M	M	L	V	L	W	I	-906.817
'0101040101030301050106	V	D	V	R	L	L	V	V	M	W	L	-906.815
'0101010205030101050102	V	D	I	H	V	L	L	V	M	W	E	-906.815
'0101020102010204010102	V	D	L	R	F	V	A	L	V	W	E	-906.813
'0101010105010304030109	V	D	I	R	V	V	V	L	I	W	Q	-906.812
'0101010403030103050101	V	D	I	Q	I	L	L	I	M	W	H	-906.81
'0201010205010101060108	I	D	I	H	V	V	L	V	S	W	N	-906.809
'0101010302010101010109	V	D	I	K	F	V	L	V	V	W	Q	-906.808
'0101010105040104040106	V	D	I	R	V	M	L	L	L	W	L	-906.804
'0101010204030105010105	V	D	I	H	M	L	L	M	V	W	K	-906.803
'0201010302040201010101	I	D	I	K	F	M	A	V	V	W	H	-906.803
'0101010105020304010103	V	D	I	R	V	I	V	L	V	W	F	-906.8
'0101020103010101070108	V	D	L	R	I	V	L	V	T	W	N	-906.8
'0301030105010201010106	L	D	M	R	V	V	A	V	V	W	L	-906.8
'0201040405010104010101	I	D	V	Q	V	V	L	L	V	W	H	-906.799
'0101010103030201050102	V	D	I	R	I	L	A	V	M	W	E	-906.799
'0101020205010101050105	V	D	L	H	V	V	L	V	M	W	K	-906.799
'0101040104010101030109	V	D	V	R	M	V	L	V	I	W	Q	-906.798
'0101010404010103020101	V	D	I	Q	M	V	L	I	A	W	H	-906.793
'0101040102020201020101	V	D	V	R	F	I	A	V	A	W	H	-906.789
'0101020101020101050108	V	D	L	R	L	I	L	V	M	W	N	-906.788
'0101040201010304010104	V	D	V	H	L	V	V	L	V	W	I	-906.787
'0101010102010102030104	V	D	I	R	F	V	L	A	I	W	I	-906.787
'0101040105040201020101	V	D	V	R	V	M	A	V	A	W	H	-906.785
'0101010304010101010102	V	D	I	K	M	V	L	V	V	W	E	-906.783
'0101030102010301010109	V	D	M	R	F	V	V	V	V	W	Q	-906.783
'0201030101010103010106	I	D	M	R	L	V	L	I	V	W	L	-906.783
'0201010305010101040107	I	D	I	K	V	V	L	V	L	W	M	-906.782
'0101040201010104020112	V	D	V	H	L	V	L	L	A	W	V	-906.782
'0101010202030105070101	V	D	I	H	F	L	L	M	T	W	H	-906.781
'0201010103030201030101	I	D	I	R	I	L	A	V	I	W	H	-906.781
'0101030201030301050101	V	D	M	H	L	L	V	V	M	W	H	-906.78
'0101010203010201040106	V	D	I	H	I	V	A	V	L	W	L	-906.78
'0101010302010102040101	V	D	I	K	F	V	L	A	L	W	H	-906.78
'0101010203010103060107	V	D	I	H	I	V	L	I	S	W	M	-906.78
'0101010201020401010101	V	D	I	H	L	I	W	V	V	W	H	-906.778

'0101030102010201030112	V	D	M	R	F	V	A	V	I	W	V	-906.778
'0101040202010201010102	V	D	V	H	F	V	A	V	V	W	E	-906.776
'0201010202010105060101	I	D	I	H	F	V	L	M	S	W	H	-906.775
'0101010303010104040101	V	D	I	K	I	V	L	L	L	W	H	-906.773
'0101030304010101070104	V	D	M	K	M	V	L	V	T	W	I	-906.773
'0201040203010101010103	I	D	V	H	I	V	L	V	V	W	F	-906.77
'0301040202010101040101	L	D	V	H	F	V	L	V	L	W	H	-906.77
'0101010105010303040108	V	D	I	R	V	V	V	I	L	W	N	-906.769
'0201010103040101050104	I	D	I	R	I	M	L	V	M	W	I	-906.767
'0301010202010101030106	L	D	I	H	F	V	L	V	I	W	L	-906.766
'0101030103030104010111	V	D	M	R	I	L	L	L	V	W	T	-906.766
'0101010201020103030110	V	D	I	H	L	I	L	I	I	W	R	-906.766
'0201020101030101040110	I	D	L	R	L	L	L	V	L	W	R	-906.765
'0101040203010401070101	V	D	V	H	I	V	W	V	T	W	H	-906.765
'0201010102040401010105	I	D	I	R	F	M	W	V	V	W	K	-906.765
'0101010102040102050105	V	D	I	R	F	M	L	A	M	W	K	-906.764
'0101030201030301010105	V	D	M	H	L	L	V	V	V	W	K	-906.763
'0201010105030101030112	I	D	I	R	V	L	L	V	I	W	V	-906.761
'0101020405020101010108	V	D	L	Q	V	I	L	V	V	W	N	-906.761
'0101010102030105010101	V	D	I	R	F	L	L	M	V	W	H	-906.76
'0201030101020101050105	I	D	M	R	L	I	L	V	M	W	K	-906.76
'0101030105010301040109	V	D	M	R	V	V	V	V	L	W	Q	-906.759
'0101030305010101020103	V	D	M	K	V	V	L	V	A	W	F	-906.759
'0201040104010303010101	I	D	V	R	M	V	V	I	V	W	H	-906.759
'0101010203010201050104	V	D	I	H	I	V	A	V	M	W	I	-906.759
'0101040303010103010108	V	D	V	K	I	V	L	I	V	W	N	-906.759
'0301010105030103020101	L	D	I	R	V	L	L	I	A	W	H	-906.757
'0101010201030203040101	V	D	I	H	L	L	A	I	L	W	H	-906.757
'0201010405040101040101	I	D	I	Q	V	M	L	V	L	W	H	-906.756
'0101010102020102030101	V	D	I	R	F	I	L	A	I	W	H	-906.755
'0101020102010203040101	V	D	L	R	F	V	A	I	L	W	H	-906.754
'0301030103010102010109	L	D	M	R	I	V	L	A	V	W	Q	-906.753
'0101010103010203030103	V	D	I	R	I	V	A	I	I	W	F	-906.753
'0101010105030401060105	V	D	I	R	V	L	W	V	S	W	K	-906.749
'0101030102010102020109	V	D	M	R	F	V	L	A	A	W	Q	-906.748
'0301020102010101040106	L	D	L	R	F	V	L	V	L	W	L	-906.746
'0301010201040101040104	L	D	I	H	L	M	L	V	L	W	I	-906.746

**'01010104010201010101 06**	V	D	I	Q	L	I	L	V	V	W	L	-906.744
**'02010401030101040101 11**	I	D	V	R	I	V	L	L	V	W	T	-906.743
**'01010302030101030101 02**	V	D	M	H	I	V	L	I	V	W	E	-906.743
**'01010103030101030501 10**	V	D	I	K	I	V	L	I	M	W	R	-906.742
**'01010101030104030201 10**	V	D	I	R	I	V	W	I	A	W	R	-906.74
**'03010101030401010101 07**	L	D	I	R	I	M	L	V	V	W	M	-906.74
**'02010103050101010101 04**	I	D	I	K	V	V	L	V	V	W	I	-906.738
**'02010101030101040201 01**	I	D	I	R	I	V	L	L	A	W	H	-906.738
**'01010102020401030601 01**	V	D	I	H	F	M	L	I	S	W	H	-906.738
**'01010404050103010101 06**	V	D	V	Q	V	V	V	V	V	W	L	-906.737
**'01010402030103040101 01**	V	D	V	H	I	V	V	L	V	W	H	-906.736
**'01010301020401030101 07**	V	D	M	R	F	M	L	I	V	W	M	-906.735
**'01010103020101010701 12**	V	D	I	K	F	V	L	V	T	W	V	-906.734
**'03010101050201020601 01**	L	D	I	R	V	I	L	A	S	W	H	-906.732
**'02010101040101010101 10**	I	D	I	R	M	V	L	V	V	W	R	-906.731
**'01010301030101050201 06**	V	D	M	R	I	V	L	M	A	W	L	-906.731
**'02010104050102010101 12**	I	D	I	Q	V	V	A	V	V	W	V	-906.73
**'01010101010201010501 06**	V	D	I	R	L	I	L	V	M	W	L	-906.73
**'03010102020401010601 01**	L	D	I	H	F	M	L	V	S	W	H	-906.728
**'01010102030104010101 08**	V	D	I	H	I	V	W	V	V	W	N	-906.728
**'01010201010201010201 06**	V	D	L	R	L	I	L	V	A	W	L	-906.728
**'01010301010101010501 10**	V	D	M	R	L	V	L	V	M	W	R	-906.727
**'01010102010304010201 12**	V	D	I	H	L	L	W	V	A	W	V	-906.726
**'01010102050301050101 01**	V	D	I	H	V	L	L	M	V	W	H	-906.725
**'01010201050101040101 07**	V	D	L	R	V	V	L	L	V	W	M	-906.725
**'01010201020401010301 04**	V	D	L	R	F	M	L	V	I	W	I	-906.725
**'02010401010201030201 01**	I	D	V	R	L	I	L	I	A	W	H	-906.724
**'01010101020101020401 03**	V	D	I	R	F	V	L	A	L	W	F	-906.723
**'01010102010203010401 01**	V	D	I	H	L	I	V	V	L	W	H	-906.723
**'01010102010303010401 04**	V	D	I	H	L	L	V	V	L	W	I	-906.723
**'01010304050201040101 01**	V	D	M	Q	V	I	L	L	V	W	H	-906.722
**'01010203010301010201 01**	V	D	L	K	L	L	L	V	A	W	H	-906.721
**'02010402040401010101 01**	I	D	V	H	M	M	L	V	V	W	H	-906.721
**'02010404030101010101 10**	I	D	V	Q	I	V	L	V	V	W	R	-906.721
**'01010201050201010701 07**	V	D	L	R	V	I	L	V	T	W	M	-906.718
**'01010301030103030701 01**	V	D	M	R	I	V	V	I	T	W	H	-906.718
**'02010401030101010201 09**	I	D	V	R	I	V	L	V	A	W	Q	-906.715

'0101040103010401040108	V	D	V	R	I	V	W	V	L	W	N	-906.714
'0101010102010304030110	V	D	I	R	F	V	V	L	I	W	R	-906.714
'0101040204030101010101	V	D	V	H	M	L	L	V	V	W	H	-906.713
'0301010105030101010112	L	D	I	R	V	L	L	V	V	W	V	-906.712
'0101010304010104050112	V	D	I	K	M	V	L	L	M	W	V	-906.712
'0101030103010103060102	V	D	M	R	I	V	L	I	S	W	E	-906.709
'0101010405010404010112	V	D	I	Q	V	V	W	L	V	W	V	-906.707
'0201010103040101020106	I	D	I	R	I	M	L	V	A	W	L	-906.704
'0101010103040102040101	V	D	I	R	I	M	L	A	L	W	H	-906.703
'0201010405020104010101	I	D	I	Q	V	I	L	L	V	W	H	-906.703
'0201010101030303010104	I	D	I	R	L	L	V	I	V	W	I	-906.699
'0301010102010402010101	L	D	I	R	F	V	W	A	V	W	H	-906.698
'0301040205010101010103	L	D	V	H	V	V	L	V	V	W	F	-906.698
'0101040104020401010109	V	D	V	R	M	I	W	V	V	W	Q	-906.698
'0101010203010301040112	V	D	I	H	I	V	V	V	L	W	V	-906.696
'0101030102030401070101	V	D	M	R	F	L	W	V	T	W	H	-906.696
'0201010105030301010110	I	D	I	R	V	L	V	V	V	W	R	-906.692
'0101010103020401030110	V	D	I	R	I	I	W	V	I	W	R	-906.691
'0201010101030103060108	I	D	I	R	L	L	L	I	S	W	N	-906.69
'0101010105020105030109	V	D	I	R	V	I	L	M	I	W	Q	-906.69
'0301010102010101010103	L	D	I	R	F	V	L	V	V	W	F	-906.689
'0101010304010104030104	V	D	I	K	M	V	L	L	I	W	I	-906.689
'0101010203010103070106	V	D	I	H	I	V	L	I	T	W	L	-906.688
'0201040105040201010101	I	D	V	R	V	M	A	V	V	W	H	-906.688
'0101010101030201010109	V	D	I	R	L	L	A	V	V	W	Q	-906.687
'0101040104010103050104	V	D	V	R	M	V	L	I	M	W	I	-906.687
'0101040105010301050106	V	D	V	R	V	V	V	V	M	W	L	-906.686
'0101040302010401010109	V	D	V	K	F	V	W	V	V	W	Q	-906.685
'0201030103010102010104	I	D	M	R	I	V	L	A	V	W	I	-906.685
'0101040103010102040106	V	D	V	R	I	V	L	A	L	W	L	-906.684
'0101040305010101040103	V	D	V	K	V	V	L	V	L	W	F	-906.684
'0201010103020102020101	I	D	I	R	I	I	L	A	A	W	H	-906.684
'0101010105030403010102	V	D	I	R	V	L	W	I	V	W	E	-906.683
'0201010302040101070101	I	D	I	K	F	M	L	V	T	W	H	-906.682
'0101010402030204010101	V	D	I	Q	F	L	A	L	V	W	H	-906.679
'0301010205010201010110	L	D	I	H	V	V	A	V	V	W	R	-906.676
'0101010101030401010108	V	D	I	R	L	L	W	V	V	W	N	-906.676

**'0201010102010201020112**	I	D	I	R	F	V	A	V	A	W	V	-906.676
**'0101010201010101040106**	V	D	I	H	L	V	L	V	L	W	L	-906.675
**'0201040105010101010102**	I	D	V	R	V	V	L	V	V	W	E	-906.674
**'0101040401020101070103**	V	D	V	Q	L	I	L	V	T	W	F	-906.674
**'0101030301020101070106**	V	D	M	K	L	I	L	V	T	W	L	-906.672
**'0201040101010103040110**	I	D	V	R	L	V	L	I	L	W	R	-906.67
**'0301010105010102030102**	L	D	I	R	V	V	L	A	I	W	E	-906.67
**'0101020305030101010105**	V	D	L	K	V	L	L	V	V	W	K	-906.667
**'0101010201020101060109**	V	D	I	H	L	I	L	V	S	W	Q	-906.665
**'0201030105010101050108**	I	D	M	R	V	V	L	V	M	W	N	-906.665
**'0101010302040301040101**	V	D	I	K	F	M	V	V	L	W	H	-906.665
**'0101010105010205070104**	V	D	I	R	V	V	A	M	T	W	I	-906.664
**'0201010104030203010101**	I	D	I	R	M	L	A	I	V	W	H	-906.664
**'0201010102020301010108**	I	D	I	R	F	I	V	V	V	W	N	-906.661
**'0101020302040101030101**	V	D	L	K	F	M	L	V	I	W	H	-906.66
**'0101040105020201040101**	V	D	V	R	V	I	A	V	L	W	H	-906.658
**'0101010104010101070105**	V	D	I	R	M	V	L	V	T	W	K	-906.657
**'0101020202010101050106**	V	D	L	H	F	V	L	V	M	W	L	-906.655
**'0301010103020301070101**	L	D	I	R	I	I	V	V	T	W	H	-906.655
**'0101030205020401010101**	V	D	M	H	V	I	W	V	V	W	H	-906.651
**'0301010204010101070110**	L	D	I	H	M	V	L	V	T	W	R	-906.649
**'0101010104010104020104**	V	D	I	R	M	V	L	L	A	W	I	-906.648
**'0101010404040101030105**	V	D	I	Q	M	M	L	V	I	W	K	-906.645
**'0101010205010104070102**	V	D	I	H	V	V	L	L	T	W	E	-906.644
**'0101040305020102010101**	V	D	V	K	V	I	L	A	V	W	H	-906.642
**'0201010203010103020101**	I	D	I	H	I	V	L	I	A	W	H	-906.64
**'0101020202010404010101**	V	D	L	H	F	V	W	L	V	W	H	-906.637
**'0101010304010101030107**	V	D	I	K	M	V	L	V	I	W	M	-906.637
**'0101010302020101030109**	V	D	I	K	F	I	L	V	I	W	Q	-906.636
**'0101010101020104040110**	V	D	I	R	L	I	L	L	L	W	R	-906.635
**'0101010104040101070107**	V	D	I	R	M	M	L	V	T	W	M	-906.634
**'0301010201020101030106**	L	D	I	H	L	I	L	V	I	W	L	-906.633
**'0101040102010201050103**	V	D	V	R	F	V	A	V	M	W	F	-906.633
**'0101020101020103010101**	V	D	L	R	L	I	L	I	V	W	H	-906.628
**'0101010202010105060110**	V	D	I	H	F	V	L	M	S	W	R	-906.628
**'0101010103030105010106**	V	D	I	R	I	L	L	M	V	W	L	-906.626
**'0301040402010104010101**	L	D	V	Q	F	V	L	L	V	W	H	-906.626

**'0201040101020101070101**	I	D	V	R	L	I	L	V	T	W	H	-906.625
**'0201010103030104010112**	I	D	I	R	I	L	L	L	V	W	V	-906.625
**'0101030201030101060105**	V	D	M	H	L	L	L	V	S	W	K	-906.625
**'0101040301010103040111**	V	D	V	K	L	V	L	I	L	W	T	-906.622
**'0101030305010101030107**	V	D	M	K	V	V	L	V	I	W	M	-906.618
**'0101040103020101060107**	V	D	V	R	I	I	L	V	S	W	M	-906.618
